# The functional synergism of microRNA clustering provides therapeutically relevant epigenetic interference in glioblastoma

**DOI:** 10.1038/s41467-019-08390-z

**Published:** 2019-01-25

**Authors:** Vivek Bhaskaran, Michal O. Nowicki, Mahmoud Idriss, Miguel A. Jimenez, Gianmarco Lugli, Josie L. Hayes, Ahmad Bakur Mahmoud, Rachel E. Zane, Carmela Passaro, Keith L. Ligon, Daphne Haas-Kogan, Agnieszka Bronisz, Jakub Godlewski, Sean E. Lawler, E. Antonio Chiocca, Pierpaolo Peruzzi

**Affiliations:** 1 Harvey Cushing Neuro-Oncology Laboratories, Department of Neurosurgery, Harvard Medical School and Brigham and Women’s Hospital, Boston, MA 02115 USA; 2 Northeastern University, Boston, MA 02115 USA; 3 University of Central Florida, Orlando, FL 32816 USA; 4 Facolta’ di medicina e chirurgia, Universita’ Cattolica del Sacro Cuore, Rome, 00168 Italy; 5 School of Public Health, University of California, Berkeley, Berkeley, CA 94720 USA; 6 College of Applied Medical Sciences, Taibah University, Madinah, 42353 Saudi Arabia; 7 Division of Neuro-Pathology, Department of Pathology, Brigham and Women’s Hospital, Boston, MA 02115 USA; 8 Department of Radiation Oncology, Harvard Medical School, Dana-Farber Cancer Institute, Boston, MA 02115 USA

**Keywords:** Gene therapy, Cancer therapeutic resistance, CNS cancer, miRNAs

## Abstract

MicroRNA deregulation is a consistent feature of glioblastoma, yet the biological effect of each single gene is generally modest, and therapeutically negligible. Here we describe a module of microRNAs, constituted by miR-124, miR-128 and miR-137, which are co-expressed during neuronal differentiation and simultaneously lost in gliomagenesis. Each one of these miRs targets several transcriptional regulators, including the oncogenic chromatin repressors EZH2, BMI1 and LSD1, which are functionally interdependent and involved in glioblastoma recurrence after therapeutic chemoradiation. Synchronizing the expression of these three microRNAs in a gene therapy approach displays significant anticancer synergism, abrogates this epigenetic-mediated, multi-protein tumor survival mechanism and results in a 5-fold increase in survival when combined with chemotherapy in murine glioblastoma models. These transgenic microRNA clusters display intercellular propagation in vivo, via extracellular vesicles, extending their biological effect throughout the whole tumor. Our results support the rationale and feasibility of combinatorial microRNA strategies for anticancer therapies.

## Abstract (web summary)

The delivery of single therapeutic microRNAs in brain cancers is challenging. Here, the authors engineer three neuronal microRNAs (miR-124, 128 and 137) into a cluster that, targeting oncogenic chromatin repressors, increases survival of GBM-bearing mice when combined with chemotherapy.

## Introduction

Since the initial description of their role in the pathogenesis of cancer in 2002^1^, microRNAs have been extensively studied in several human malignancies, including brain tumors, and many of them have been established as important players in cancer biology, by either facilitating or hampering tumor development^2–5^. Yet, to date, only two clinical trials have been reported, describing the use of microRNAs for the treatment of cancers, none of them involving glioblastoma (GBM) patients. With only partial responses and some evidence of toxicity, the results of these trials point to the need for further improvements^6,7^. Among the challenges in applying microRNAs as cancer therapeutics are: (1) the intratumoral heterogeneity, biological complexity and numerous aberrancies of cancer cells are highly unlikely to be targeted by a single microRNA of choice; (2) single microRNAs usually achieve a significant, but rarely meaningful, biologic effect in cancer cells; (3) inefficient in vivo delivery, especially for brain cancers, dilutes anticancer effects that were observed in vitro. The existence of genetically determined microRNA clusters, i.e. DNA loci of various length encoding several microRNAs in tandem^8–10^, suggests the gregarious nature of microRNAs and its functional importance. We thus hypothesized that the clustering properties of microRNAs could be exploited for the development of a novel and more effective gene therapy approach against GBM and other cancers. This hypothesis takes advantage of microRNAs’ small size (~70–100 nucleotide long in the precursor form and only ~22 nucleotide long in the mature, active form), their rather simple and widely conserved biogenesis mechanism^11^, and their striking propensity to be shed by tumor cells via extracellular vesicles (EVs)^12^ or gap junctions^13^. In this report, we show that several microRNAs implicated in GBM pathobiology display a pattern of clustered expression, even if not physically encoded in the same genetic locus; that is, they are coexpressed during specific normal homeostatic cellular programs, but are consistently downregulated together in GBM. We then show that the combined re-expression of these microRNAs from an artificially engineered cluster is biologically synergistic when compared to isolated microRNA overexpression. We propose that this artificial microRNA cluster works through targeting vital epigenetic pathways crucial for GBM growth and survival responses to genotoxic stress. This engineered microRNA cluster was actively transferred among tumor cells via EVs, both in vitro and in vivo, resulting in an effective gene therapy in a GBM mouse model.

## Results

### Identification of functional microRNA modules in GBM

**Fig. 1:**
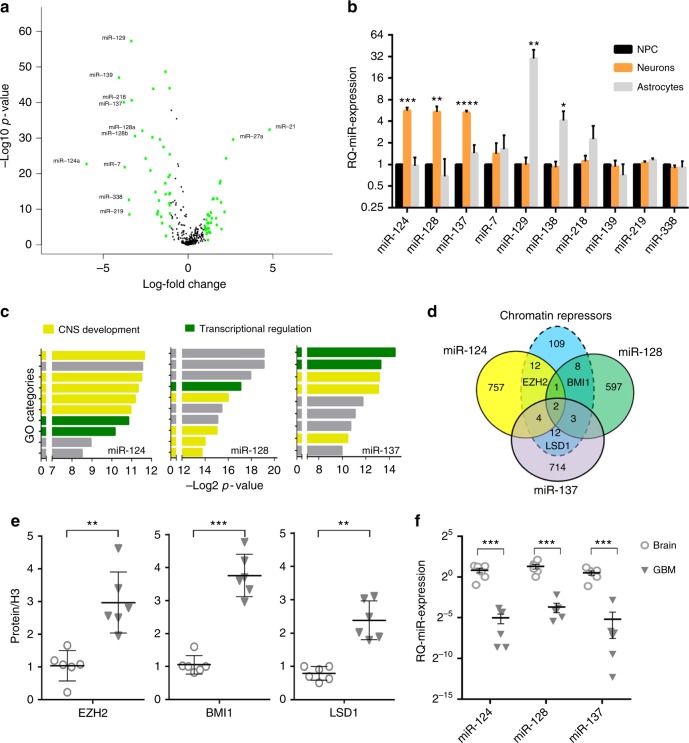
A neuronal microRNA module targets multiple chromatin modifying proteins in GBM. **a** Volcano plot showing the most deregulated microRNAs in glioblastomas (*n* = 520) vs. normal brain (*n* = 10). Green color = >4-fold change in expression. **b** Real-time quantitative PCR analysis of microRNA expression in human neural progenitor cells (NPC) upon induction of lineage-specific differentiation. Mean ± SD from three biological replicates. **c** Schematic representation of the ten most enriched GO categories among the predicted targets of miR-124, miR-128, and miR-137, respectively. Yellow color denotes genes with involvement in neural development. Green color denotes genes with involvement in transcriptional regulation. Gray color denotes any other biological process. **d** Venn diagram crossing the predicted targetome of each microRNA against the group of genes with repressive chromatin function according to GO analysis. **e** Semiquantitative protein quantification of western blot analysis from operatory specimen lysates. For each protein, all samples were equalized to the expression level of Normal Brain #1. **f** Relative quantification of microRNA expression in clinical samples of GBM and brain by real-time PCR. All samples were equalized to the expression level of Normal Brain #1.*p < 0.05; **p < 0.01; ***p < 0.001; ****p < 0.0001 (Student’s *t* test, two tails). GBM gliobastoma

The Cancer Genome Atlas (TCGA) database was queried for the differential expression of all annotated microRNAs between GBMs (*n* = 558) and normal brain (*n* = 10) (Supplementary Data 1). The microRNAs segregated into GBM-downregulated and GBM-upregulated, as shown in the Volcano plot in Fig. 1a. The ten most profoundly and significantly downregulated microRNAs appeared not to show variability according to known transcriptional GBM subtypes^14^ (Supplementary Fig. 1), suggesting that they may represent a core signature of the tumor and its underlying basic biology. We hypothesized that some, or all of these selected microRNAs could be functionally connected. To test this, we first measured the change in their expression in neural progenitor cells (NPCs) during differentiation in vitro. There was clustered expression of specific combinations of these ten microRNAs when NPCs were induced to differentiate either towards a neuronal (upregulation of miR-124, miR-128, and miR-137) or astrocytic lineage (upregulation of miR-129 and miR-138) while we did not observe significant changes for the remaining five microRNAs (Fig. 1b).

We had previously published that miR-128 re-expression led to anticancer effect in GBM cells and that this was mediated by its downregulation of the chromatin repressors BMI1 and SUZ12^15,16^. Because of this, and since differentiation is associated with changes in epigenetic modifiers^17^, we investigated whether the other microRNAs of the neuronal cluster (i.e. miR-124 and miR-137) regulated other proteins functionally related to miR-128 targets and with chromatin repressor function. Gene Ontology analysis (www.toppgene.cchmc.org)^18^ of the predicted targetome (www.targetscan.org)^19^ of each microRNA (detailed in Supplementary Data 2–4) revealed a strong enrichment for the neurogenesis and transcriptional regulation categories (Fig. 1c and Supplementary Table 1), with each microRNA showing potential targeting of several chromatin-associated proteins (Supplementary Data 5 and 6). Among miR-124 targets, we focused on EZH2, a known GBM oncogene^20^ which is a functional partner of BMI1^21^. Both EZH2 and BMI1 were recently shown to be promising candidates for co-inhibition in GBM^22^. Similarly, KDM1A (LSD1) was chosen among miR-137 targets, because of its important role as one of the master epigenetic proteins involved in GBM stemness^23^ and its known functional interaction with EZH2^24^ (Fig. 1d). For all three microRNAs, the targeting specificity had previously been validated by us and others: miR-128 targets BMI1^15^, miR-124 targets EZH2^25^, and miR-137 targets LSD1^26^. These three proteins showed a reduction in expression after induction of neural differentiation but not after astrocytic differentiation of NPCs (Supplementary Fig. 2).

Six primary GBM operative specimens were analyzed against six normal brains for the quantification of the three proteins (Fig. 1e and Supplementary Fig. 3) and microRNAs (Fig. 1f). This analysis showed that BMI1, EZH2, and LSD1 were simultaneously upregulated in GBM while the three corresponding microRNAs were consistently downregulated. Lentiviral-mediated overexpression of each microRNA in primary glioblastoma stem-like cells (GSCs) did not induce changes in expression in the other two microRNAs (Supplementary Fig. 4A, C), and resulted in downregulation of only their known respective targets (Supplementary Fig. 4B,D), without overlaps. These findings supported the hypothesis that miR-128, miR-124, and miR-137 function independently but cooperatively, each downregulating the expression of distinct targets (BMI1, EZH2, and LSD1, respectively) within a defined chromatin-repression module.

### A coordinated GBM response to therapy

**Fig. 2:**
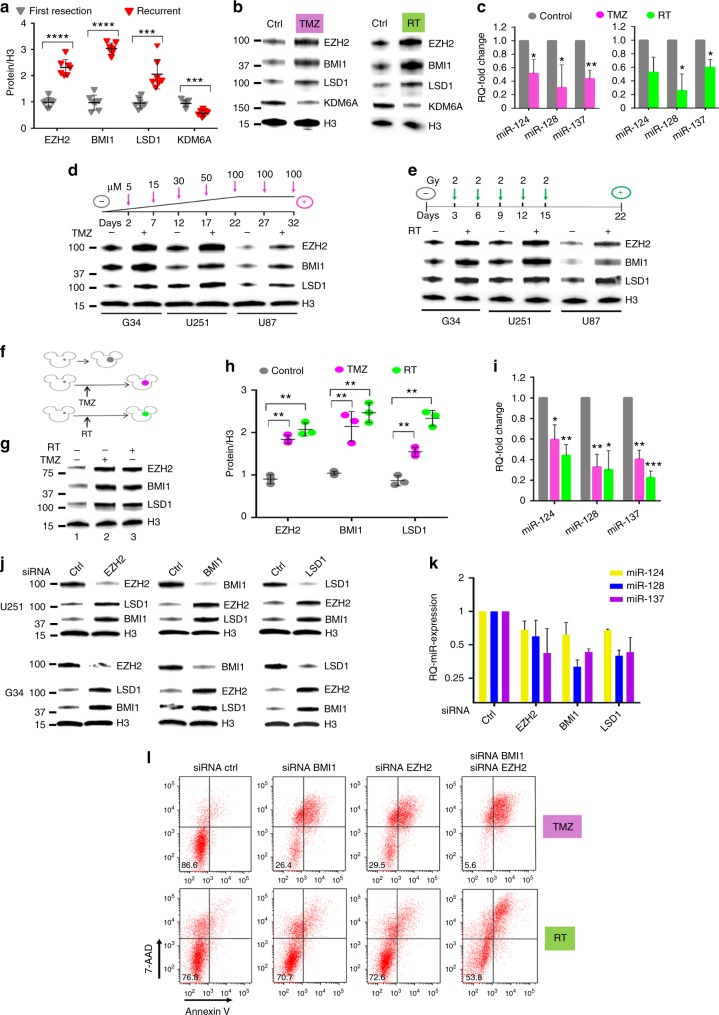
Epigenetic-mediated, multiprotein-enacted GBM escape from genotoxic therapy. **a** Protein quantification from operatory specimen of glioblastomas at time of first diagnosis vs. after recurrence. For each protein, all samples were equalized to the expression level of First Resection sample #1. **b** Protein expression of G34 cells treated with either 15 μM TMZ or 2 Gy of ionizing irradiation, and harvested 24 h after treatment. One representative experiment is shown. **c** Relative quantification of microRNA expression in G34 cells treated as in **b**. Mean ± SD from three independent experiments. **d** Protein expression analysis of three different GBM cell lines treated with progressively increasing concentration of TMZ over 5 weeks, or **e** repeated radiation treatment, as schematized by each corresponding cartoon. **f** Cartoon exemplifying in vivo experiment: tumors grown after the treatment are color-coded: violet denotes tumor after TMZ, green denotes tumor after radiation. Untreated tumors are colored in gray. **g** Representative western blot comparing protein expression from intracranial tumors recovered at the time of mouse euthanasia, either without treatment (mouse 1), after TMZ (mouse 2) and after radiation therapy (mouse 3). **h** Protein quantification from mice in **f** and **g**. **i** Relative quantification of microRNA expression from tumors in **f** and **g**. All samples were equalized to the expression level of control mouse #1. **j** Western blot showing protein level after siRNA-mediated inhibition of specific epigenetic proteins in two different GBM cell lines. **k** Relative quantification of microRNA expression after siRNA knockdown in G34 cells. **l** FACS analysis of cell death and apoptosis after single or double siRNA knockdown of BMI1 and/or EZH2 in U251 cells 24 h after treatment with either 15 μM TMZ (upper row) or 2 Gy of ionizing radiation (lower row). Percent of living cells is reported in each left lower quadrant. 7-AAD 7-amino-actynomicin-D. For all panels, reported are mean ± SD. *p < 0.05; **p < 0.01; ***p < 0.001; ****p < 0.0001 (Student’s *t* test, two tails). GBM glioblastoma, TMZ temozolomide

EZH2 becomes upregulated in recurrent GBMs when compared to tumors at initial diagnosis^27^, and it has a role in DNA protection from genotoxic stress^27,28^. BMI1 has also been shown to facilitate DNA repair through recruitment of the DNA damage response to break sites^29^. We thus analyzed BMI1, EZH2, and LSD1 protein levels in seven recurrent GBM tumors and compared them to GBMs at time of first resection (before adjuvant treatment). All three proteins were significantly upregulated in recurrent tumors (Fig. 2a). Importantly, KDM6A, a H3K27 de-methylase that functions in opposition to EZH2^30^, was downregulated, confirming the specificity of the observed pattern. Since all recurrent tumors had previously been treated with both temozolomide (TMZ, a DNA alkylating agent) and irradiation (radiation therapy, RT), we investigated whether these changes in EZH2, BMI1, LSD1, and KDM6A could result from the therapy itself. To test this, six different GBM cell lines were treated in vitro with either TMZ or RT and analyzed at 24 h for expression of the four proteins (Fig. 2b and Supplementary Fig. 5A) as well as of the three microRNAs (Fig. 2c and Supplementary Fig. 5B). In all cases, increased expression of EZH2, BMI1, and LSD1, with reduction in expression of the three microRNAs was observed. Also, KDM6A was downregulated, recapitulating the observation obtained from operative specimens.

Next, we asked if this was associated with a survival response by tumor cells: three GBM cell lines were either cultured in progressively increasing concentrations of TMZ (5−100 µM over 5 weeks), or repeatedly irradiated (2 Gy every 3 days for 5 sessions over 2 weeks). Cells that survived this treatment were resistant to rechallenge with TMZ or RT (Supplementary Fig. 6) and displayed overexpression of BMI1, EZH2, and LSD1 in comparison to untreated controls (Fig. 2d, e). To investigate if this was recapitulated in vivo, G34 cells, an aggressive patient-derived GSCs, were intracranially implanted in the brain of nude mice and then were treated either with intraperitoneal TMZ or focal RT (Fig. 2f). When mice eventually succumbed, the tumor was isolated from the brains and the amount of BMI1, EZH2, and LSD1 proteins in the treated tumors was compared against those of untreated tumors, confirming a significant increase for all three proteins (Fig. 2g, h). Also, miR-124, miR-128, and miR-137 showed further downregulation in cells that had received the genotoxic treatment (Fig. 2i), confirming an inverse correlation with their target proteins.

We then considered the possibility that the three proteins may act as partners in regulating this stereotypical biological response: siRNA-mediated downregulation of each one of the three proteins individually led to a consistent and marked upregulation of the other two (Fig. 2j) mainly by transcriptional activation (Supplementary Figs. 7 and 8), while microRNA levels did not change significantly (Fig. 2k). To test for a possible functional redundancy among the three proteins, either TMZ or RT were administered after siRNA knockdown of BMI1 or EZH2 or both combined. While there was low to moderate toxicity with single gene interference, there was marked induction of cell death when the two proteins were targeted simultaneously, confirming redundancy of activity (Fig. 2l). The effect on cell death by protein knockdown (either single or multiple) was not significant without genotoxic stress (Supplementary Fig. 9), confirming our observation that this response is important in the setting of DNA damage.

### Synergistic antitumor activity of clustered microRNAs

**Fig. 3:**
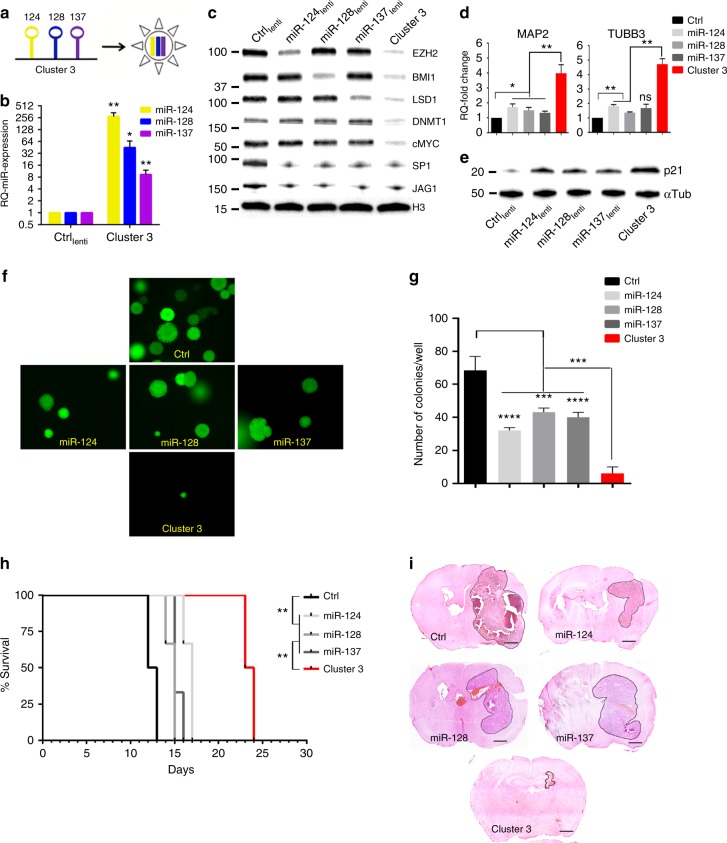
Biological effects of microRNA clustering. **a** Cartoon depicting the engineering of Cluster 3. **b** Relative quantification of microRNA expression in G34 GSC transduced with Cluster 3 transgene or negative control (GFP-only transgene). **c** Representative Western blot from whole cell protein lysate of G34 GSC stably expressing different microRNAs. **d** Relative quantification of MAP2 and TUBB3 (β3-tubulin) gene expression by quantitative real-time PCR after microRNA overexpression. **e** Western blot from G34 GSC showing p21 protein level after microRNA overexpression. **f** Soft agar clonogenic assay. Representative images of GFP-positive G34 cells 14 days after plating into 12-well plates (1000 cells/well). Image acquired with ×4 optical lens (4 × 4 tile scanning). **g** Total count of colonies in **e**. **h** Kaplan−Meier survival curve of female athymic nu/nu mice intracranially implanted with 10,000 G34 GSC stably expressing the indicated microRNAs (or negative control GFP). Six mice/group. Log Rank test, corrected by Bonferroni analysis. **p < 0.001. **i** Representative hematoxylin and eosin stain of paraformaldehyde-fixed brains from mice in **c**. All brains were collected at day 12 post-tumor implantation. Scale bars: 1 mm. For all bar graphs, means ± SD from three independent experiments are reported. *p < 0.05; **p < 0.01; ***p < 0.001; ****p < 0.0001 (Student’s *t* test, two tails, multiple comparisons). GSC glioblastoma stem-like cell

To study the combined effect of microRNAs, a transgene encoding miR-124/128/137 within a polycistronic RNA sequence (hereafter called Cluster 3) was cloned into a lentiviral vector (Fig. 3a), and was used to establish various GBM lines stably expressing the microRNAs (Fig. 3b and Supplementary Fig. 10A). A lentiviral vector encoding only the GFP transgene and a scrambled Cluster 3 sequence were used as negative controls. When microRNAs were upregulated via this engineered cluster, not only were all their target proteins (BMI1, EZH2, and LSD1) downregulated, but there was also a significant downregulation of DNMT1 and MYC (Fig. 3c and Supplementary Fig. 10B) which were otherwise not affected by expression of the single miRs. DNMT1 and MYC are two important oncogenes well described in GBM pathobiology, shown to function in association with EZH2^31,32^ and their expression appears to be dependent on the combined activity of EZH2, BMI1, and LSD1 (Supplementary Fig. 11). Additionally, the protein expression of SP1 and JAG1, whose mRNAs are targeted simultaneously by miR-124, miR-128, and miR-137^18,33^, was quantified. In this case, we did not observe incremental decrease in protein level with the full cluster as opposed to the single miRs, suggesting that the biological effect of clustered microRNAs is to expand the targetome rather than to potentiate the effect on common targets (Fig. 3c).

At the functional level, clustered microRNA overexpression resulted in increased expression of neuronal genes MAP2 and TUBB3 (Fig. 3d and Supplementary Fig. 10C). Also, the microRNA cluster upregulated p21 (a gene known to be independently silenced by EZH2^34^, BMI1^35^, and LSD1^36^ significantly more than single microRNA re-expression (Fig. 3e and Supplementary Fig. 10D). This molecular effect was paralleled by a similarly augmented antitumor effect, both in vitro and in vivo: primary GSCs overexpressing clustered microRNAs exhibited a significant reduction in clonogenicity and proliferation (Fig. 3f, g and Supplementary Fig. 10E), and significant extension of survival in an orthotopic intracranial mouse model (Fig. 3h). Yet, all mice eventually succumbed to the tumor: as shown in Fig. 3i, it appears that cells expressing the microRNAs clusters had produced a very small tumor by the time the control mice died (day 12). Tumor progression after that time point appeared mainly due to progressive loss of microRNA cluster expression in vivo, likely due to CMV promoter silencing (Supplementary Fig. 12).

### MicroRNA clustering abates GBM escape from genotoxic stress

**Fig. 4:**
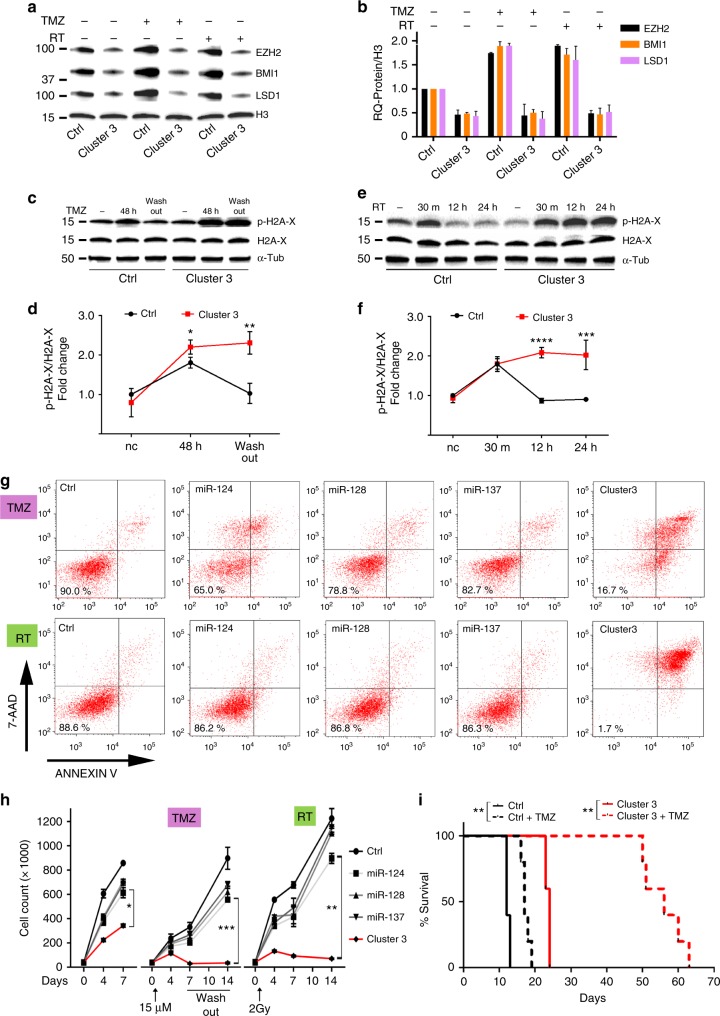
Clustered microRNAs impede GBM escape from genotoxic stress. **a** Western blot from whole-cell lysate of G34 expressing either negative control or Cluster 3 transgene 24 h after either 15 μM TMZ or 2 Gy ionizing radiation. **b** Quantification of proteins from **a**. Values reported represent mean + SD from two separate experiments. **c**, **d** Representative western blots for phospho-H2A-x and H2A-x in G34 GSC after 48 h incubation with 15 μM TMZ and further 48 h after TMZ washout, and relative protein quantification (mean + SD, *n* = 3) **e**, **f** Representative western blot for phospho-H2A-x and H2A-x in G34 GSC 30 min, 12 h and 24 h after 2 Gy irradiation, and relative protein quantification based on three independent experiments (reported values are mean ± SD). **g** FACS-based analysis of cell death and apoptosis in G34 GSC expressing either single miRs or Cluster 3 transgene, in the presence of either 15 μM TMZ (upper row) for 5 days or 5 days after treatment with 2 Gy of ionizing radiation (lower row). Percent of living cells is reported in each left lower quadrant. 7-AAD 7-amino-actynomicin-D. **h** Cell count per well of G34 GSCs expressing different microRNAs at different time points and in different genotoxic conditions (Left panel: no additional treatment; Central panel: TMZ; Right panel: radiation). Reported is the mean ± SD from three independent experiments. For all graphs, *p < 0.05; **p < 0.01; ***p < 0.001; ****p < 0.0001 (Student’s *t* test, two tails). **i** Survival curve of nude mice intracranially implanted with 10,000 G34 GSC differentially expressing clustered microRNAs, and with/without 5 days treatment with 20 mg/kg IP TMZ starting at day 7 post implantation (six mice/group). **p < 0.01, Log Rank corrected by Bonferroni analysis. GSC glioblastoma stem-like cell

To overcome the translational drawback imposed by progressive transgene loss, we asked if Cluster 3 expression during chemotherapy or radiation would augment their anticancer efficacy. As expected, BMI1, EZH2, and LSD1 levels increased significantly after treatment with either TMZ or RT in GSCs expressing negative control (GFP); however, GSCs transduced with the Cluster 3 microRNAs did not increase expression of these three proteins (Fig. 4a, b). This was accompanied by a significant decrease in the clearance of phospho-Histone2A-x accumulation at different time points after treatment, suggesting impaired DNA repair (Fig. 4c–f). The functional relevance of this observation was determined by treating G34 cells expressing single miR-124, miR-128, miR-137 or Cluster 3 with TMZ or RT. There was a modest effect on the proliferation of cells expressing single microRNAs; however, there was a much more significant effect for cells expressing Cluster 3 (Fig. 4h, left panel). After exposure to TMZ or RT, cells expressing single microRNAs continued to proliferate throughout the treatment, while cells expressing Cluster 3 displayed a significant decrease in cell number. These cells could not recover after TMZ washout or as long as 14 days after radiation (Fig. 4h, center and right panels). To verify that the decrease in cell number observed with Cluster 3 was due to cell death and not due to decreased proliferation, cells were collected 5 days after TMZ or RT and were analyzed for the quantification of apoptosis/necrosis by Annexin V/7-AAD staining. The induction of cell death by single microRNA was minimal when compared to Cluster 3 (Fig. 4g). Single or Cluster 3 microRNA expression without TMZ or RT did not induce cell death (Supplementary Fig. 13). Finally, 24 athymic nu/nu mice were intracranially implanted with GSCs expressing either a negative control transgene or the Cluster 3 transgene and were either treated with IP TMZ or vehicle for 5 days, starting 7 days after tumor implantation. Confirming the in vitro data, TMZ treatment resulted in a median survival benefit of 5 days in mice harboring control cells, while the survival benefit significantly increased to 32 days (a >6-fold increase) in animals implanted with cells overexpressing the microRNA cluster (Fig. 4i).

### MicroRNA clusters are transferred to bystander cells via EVs

**Fig. 5:**
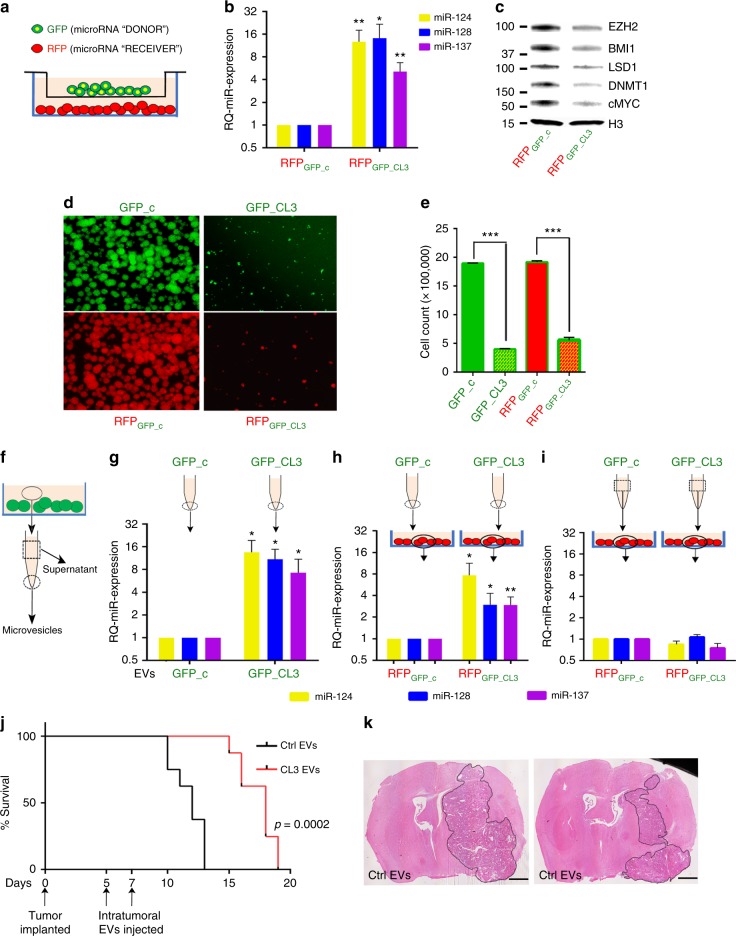
Vesicle-mediated transfer of clustered transgenic microRNAs. **a** Cartoon depicting transwell assay used for microRNA transfer analysis. **b** Relative quantification of microRNA expression by RT PCR in cocultured cells. Reported are means ± SD from three independent experiments. **c** Representative western blot analysis of proteins in RFP+ cells. **d** Representative fluorescent microscope images of G34 neurospheres in different co-culturing conditions, with relative cell count/12-well reported in **e** as mean values ± SD from three experiments. **f** Cartoon depicting the processing of conditioned medium from GFP-positive cells into microvesicular component (dotted circle) and supernatant (dotted square), as used in the following panels. **g** Real-time PCR quantification of microRNA expression in EVs recovered from conditioned medium. **h** microRNA expression from RFP-positive cells cultured for 36 h with 10 µg/ml of purified EVs, or, **i** 2 ml of conditioned medium deprived of EVs (supernatant) obtained from GFP-positive cells. **j** Survival of mice after intratumor injection of EVs (10 μg total by protein quantification delivered in two injections) purified either from negative control cells (ctrl EVs) or Cluster 3 cells (CL3 EVs). *n* = 6 mice per group. **k** Hematoxylin & Eosin stains of representative brains at time of control mouse endpoint. Scale bars: 1 mm. Represented are means ± SD from three independent experiments. *p < 0.05; **p < 0.01; ***p < 0.0001 (Student’s *t* test, two tails). EV extracellular vesicle

We observed that RFP-transduced cells growing in mixed cultures with GFP-positive cells overexpressing the Cluster 3 transgene also showed delayed growth, had higher levels of miR-124, miR-128, and miR-137 and decreased amounts of their protein targets (Supplementary Fig. 14). To investigate if this was the result of an active microRNA transfer among cells, and if cell-to-cell contact was necessary for this transfer, we co-cultured RFP and GFP cells separated by a semipermeable membrane with a 1 µm pore size, to allow free passage of soluble factors and extracellular vesicles (EVs,) but no cell−cell interaction (Fig. 5a). After 5 days of co-culture, there was a several fold increase in miR-128, miR-124, and miR-137 levels in the RFP-positive cells co-cultured with GFP-positive cells expressing the Cluster 3 transgene, but not in those co-cultured with control GFP cells (Fig. 5b). This also corresponded to decreased levels of BMI1, EZH2, LSD1, DNMT1, and MYC (Fig. 5c) and to impaired growth rate (Fig. 5d, e). Importantly, co-culturing with GBM cells expressing only single microRNAs demonstrated a negligible antiproliferative effect on receiving RFP cells, further confirming the importance of combined microRNA modulation to attain a relevant bystander effect (Supplementary Fig. 15). To exclude the possibility that this phenomenon could be due to unwanted virus contamination of the RFP cells from the GFP-positive cells, the transwell experiment was also repeated using plasmid transfection of Cluster 3 or negative control transgenes instead of lentiviral infection, yielding similar results (Supplementary Fig. 16).

MicroRNAs have been reported to be released from cells within EVs, including exosomes shed by tumor cells^12^. We thus collected EVs from GFP-positive cells by ultracentrifugation (Fig. 5f), verified the purity of the preparation, as well as the absence of virus contamination (Supplementary Fig. 17) and confirmed by qPCR that EVs isolated from GSCs expressing the Cluster 3 transgene contained a significantly higher amount of all three microRNAs, in comparison to EVs from negative control cells (Fig. 5g). These EVs were then administered to a culture of RFP- positive cells, and cellular RNA was obtained after 36 h. Quantitative PCR showed a significant increase in the three microRNAs in RFP cells receiving EVs from GFP cells expressing the Cluster 3 transgene (Fig. 5h), while no increase in microRNAs was detected by use of EV-depleted supernatants (Fig. 5i). The observed increase in miR-124, miR-128, and miR-137 was not due to transcriptional activation of their respective genes in the RFP cells (Supplementary Fig. 18). Furthermore, inhibition of EV secretion by RAB27A knockdown^37^ in GFP-positive cells abrogated this microRNA transfer (Supplementary Fig. 19). Finally, direct inoculation of purified EVs into intracranial tumors in mice resulted in a significant increase in survival in animals receiving EVs isolated from Cluster 3 cells (Fig. 5j, k).

### Transfer of microRNA clusters occurs in vivo

**Fig. 6:**
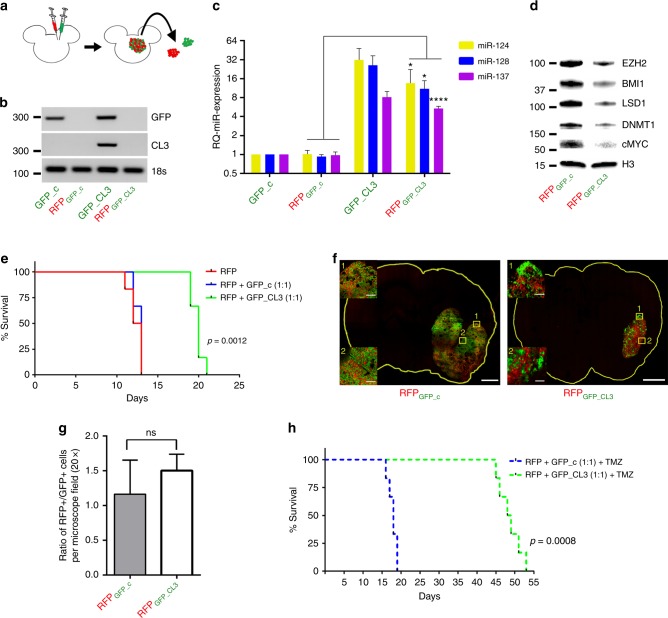
In vivo evidence of microRNA transfer to bystander cells. **a** Scheme of experimental protocol. Red = RFP; Green = GFP. **b** Semiquantitative PCR of Cluster 3 and GFP transgene expression across the four different sorted cell populations. **c** Relative quantification of microRNA expression in G34 cells recovered from mixed intracranial brain tumors at time of mouse euthanasia (day 12). *n* = 3 animals/group. **d** Representative western blot analysis of protein amount in RFP control cells vs. RFP-Cl3. **e** Survival curve of athymic nu/nu mice (6 per group) intracranially implanted with RFP/GFP control vs. RFP/GFP-Cluster3 mixed in a 1:1 ratio. **f** Confocal microscopy image of one representative brain per group sacrificed at day 12, showing the two cell populations, at low magnification and ×20 magnification. Scale bars: 1 mm (whole slide); 100 µm (inserts). **g** Ratio of RFP+/GFP+ cells per ×20 microscopy field (mean ± SD from nine random fields/tumor). **h** Survival curve of athymic mice (six per group) as described in **e**, with the addition of 20 mg/kg IP TMZ for 5 days starting at day 7 post implantation (Student’s *t* test, two tails). *p < 0.05; ****p < 0.0001

To determine if transfer of the microRNAs expressed by Cluster 3 also occurs in vivo, athymic mice were co-implanted intracranially with RFP-positive G34 cells together with an equal number of GFP-positive G34 cells either expressing negative control or the Cluster 3 transgene. As an additional control, three mice were implanted with RFP-positive cells only. When the control mice became symptomatic (day 12), three animals per group were euthanized and the tumors were isolated and enzymatically dissociated into single cells, which were then sorted into the original GFP and RFP components by FACS (Fig. 6a). Purity of the sorted populations was confirmed by PCR amplification of GFP and Cluster 3 transgenes (Fig. 6b). RFP-positive cells showed a significant increase in the three microRNAs in the mice where they were co-injected together with GFP cells expressing Cluster 3 (Fig. 6c). Similarly, there was downregulation of the epigenetic protein module controlled by the three microRNAs (Fig. 6d). Interestingly, we were not able to observe the transfer of microRNAs from normal brain cells to tumor cells in vivo (Supplementary Fig. 20), suggesting preferential transfer among tumor cells.

Survival studies demonstrated that mice co-implanted with RFP and GFP-Cluster 3 cells lived significantly longer than those with RFP and GFP-negative control (Fig. 6e). Importantly, we observed that mice harboring only RFP cells died at approximately the same time as RFP/GFP-negative controls animals, indicating that the prolonged survival observed in the RFP/GFP-Cluster3 mice was not only due to the expected slower growth of GFP cells transduced with Cluster 3 transgene, but also by active growth inhibition of RFP cells exerted by transferred microRNAs, as previously observed in vitro. This was also confirmed by confocal microscopy analysis of the mixed cell population in the tumors. This showed not only a smaller tumor in the RFP/GFP-Cluster 3 mouse, but also that the RFP cells had not significantly outgrown GFP cells, suggesting that the growth of the RFP population had been inhibited (Fig. 6f, g).

With the addition of TMZ there was a significant survival benefit (median survival of 18 days for RFP/GFP-control vs. 48.5 days for RFP/GFP-Cluster3) (Fig. 6h), which could not be explained by only an effect of TMZ on the GFP component, since the RFP component, if not affected by the microRNAs, would have responded only for 5−6 days, as shown in Fig. 4i. Thus, this Cluster 3 microRNA expression approach works not only in infected cells, but also in neighboring uninfected cells by EV-mediated microRNA transfer.

## Discussion

The goal of this work is to provide a wider perspective for the use of microRNAs in cancer therapy, taking advantage from the combination of their several unique features and circumventing their limitations.

In this report, we have identified a module of coexpressed microRNAs which allows repression of epigenetic oncogenic signaling pathways. This prevents compensatory regulatory mechanisms that normally allow cancer cell survival after genotoxic therapy and leads to profound therapeutic benefit in a GBM preclinical model.

The results of the only two clinical trials to date employing microRNAs mimics as therapeutics (miR-34 in GI cancers^6^ and miR-16 in mesothelioma^7^ suggest that single miR modulation may have limited clinical impact. Hence, our interest in defining groups of microRNAs which could be coordinately responsible for the regulation of complex cellular pathways, thus achieving a more profound biological response. In GBM, Silber et al. were among the first to show that the tumor displayed a specific signature of microRNA deregulation and they described both miR-124 and miR-137 as tumor suppressor microRNAs^38^. A possible functional interconnection between miR-124, miR-128, and miR-137 in neural cells was recently described, showing that the three microRNAs converged on the epigenetic regulation of core neuronal-specific transcripts^33^. However, the concept of microRNA clustering, i.e. to study and harness the function of a group of microRNAs together has not been previously explored from a translational perspective in cancer. Our data confirm prior evidence that upregulation of single microRNAs might produce a sensitizing effect to either irradiation or chemotherapy^16,39^, but also proves that a clustered approach is significantly more effective. This is translationally relevant because, as we show, it is feasible to engineer DNA sequences encoding multiple microRNAs of choice, which can be used to modulate the expression of many microRNAs simultaneously.

A crucial aspect for the success of microRNA-mediated therapy lies in the appropriate selection of strategic targets. We argue that such strategy should aim at broadly interfering with cell biology, rather than focusing on single targets. Epigenetic plasticity has been gaining momentum as a fundamental mechanism allowing cancer cells to maintain a fluid, ever adjustable nature, responsible for their ability to survive pharmacological challenges^40,41^. This notion has been shifting the goal of epigenetically targeted therapies from the more classic induction of differentiation, i.e. as a way to induce blander cancer phenotypes^42^, to the new goal of interfering with cancer cell’s ability to adapt and survive. In keeping with this, our data show that GBM cells upregulate a consistent module of several chromatin modifying enzymes in response to pharmacologic and radiation-induced genotoxic stress. This epigenetic response, thus, appears to be a critical vulnerability that could be exploited for a complementary and synergistic therapeutic approach. A major hurdle for translating this observation into therapy, however, comes from the multitude of proteins involved in this epigenetic machinery. This seemingly overlapping and partially redundant role is probably one of the causes why prior trials using Histone Deacetylase inhibitors (HDACi) in solid cancers, including GBM, have not been successful at prolonging survival^43,44^. Furthermore, recent evidence suggests that co-downregulation of multiple such epigenetic pathways, including BMI1 and EZH2, is needed to obtain a significant biological effect in cancer cells^22,31^. Such level of multitargeting can be ideally and naturally offered by properly combined microRNAs, instead than by multidrug cocktails, which might have limitations in terms of toxicity as well as CNS permeability.

The rationale for the specific selection of three microRNAs used in this work (i.e. miR-124, miR-128, and miR-137), while experimentally justified, is certainly not the only one possible. In fact, we have observed that other clustering patterns were present upon different conditions (e.g. miR-138 and miR-129 appeared to be strongly associated with astrocytic differentiation). We speculate that specific miR clustering patterns would likely result from specific cell stimulations (e.g., in response to drugs, metabolic challenges or inflammation), and they could be used in those circumstances to hamper each specific response for therapeutic purpose.

Finally, the demonstration that transgenic microRNAs clusters can diffuse as a group from cell to cell and affect tumor globally adds an important evidence of translatability to this work: we show that a significant antitumor effect can be achieved without the need to transduce 100% of the tumor. Interestingly, this is possible because the fraction of transduced cells does not die as a consequence of the microRNAs overexpression. Instead, they become producers of microRNAs that, in turn, are shed to other bystander tumor cells, priming them for a more cytotoxic effect by other therapeutic interventions, like cytotoxic drugs or irradiation. As we show here, this transfer of microRNAs in vivo appears to preferentially occur between tumor cells. This observation might be important in implementing strategies to deliver microRNA clusters to tumors, favoring in situ, vector-mediated transduction of tumor cells, as opposed to exogenous administration of synthetic microRNA mimics. In this regard, one major advantage of being able to cluster multiple microRNAs into short DNA sequences (our Cluster 3 transgene is 800 bp) is their compatibility with the packaging limits of virtually any available vectors^45^.

## Methods

### Bioinformatic analysis

Level 3 normalized microRNA Agilent 8 × 15k expression data for GBM were downloaded from the Tissue Cancer Genome Atlas (TCGA) database and the LIMMA R package (functions lmFit and eBayes) was used to perform differential expression between normal brain (*n* = 10) and GBM (*n* = 558) samples. The same was performed for subtypes of GBM: mesenchymal (*n* = 145), proneural (*n* = 82), and classical (*n* = 134).

### Human tissue processing

The collection of human operative specimen was performed in accordance to the Brigham and Women’s Hospital/Dana Farber Cancer Institute (IRB protocol 10-417) and after obtaining informed consent. Frozen surgical specimens of histopathology-confirmed GBMs or normal brains were obtained through the Department of Neuropathology at the Dana Farber Cancer Institute. For proteins, tissues were individually homogenized in RIPA buffer (Thermo Fisher Scientific, Waltham, MA) containing a cocktail of protease inhibitors (Complete, Roche Applied Science, Indianapolis, IN) and lysed by sonication. Total homogenates were used for protein electrophoresis. For RNA extraction, tissues were homogenized in Trizol (Thermo Fisher Scientific) according to the manufacturer’s protocol.

### Gene expression studies

Total RNA was extracted using TRIzol (Life Technologies) according to the manufacturer’s protocol. For microRNA analysis, TaqMan microRNA probe (Life Technologies) was used to detect miR-124, miR-128, miR-137, miR-7, miR-129, miR-138, miR-218, miR-139, miR-219, and miR-338 from the cDNA, synthesized by using TaqMan microRNA real-time (RT) PCR detection kit (Applied Biosystems), following the manufacturer’s protocol. Real-time quantification of microRNAs was performed using TaqMan Universal PCR Master mix (Applied Biosystems) and analyzed using Applied Biosystems Step One Plus 7500 RT-PCR apparatus. U6 snRNA (TaqMan probe) was used as an endogenous internal control. For mRNA analysis, cDNA for RT-PCR was synthesized using iScript cDNA Synthesis Kit (BioRad, Hercules, CA). Analysis of mRNA expression was carried out using Power SYBR Green PCR Master Mix (Applied Biosystems) with primers listed in Supplementary Table 3.

### MicroRNA overexpression

For each transgene, a DNA sequence of 500–800 bp containing the ~70 nucleotide precursor microRNAs, as well as ~250 nucleotides 5′ and 3′ flanking regions was PCR-amplified from genomic DNA derived from human astrocytes, and sequenced for confirmation. For the Cluster 3 transgene, the three precursor microRNAs were combined in a polycistronic DNA sequence, separated by ~100 nucleotide spacing sequences, and flanked by the same ~250 bp regions used for the miR-128 transgene. The Cluster 3 DNA sequence was designed in silico by the authors and obtained as a bacterial plasmid from GeneArt (Life Technologies). All DNA sequences were cloned into the lentiviral vector pCDH-EF1-copGFP vector (System Biosciences, Palo Alto, CA). For negative controls, both the empty vector (i.e. GFP-only) and scrambled Cluster 3 sequences were used. The scrambled vector sequence was obtained by replacing each 20 nucleotide sequence encoding for the mature microRNA of Cluster 3 with a scrambled sequence, computer-generated using the GenScript sequence tool at https://www.genscript.com/tools/create-scrambled-sequence. For each microRNA, scrambled sequences were as follows: miR-128: ATCGTCATTCGATTCACTGGC; miR-124: GCGCAAGACGCAATGTCAGAGT; miR-137: GCTAGTAACTTCGTATATAAGGT (full transgene sequences are provided in Supplementary Table 2). After infection, GSCs stably expressing each transgene were sorted by GFP expression and expanded in cultures.

### Extracellular vesicles isolation and studies

EVs were isolated as previously described^46^, with slight modifications: briefly, 5×10^5^ cells were grown in T175 flask for 5 days in antibiotics-free neurobasal medium supplemented with EGF/FGF and B27. The conditioned media were then collected, cleared from cells and cell debris by centrifugation at 3000 × *g* for 10 min followed by 16,500 × *g* for 20 min and then filtered through a 0.22 μm polyvinylidene fluoride (PVDF) filter. Extracellular vesicles were finally isolated by ultracentrifugation at 100,000 × *g* for 70 min and either used for RNA extraction or resuspended in phosphate buffered saline (PBS) for administration to cell cultures.

QuantiMir kit (System Biosciences) was used to extract total RNA from EVs. The RNA (10 ng per sample) was then retrotranscribed and used for PCR-based quantification of miR-124, miR-128, and miR-137, using custom made TaqMan primers (Life Technology).

For protein preparations, the EVs after ultracentrifugation was washed with PBS and dissolved in RIPA buffer and processed for protein electrophoresis. The protein concentration of each preparation was measured by Bradford assay (BioRad) and was used to equalize gel loading.

### Cell studies

For cell proliferation assays, G34 GSCs were seeded in neurobasal medium at a density of 50,000 cells/well (six-well plate) for a period of 4 and 7 days. At each endpoint, neurospheres were dissociated into single cells with Accutase (Thermofisher Scientific) and counted.

For clonogenic studies, G34 cells were dissociated and resuspended as single cells in stem cell medium containing 0.4% low melting temperature agarose (IBI Scientific, Peosta, Iowa). The cells were seeded at a density of 1000/well in a 12-well plate. After 10 min at room temperature to allow medium gelification, cells were grown at standard parameters (37 °C, 20% O_2_, 5% CO_2_) for 2 weeks to allow time for sphere formation. Images were obtained with a Nikon eclipse Ti motorized fluorescent microscope system, Japan (NIS-Elements 4.2) and number of spheres were counted.

For the in vitro EV treatment, 2×10^5^ G34 cells expressing RFP were cultured in 1 ml of medium and were treated 24 and 48 h after seeding with 2 separate administrations of 5 µg (by protein concentration) EVs isolated from either GFP-control or GFP-Cluster 3 conditioned media. Cells were collected for RNA analysis 12 h after the second EV administration.

For in vitro studies involving TMZ or irradiation, all represented GBM cells were treated with TMZ (Tocris Bioscience, Minneapolis, MN) resuspended in DMSO (Fisher Scientific, Fair Lawn, NJ) at working concentration of 1 mM. DMSO was used for control samples. TMZ was administered at concentrations and for periods of time specified in the main text and respective figures. For irradiation studies, cells were placed into a Cesium 137 irradiator (JL Sheperd and Associates, San Fernando, CA) and subjected to a dose of 2 Gy per each treatment.

### Animal studies

All animal experiments were performed in female, 6- to 8-week-old immunodeficient athymic mice (FoxN1 nu/nu, Envigo, South Easton, MA), in compliance with all relevant ethical regulations applied to the use of small rodents, and with approval by the Animal Care and Use Committees (IACUC) at the Brigham and Women’s Hospital and Harvard Medical School. For intracranial tumor implantation, a stereotactic frame was used to inoculate each animal in the right striatum with (unless otherwise specified) 10,000 cells (resuspended in 2 µl PBS), stably expressing different microRNA transgenes, including GFP control. Mice were euthanized and perfused when they reached their predetermined endpoints, and tissues were recovered for biochemical or histochemical analysis. For mRNA and protein analysis, animals were anesthetized and perfused transcardially with 100 mmol/l of sodium phosphate buffer (pH 7.4), followed by tissue dissection under microscope to isolate tumor tissue. For hematoxylin/eosin (H&E) staining, brains were fixed in 4% paraformaldehyde during perfusion and processed for cryosectioning at 25 μm thickness. Sections were imaged using a confocal microscope Zeiss LSM710.

Temozolomide was administered in vivo by intraperitoneal injection at a concentration of 20 mg/kg for 5 consecutive days starting from the seventh day after intracranial tumor implantation.

Irradiation was administered in vivo using a Small Animal Image-Guided Micro Irradiator (Xstrahal Life Sciences, UK), which provided 10 Gy of focal irradiation to the tumor site in three fractions (4 Gy, 3 Gy, 3 Gy) over 6 days.

For in vivo EVs administration, mice were intratumorally inoculated at day 5 and day 7 after tumor implantation, each time with a total of 5 µg of EVs (by protein quantification) in 5 μl of PBS (for a total of 10 µg EVs) using a stereotactic frame with the same coordinates used to implant the initial tumor.

### In vitro and in vivo cell mixing studies

G34 cells stably overexpressing GFP-Cluster 3 and negative control GFP-CDH were mixed with RFP-expressing G34 cells in a ratio of 1:1 and cultured for 5 days in complete stem cell medium. Cell were then dissociated and FACS-sorted by RFP and GFP into the two initial cell populations. RNA and proteins were obtained as described above for each cell population.

For transwell assay, GFP-positive cells were cultured in a six-well Transwell chamber with a semipermeable, 1 μm pore size floor (Greiner Bio-One, Monroe, NC) over RFP-positive cells within the same culturing medium. RFP and GFP cells were seeded at a 1:1 ratio and were harvested for RNA and proteins 5 days after plating.

For in vivo mixing experiments, a 1:1 ratio of RFP- and GFP-positive G34 cells were injected into the right striatum of athymic nu/nu female mice. For molecular studies, a total of 50,000 cells were injected, to ensure sufficient cell recovery at time of euthanasia. For survival studies, 10,000 cells were implanted, consistently with all prior survival studies reported in the manuscript. For molecular studies, after PBS-only perfusion, the tumor was isolated from the brain, dissociated with Accutase and DNAse and the cells were sorted by FACS into GFP+ and RFP+ populations. For microscopy analysis, one mouse per group was also euthanized 12 days after implantation, perfused with 4% PFA and the brain was cryosectioned. GFP+ and RFP+ cells within the tumor were visualized by confocal microscopy and counted by ImageJ software.

### Data and statistical analysis

Data are expressed as mean ± SD. Statistical analyses were performed using the unpaired two-tailed Student’s *t* test from the GraphPad Prism software. All experiments in vitro were repeated in triplicate. All microscopy-based assays and western blot band intensities were quantified using ImageJ. Multiple *t* test followed by Bonferroni correction were used to test for significance when comparing multiple groups in survival studies. Differences were considered statistically significant at *p* < 0.05.

### Reporting summary

Further information on experimental design is available in the Nature Research Reporting Summary linked to this article.

## Supplementary information

Supplementary Information

Peer Review File

Description of Additional Supplementary Files

Supplementary Data 1

Supplementary Data 2

Supplementary Data 3

Supplementary Data 4

Supplementary Data 5

Supplementary Data 6

Reporting Summary

## Data Availability

The data that support the findings of this study which are not directly available within the paper (and its supplementary information files) will be available from the corresponding author upon reasonable request. This includes DNA sequences of all transgenes used in this study.
