# Supplementary material for: The functional synergism of microRNA clustering provides therapeutically relevant epigenetic interference in glioblastoma
Source: Nat Commun. 2019 Jan 25;10:442. doi: 10.1038/s41467-019-08390-z (PMC6347618; doi:10.1038/s41467-019-08390-z)
Supplement: Supplementary file 1 — Supplementary Information [file 41467_2019_8390_MOESM1_ESM.pdf]

Supplementary Information

The functional synergism of microRNA clustering provides therapeutically relevant epigenetic interference in glioblastoma

Bhaskaran et al.

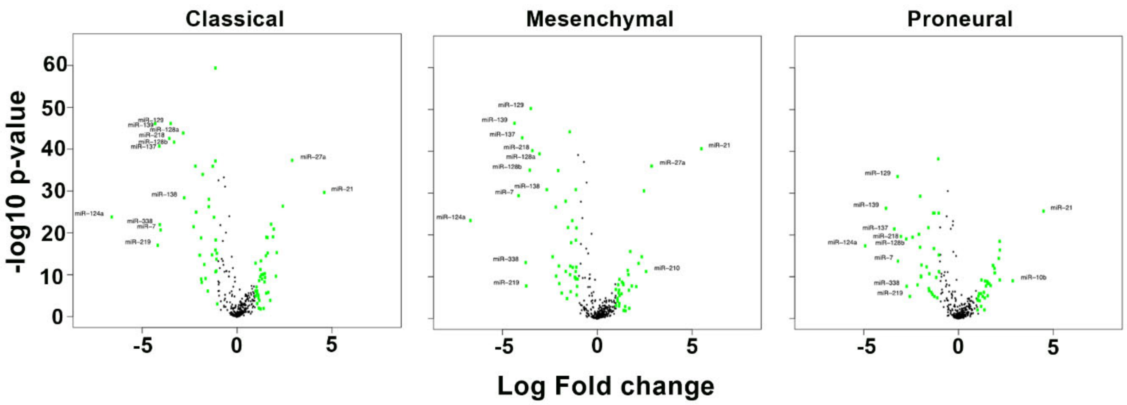

Supplementary Figure 1: Differential microRNA expression across GBM subtypes. Volcano plots from TCGA dataset. Classical subtype (n=134), Mesenchymal subtype (n=145) and Proneural subtype (n=82)

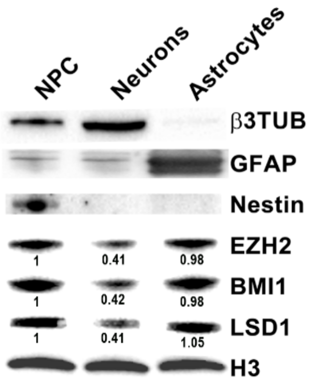

Supplementary Figure 2: Relationship of BMI1, EZH2 and LSD1 protein level with NPC differentiation status. Representative Western blot from human Neural Progenitor Cells grown either in stem cell medium (NPC), or after 7 days in Neuronal differentiation or Astrocytic differentiation conditions. Relative quantification of band intensity is provided underneath each band.

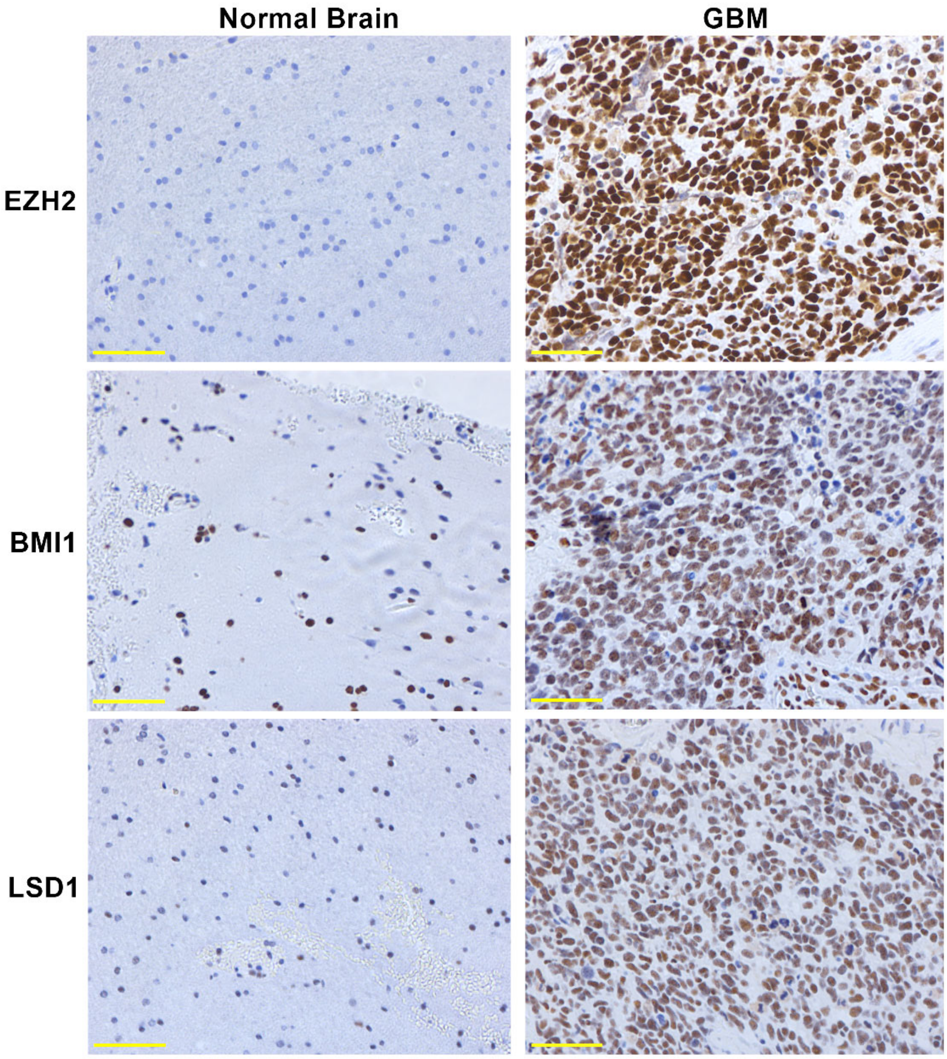

Supplementary Figure 3: Immunohistochemistry for EZH2, BMI1 and LSD1 in operative specimens of normal brain Vs glioblastoma. Cell nuclei are stained with Hematoxilin (in blue) while target proteins are stained with 3,3'-Diaminobenzidine (DAB) (in brown). Magnification: 20x, Scale bars: 100 µm.

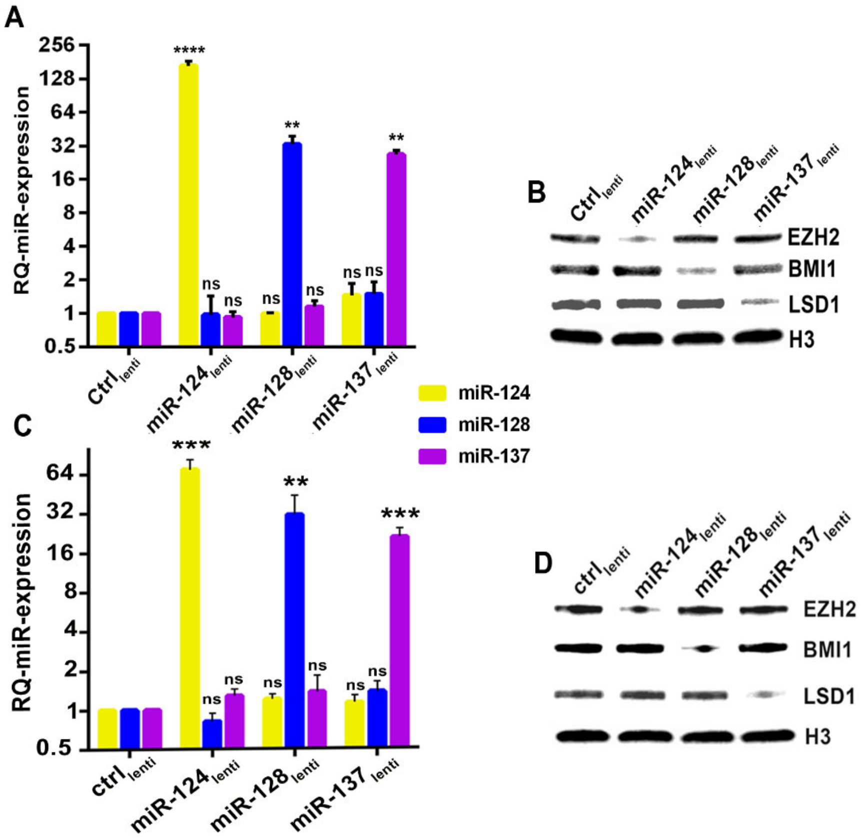

Supplementary Figure 4: Relative quantification of microRNA expression after single microRNA overexpression by lentiviral infection in G34 (**A**) and G62 (**C**) GSC. Mean ± SD form three independent experiments. ** =p<0.01; ***=p<0.001; ****= p<0.0001 (Student's t-test, 2 tails). **B** and **D**: Representative western blot showing the level of EZH2, BMI1 and LSD1 proteins after upregulation of single microRNAs as in A and C.

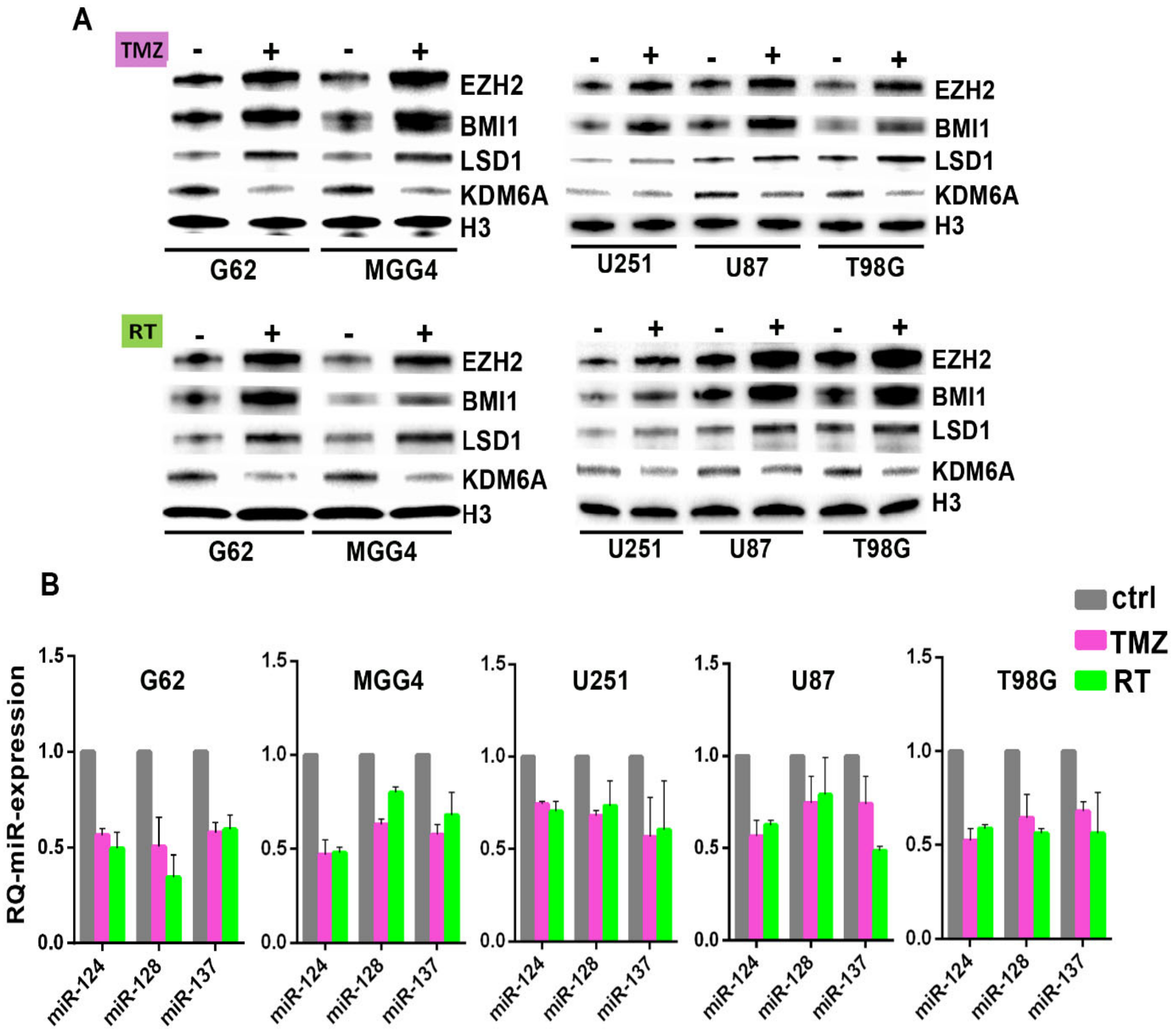

Supplementary Figure 5: Response of GBM cells to temozolomide and radiation.
A: Western blot analysis of EZH2, BMI1 and LSD1 in multiple GBMs (cell lines and GSCs), 24 hours after treatment with TMZ (15 µM for G62, U251, U87, and 100 µM for MGG4 and T98) or with ionizing radiation (2 Gy). **B:** Relative quantification of microRNA expression by RT PCR in the same cells and same conditions as in A. Mean ± SD from two separate experiments.

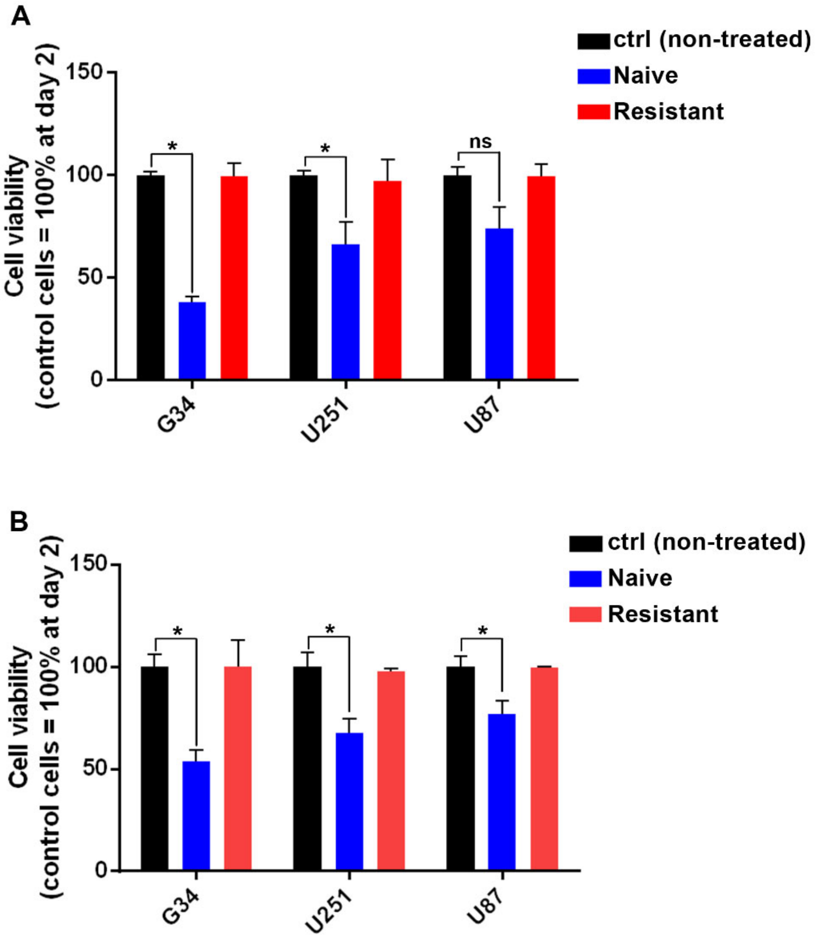

Supplementary Figure 6: Acquired resistance to Temozolomide and irradiation re-challenge after chronic exposure. Relative measurement of metabolic activity by ADP-Glo Kinase assay protocol 48 hours after treatment with 15 µM TMZ (panel A) or 2Gy irradiation (panel B). Blue bars represent cells that were naïve for prior treatment, while red bars represent cells which were previously treated and made resistant using the protocols described in the material and methods section and schematized in the cartoon in Figure 2D and 2E. Proliferation was normalized to untreated cells (black bars). Each experimental setting was performed in triplicates. *=p<0.05 by two tailed Student's t-test.

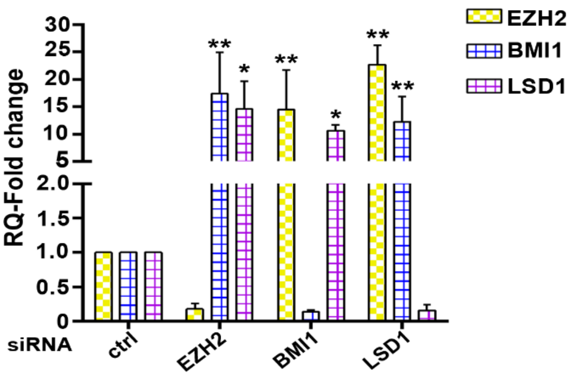

Supplementary Figure 7: Transcriptional activation of correlated chromatin modifiers upon single knock down of EZH2, BMI1 or LSD1. RT-qPCR expression analysis from glioblastoma G34 cells after siRNA-mediated single knock down of EZH2, BMI1 and LSD1. Results are from triplicate experiments. *=p<0.05, **=p<0.01 by Student's t-test, 2 tails.

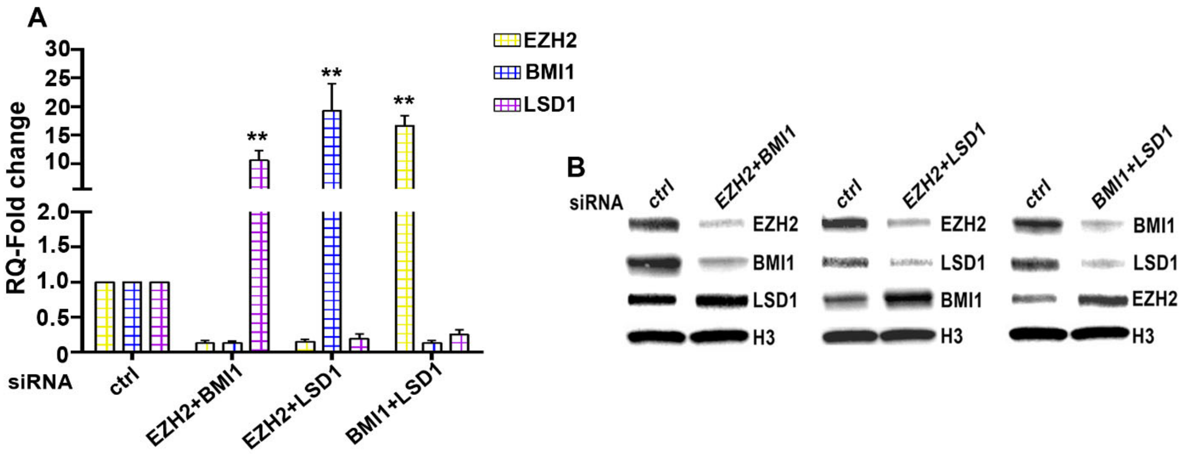

Supplementary Figure 8: Effect of double knock down. A: RT-qPCR showing expression of EZH2, BMI1, and LSD1 after different combinations of double knock down in G34 cells. **B:** Corresponding Western blot from protein lysate obtained from the same cells in panel A. Results are from triplicate experiments. **=p<0.01 by two tailed Student's t-test.

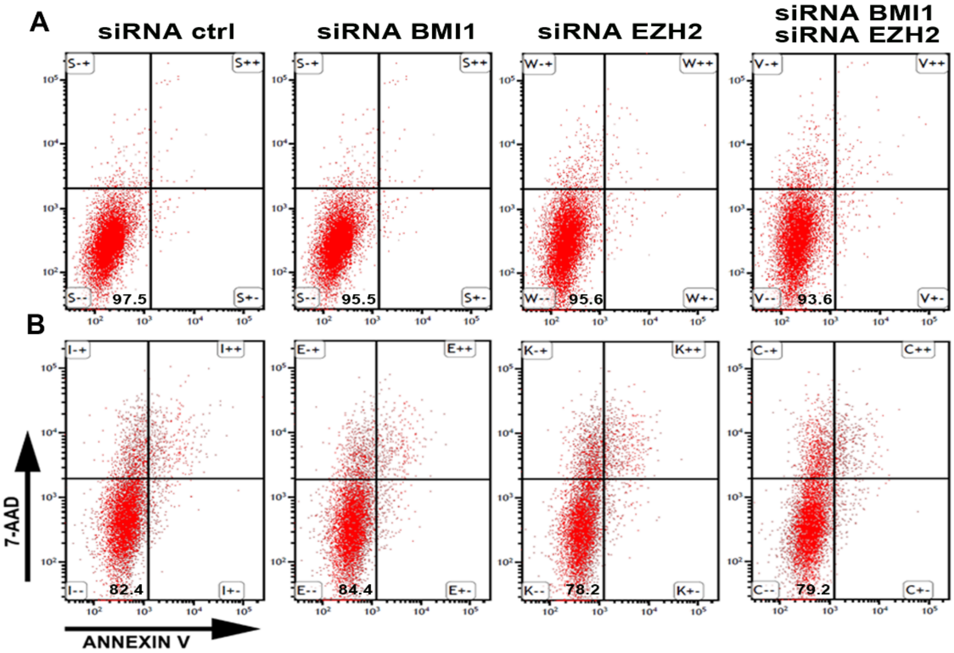

Supplementary Figure 9: Lack of direct cell toxicity by isolated epigenetic modulation in glioblastoma cells. Evaluation of viable U251 cells 72 hours after siRNA-mediated knock down of specified proteins or their combination. **A:** Untreated control for the TMZ treatment shown in Figure 3. **B:** Untreated control for treatment with ionizing radiation in Figure 3. Percentage of viable cells for each group is reported in each quadrant.

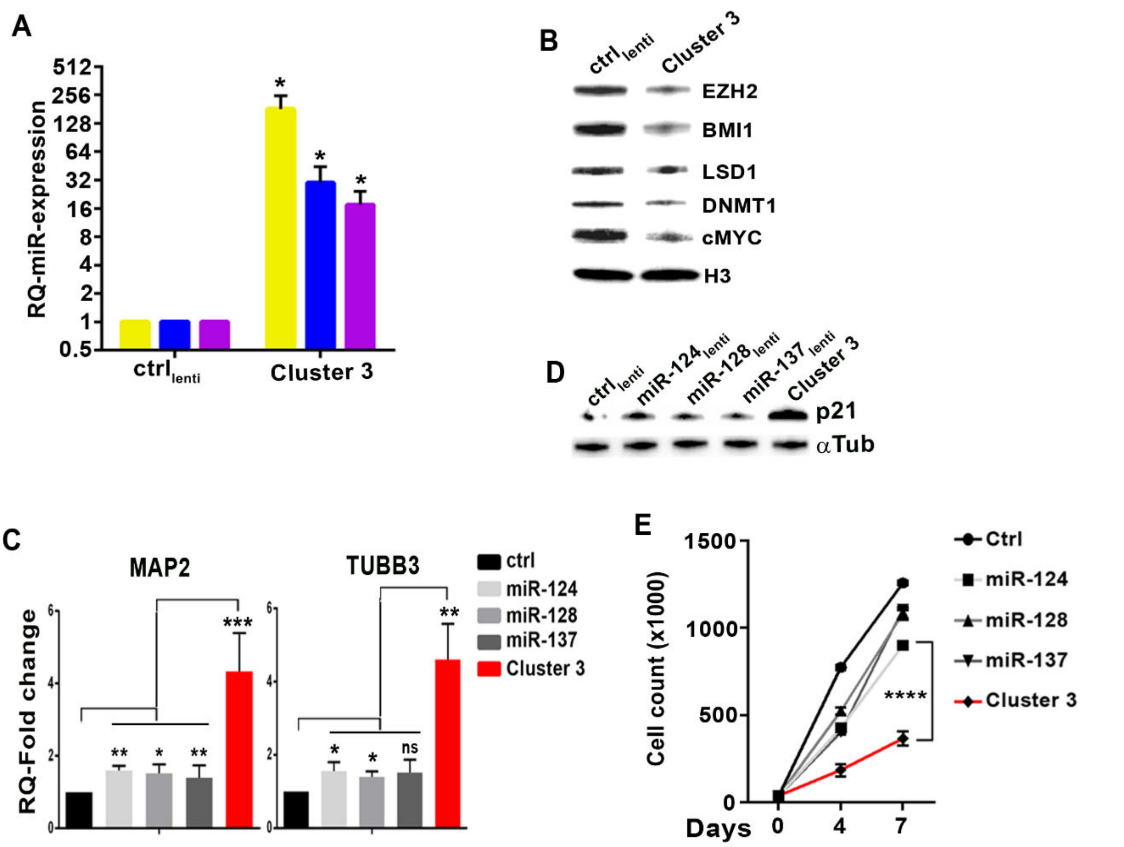

Supplementary Figure 10: Molecular and Biological effect of Cluster 3 in G62 GSC. **A:** Relative quantification of microRNA expression by Real time PCR in G62 GSC transduced with Cluster 3 transgene or negative control (GFP-only transgene). Reported mean ± SD form three independent experiments. **B:** Representative Western blot from cells in A. **C:** Relative quantification of MAP2 and TUBB3 (β3-tubulin) gene expression by Real Time PCR after different microRNA overexpression. Means ± SD of 3 independent experiments. **D:** p21 protein level by Western blot of G62 cells expressing either single miRs or Cluster 3 transgene. **E:** Cell count per well of G62 GSC expressing different microRNAs at 4 and 7 days timepoints. Reported is the mean ± SD from 3 independent experiments. For the sake of clarity, statistical value is reported only for the day 7 endpoint, and only comparing miR-124 as the most effective of the single microRNAs vs Cluster 3). * =p<0.05; ** =p<0.01, ***=p<0.001; ****=p<0.0001 (Student's t-test, 2 tails).

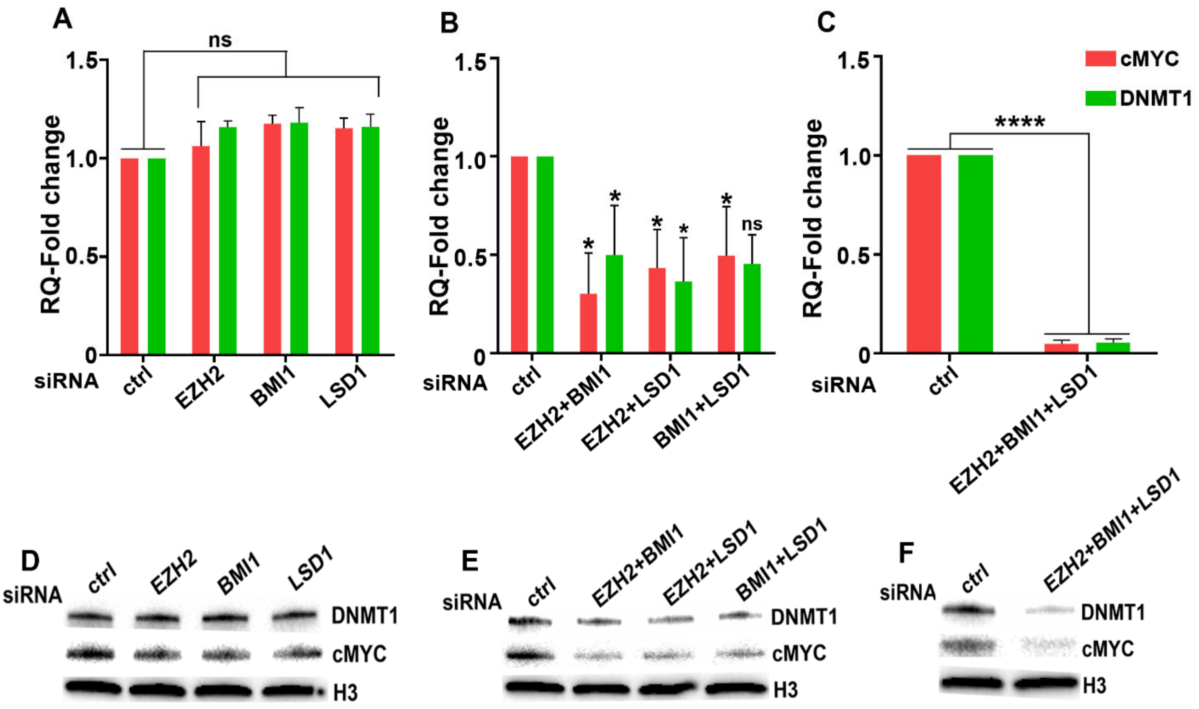

Supplementary Figure 11: DNMT1 and MYC expression incrementally declines upon progressive knockdown of EZH2, BMI1 and LSD1 function. RT-qPCR expression analysis of MYC and DNMT1 genes upon single (**A**), double (**B**) or triple knock down (**C**) of EZH2, BMI1 and LSD1 by siRNA transfection in G34 glioblastoma cells. Corresponding Western blots showing MYC and DNMT1 protein levels are presented in panels **D-F.** Represented data are means from triplicate experiments. *=p<0.05; ****=p<0.0001 by two tailed Student's t-test. ns= not significant.

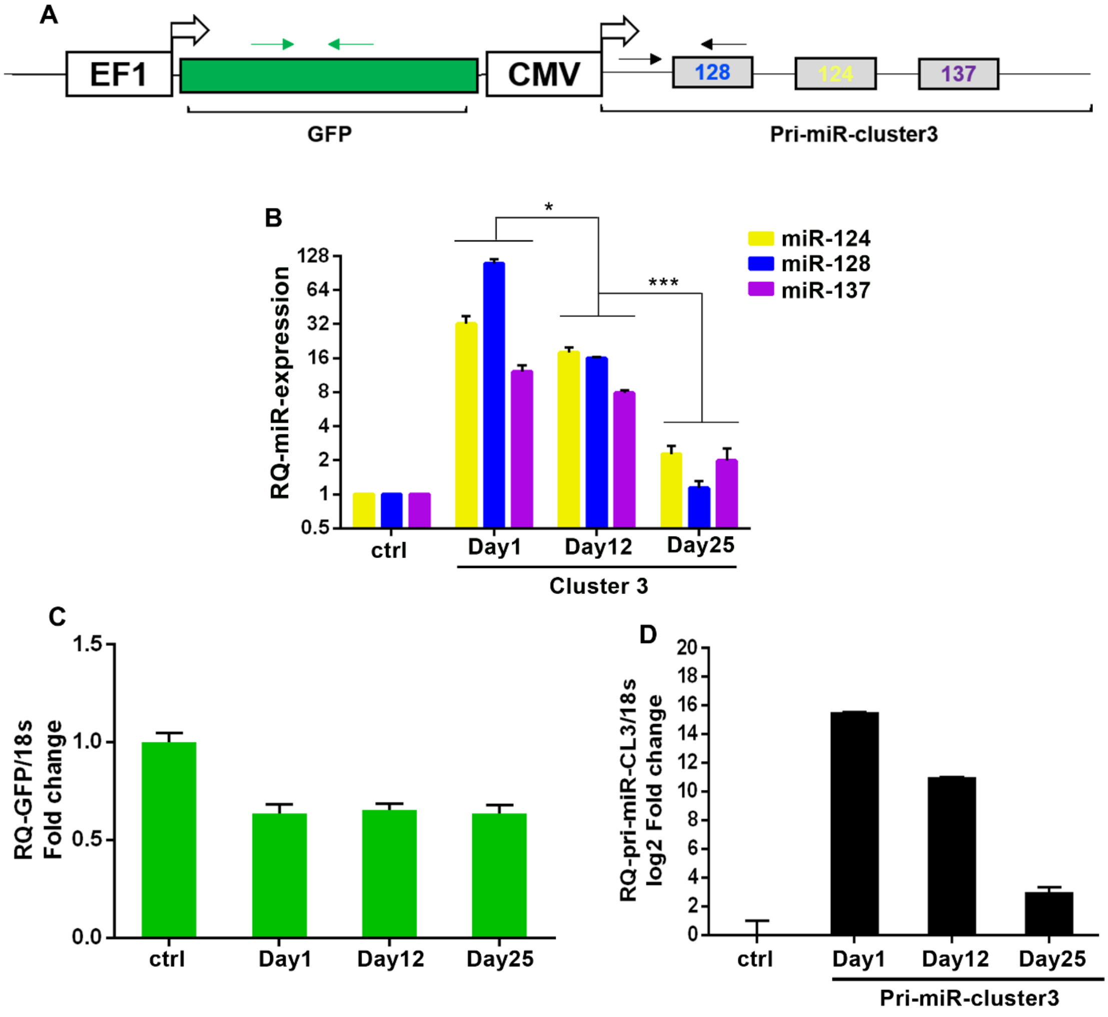

Supplementary Figure 12. Progressive loss of transgene expression *in vivo*. A: Cartoon schematizing the genomic configuration of the lentiviral vector used to overexpress microRNAs in this study. The GFP transgene is downstream to an EF1-responsive element, while the Cluster 3 primary transcript is downstream of the CMV promoter. Colored arrows represent primer sequences used for the RT-qPCR. **B:** Relative quantification of microRNA expression at different timepoints after intracranial implantation. G34 cells previously implanted intracranially in athymic mice were isolated from the brain at time of mouse euthanasia (either at day 12 or at day 25) and the expression of cluster 3 transgene was measured against that of cells expressing negative control and against parental Cluster 3 cells at time of implantation. Reported are Mean ± SD from three independent experiments. **C:** RT-qPCR showing expression of GFP transgene from cells in panel B. **D:** RT-qPCR showing expression of Primary Cluster 3 gene from cells in panel B. Reported are mean + SE from one representative experiment in technical triplicates. *=$p<0.05$; ***=$p<0.001$ (Student's t-test, 2 tails).

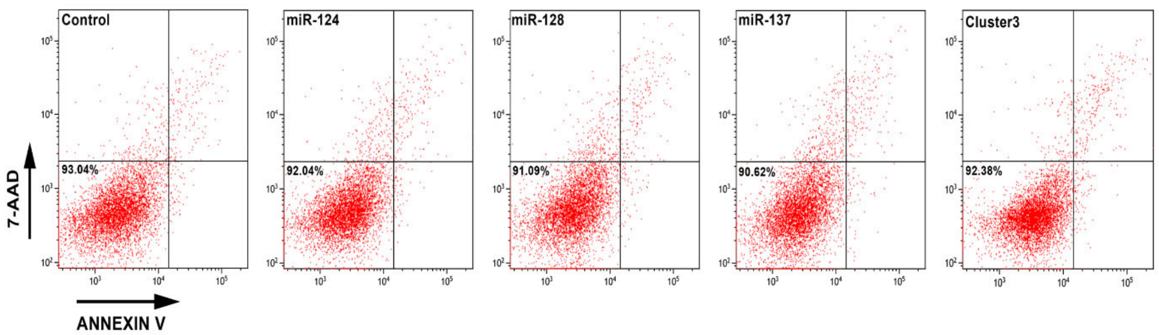

Supplementary Figure 13: Lack of direct GBM cell toxicity by clustered microRNAs. FACS-based evaluation of viable G34 cells 5 days after microRNA overexpression and without any further treatment. For each group, the percentage of viable cells is reported in the left lower quadrant.

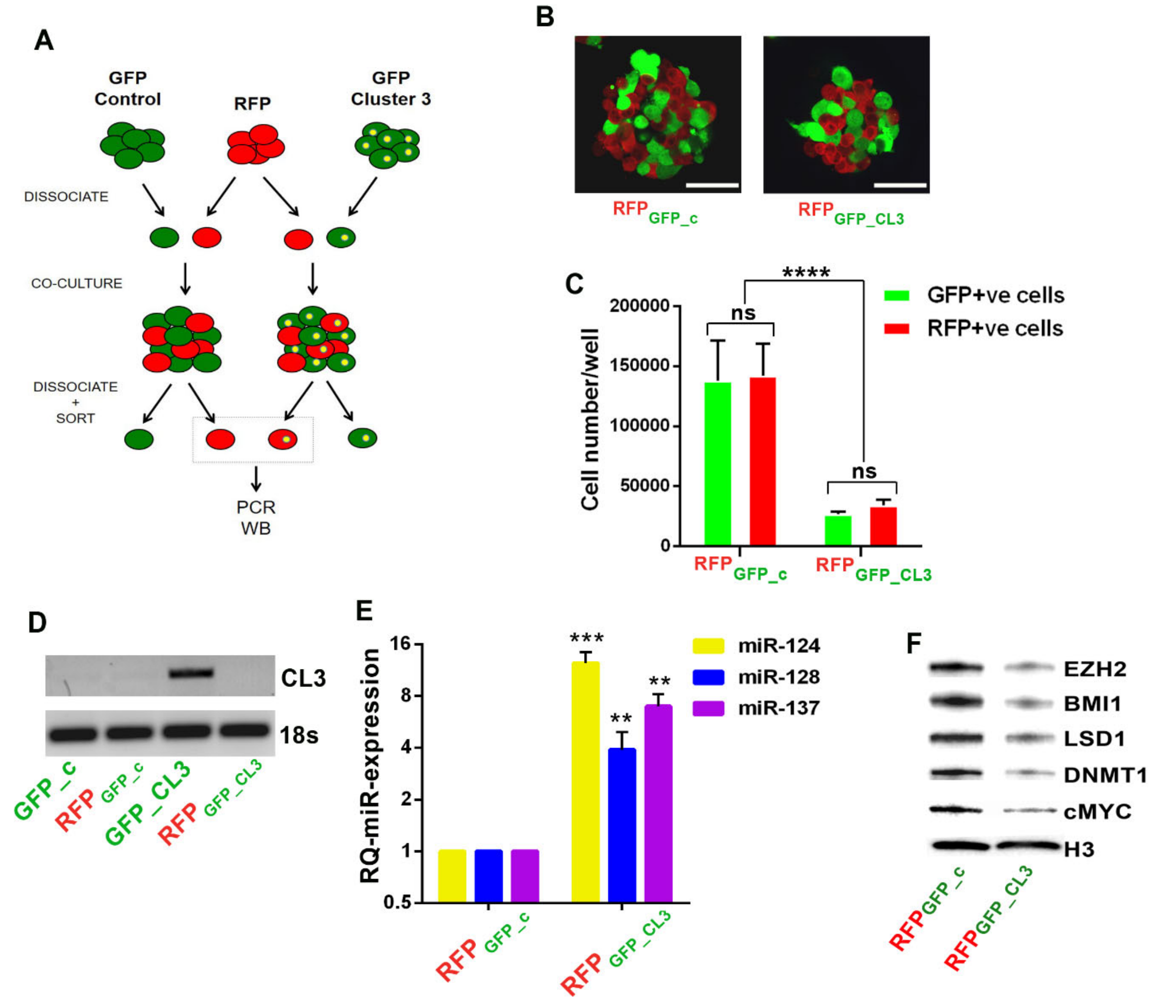

Supplementary Figure 14: *In vitro* mixing of GBM cells shows transfer of transgenic microRNAs. A: Cartoon depicting experimental protocol. Yellow dot represents Cluster 3 transgene. **B**: Confocal microscopy of two exemplificative pictures of chimeric neurospheres composed by a combination of RFP and GFP positive cells after 5 days of co-culture. Scale bars: 50 µm. **C:** Cell count of GFP and RFP-positive cells from spheres in B after dissociation and FACS. Reported are means ± SD of three independent experiments. **D:** Purity of sorted population is confirmed by Cluster 3 transgene amplification by PCR. **E:** Fold change expression of microRNAs in sorted RFP cells. Reported are mean values ± SD from 3 separate experiments. **=p<0.01; ***=p<0.001; ****=p<0.0001 (Student's t-test, 2 tails) **F:** Western blot representing the differential protein levels in sorted RFP cells after co culture with GFP-positive cells.

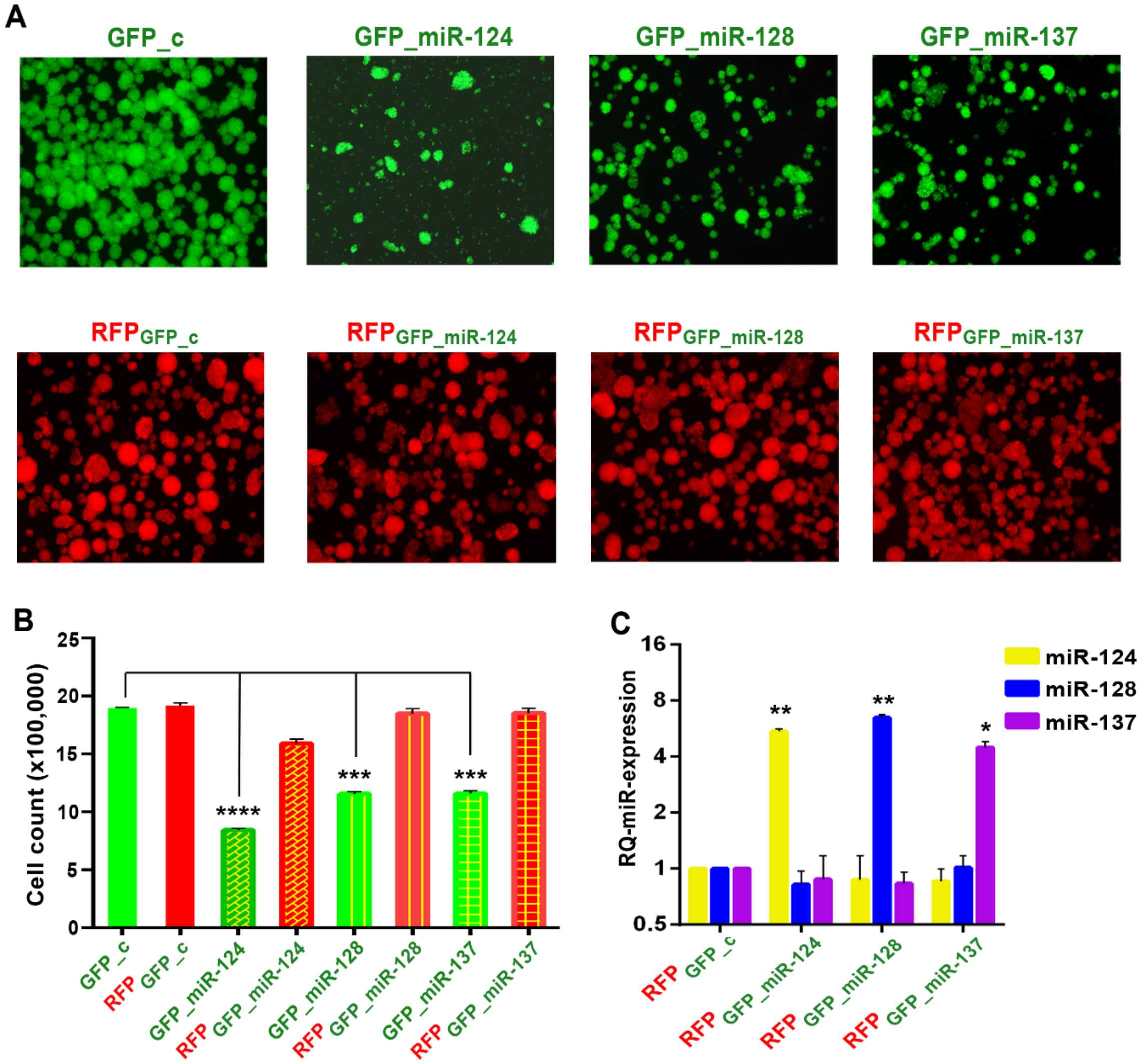

Supplementary Figure 15: Negligible bystander effect by single microRNA transfer in GSC co-culture. A: Representative fluorescent microscope pictures showing cell density in G34 cultures stably expressing single microRNAs vs negative control (upper row, GFP), and RFP-positive cells co-cultured for 5 days with the corresponding GFP-positive cells (lower row). **B**: Cell count of cells in A (three independent experiments). C: Real Time qPCR showing the level of microRNA upregulation in RFP-positive cells in A. *=p<0.05; **=p<0.01; **=p<0.001; ****=p<0.0001 (Student's t-test, 2 tails).

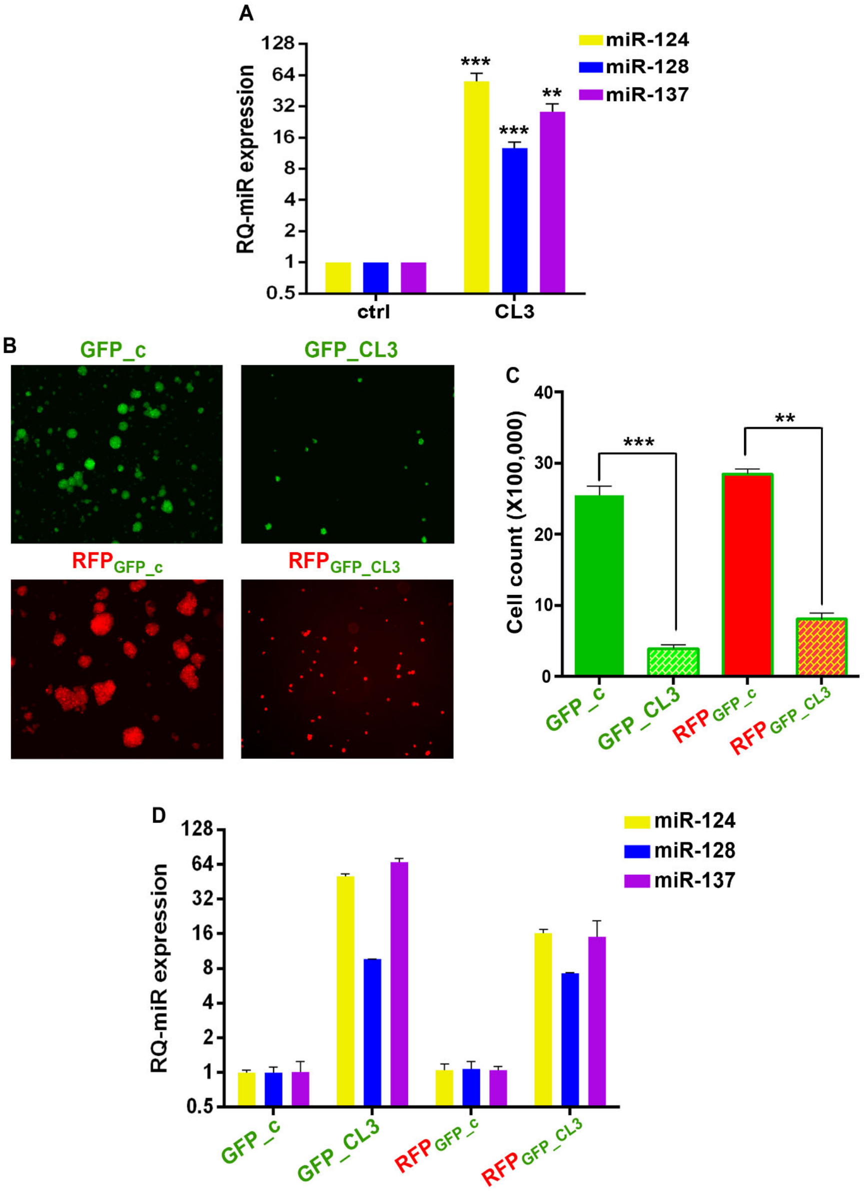

Supplementary Figure 16: microRNA upregulation in bystander cells is independent from lentivirus infection. Transwell assay performed with G34 cells transfected with DNA plasmids encoding either scrambled control or Cluster 3 transgenes but without the use of lentiviruses. **A:** RT-qPCR showing that transfected cells are able to produce mature microRNAs from the cluster 3 transgene. **B:** In a transwell assay, RFP-positive G34 cells growing together but separated from Cluster 3-transfected cells display delayed growth, quantified in panel **C**. **D:** RT-qPCR from cells in panel B, showing increased levels of mature miR-124, miR-128, and miR-137 in the RFP-positive cells growing in proximity of Cluster 3-transfected GFP cells. **=p<0.01; ***=p<0.001 by two tailed Student's t-test in three independent experiments.

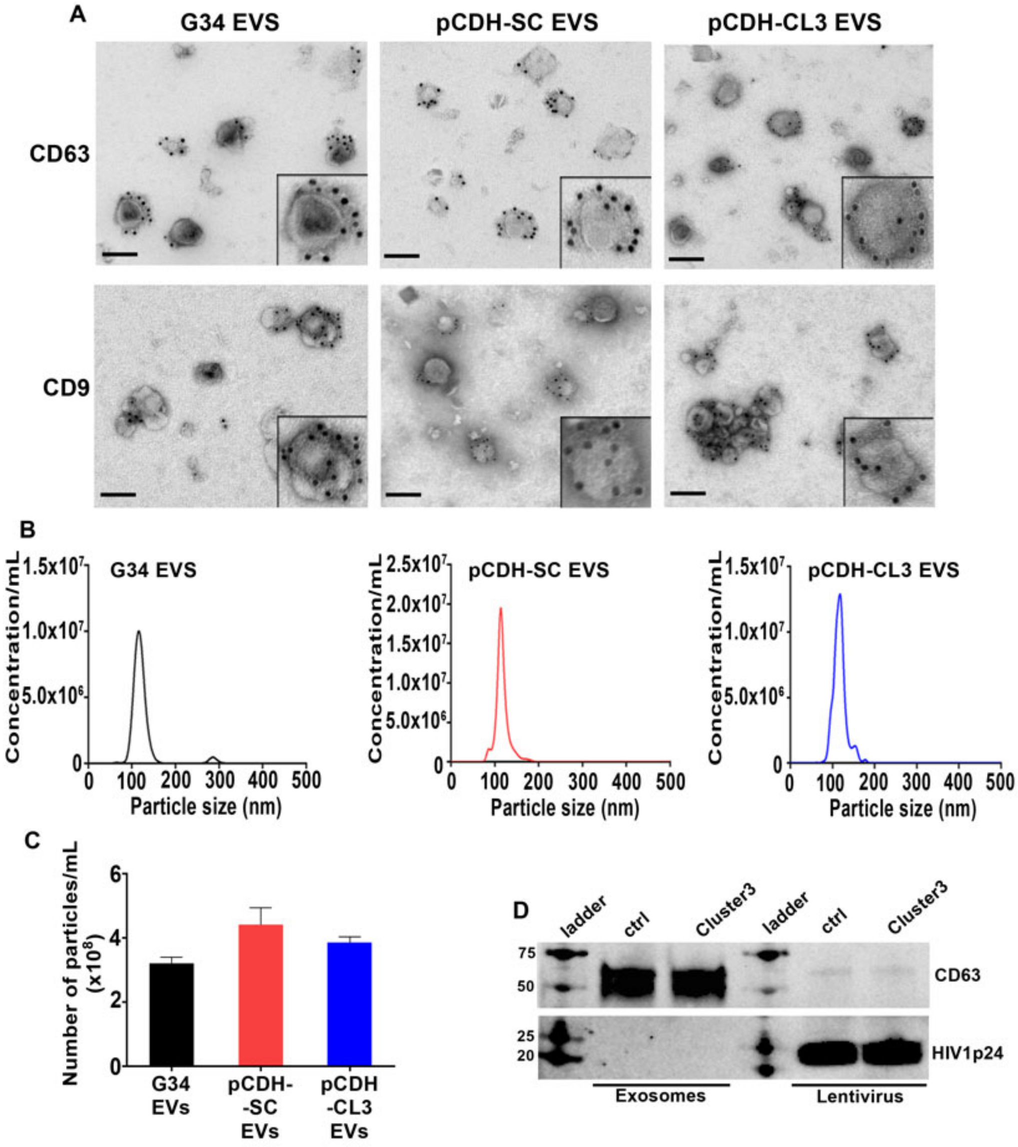

Supplementary Figure 17: Characterization of extracellular vesicles.
A: Transmission Electron Microscopy images of representative EV preparations from either parental non-transduced, scrambled control and cluster 3-infected G34 cells. Immunolabeling for tetraspanins CD63 and CD9 was performed to show the enrichment of EVs markers. Bar=100 nm. Each picture has a 4x magnification box detailing immunolabeling. **B:** Nanosight analysis of the three EV preparations shown in panel A, demonstrating similar vesicle size distribution and homogeneity. **C:** Concentration of EVs for each preparation. **D:** Western blot of EVs (10 μg/lane) obtained from scrambled control (ctrl) or Cluster 3-infected cells, in comparison to 1 μg/lane of the corresponding lentivirus preparations used to infect the cells 2 weeks prior to EV collection.

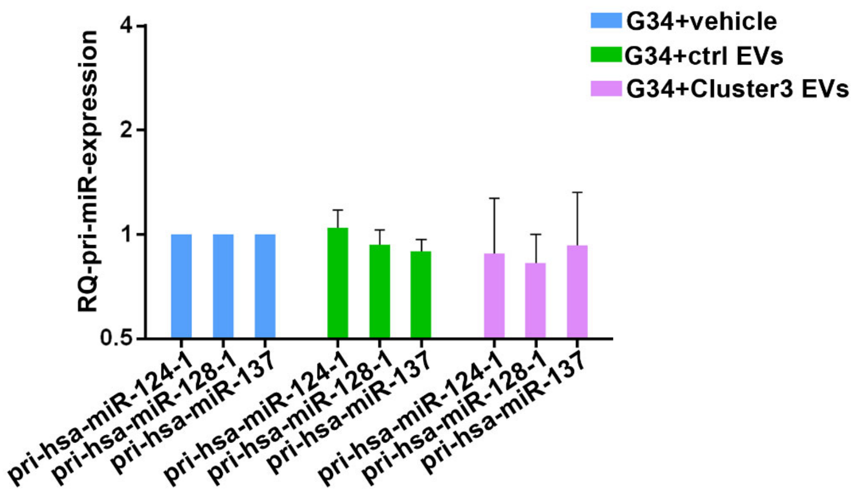

Supplementary Figure 18: EV administration does not induce expression of endogenous microRNA genes. RT-qPCR of primary microRNAs expression comparing the non-treated G34 cells (G34+vehicle, blue bars) against those incubated with EVs derived either from negative control cells (green bars) or from Cluster 3 cells (pink bars). Reported data are mean from duplicate experiments ±SD.

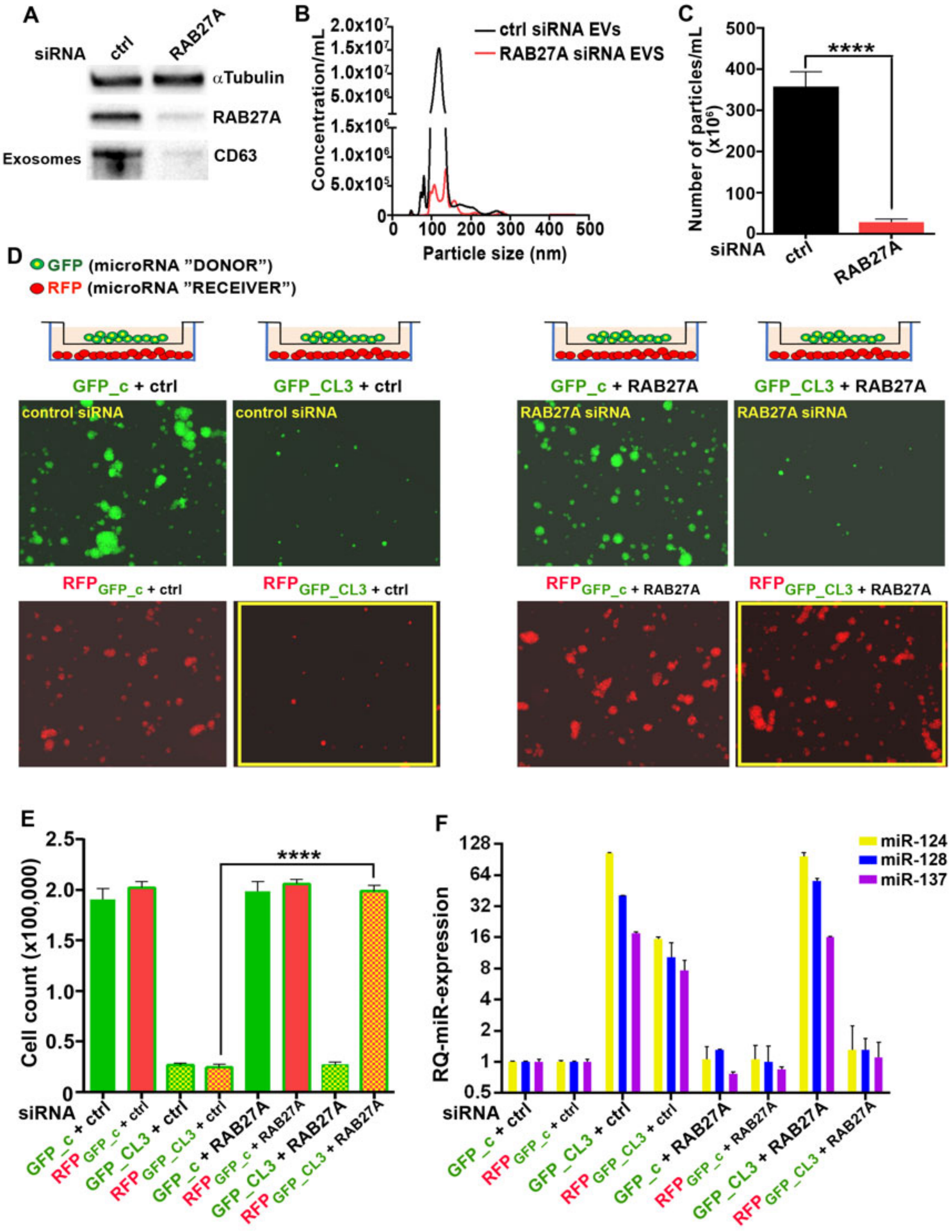

Supplementary Figure 19: Loss of microRNA transfer upon downregulation of the EVs secretory pathway. A: Western blot from G34 cells after treatment with RAB27A siRNA or negative control siRNA, showing strong RAB27A knockdown and resultant decrease in CD63 abundance in ultracentrifugation preparations. **B:** Nanosight analysis of size distribution of EVs obtained from cells in A. **C:** Nanosight quantification of EVs concentration of the two preparations. **D:** Transwell cultures of GFP-positive G34 cells

stably expressing scrambled microRNA transgene (c) or Cluster 3 transgene (CL3), labelled in green, in the presence of control siRNA (ctrl) or RAB27A siRNA (RAB27A), labelled in black. Receiving RFP cells were not treated with any siRNAs. Each column represents a separate transwell assay. The 2 yellow frames underline the two samples whose statistical difference is reported in panel E. **E:** Cell counts of samples in D (mean ±SD). **F:** RT-qPCR measuring level of mature microRNAs in the samples described in D. Experiments were performed in triplicates, except for microRNA qPCR (F), which was performed once and Mean + SE of technical triplicates is shown. ***=p<0.001; ****=p<0.0001 by two tailed Student's t-test.

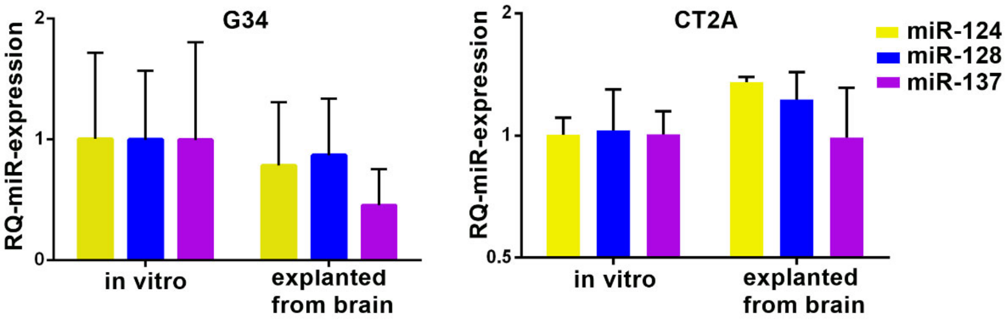

Supplementary Figure 20: Absent transfer of microRNAs from mouse brain to implanted GBM cells. Relative quantification of microRNA expression in human G34 GBM cells **(A)** and mouse CT2A GBM cells **(B)** after they were recovered from an intracranial tumor graft in athymic mice (12 days), in comparison to parental cells ("in vitro") before implantation. Reported values are means ± SD of three different experiments for G34 and one experiment for CT2A.

Figure 2A

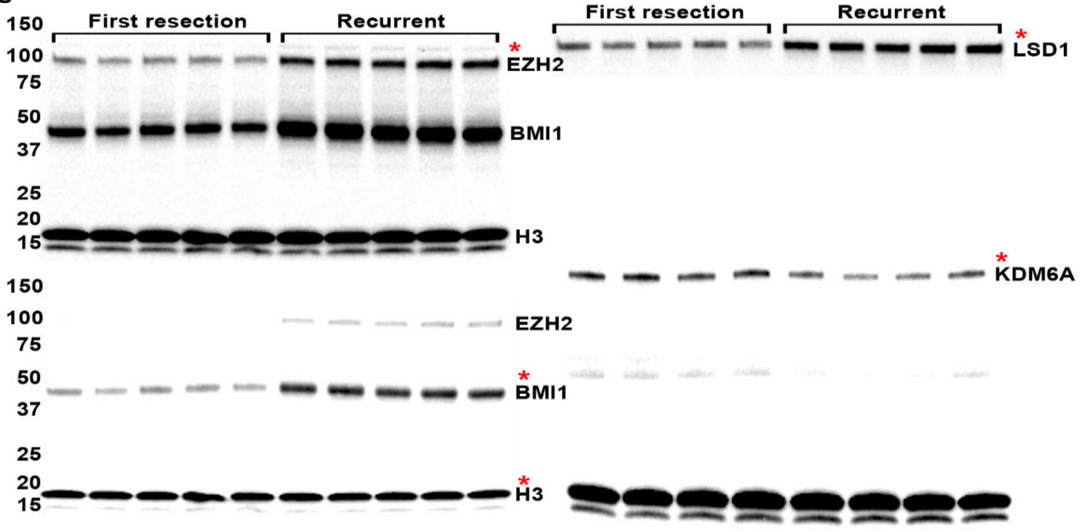

Figure 2B

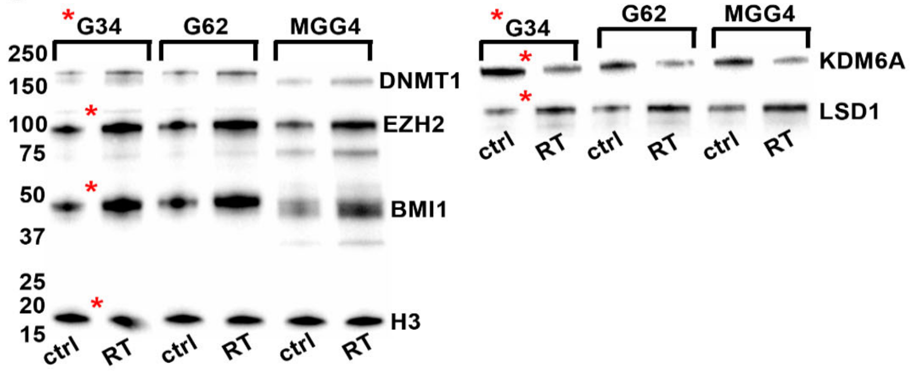

Figure 2J

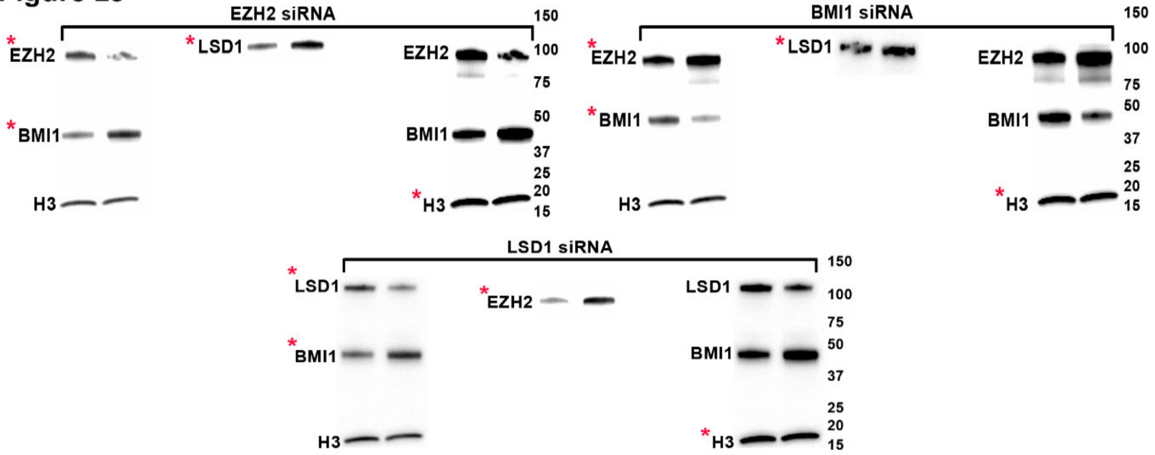

Supplementary Figure 21: Asterisks shows the representative blot image in the respective figures.

Figure 3C

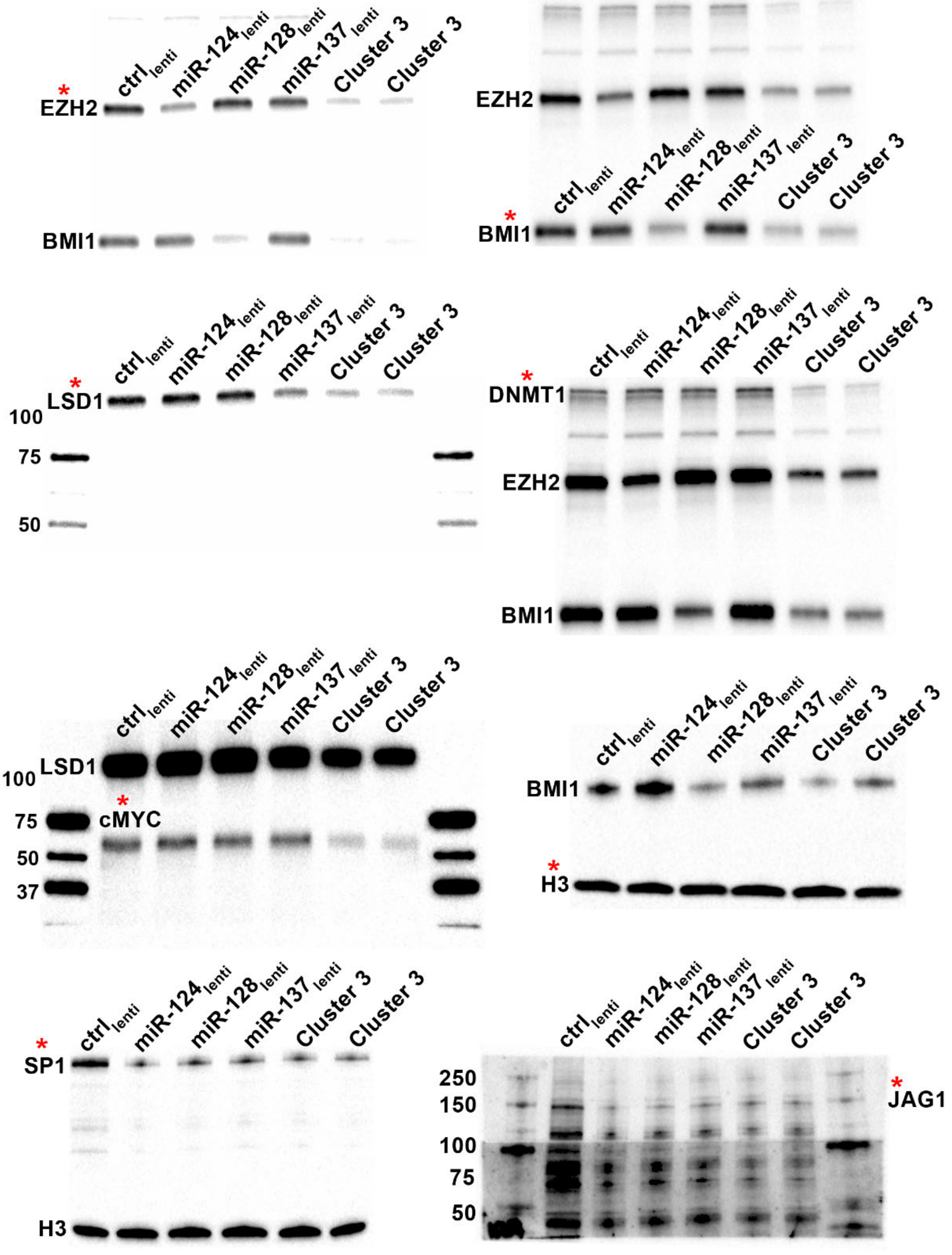

Supplementary Figure 22: Asterisks shows the representative blot image in the respective figures.

Figure 4C

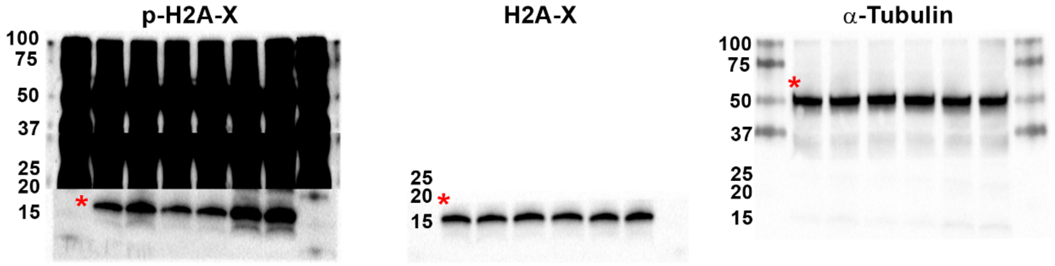

Figure 4D

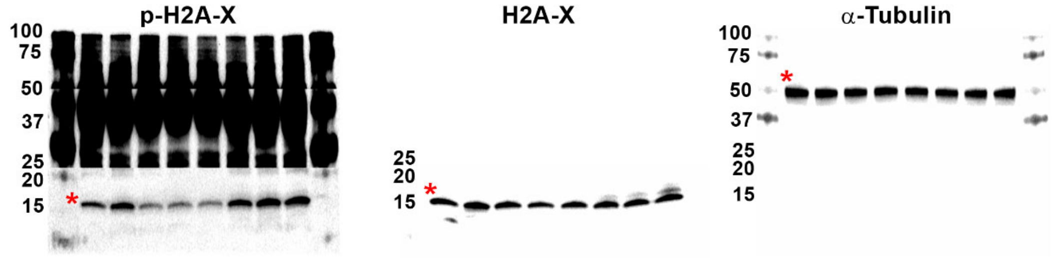

Supplementary Figure 23: Asterisks shows the representative blot image in the respective figures.

Figure 6B

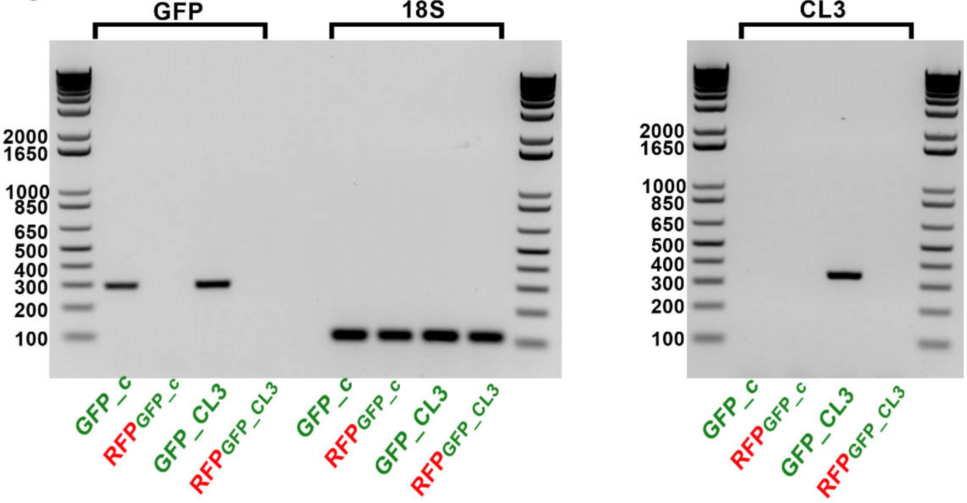

Figure 6D

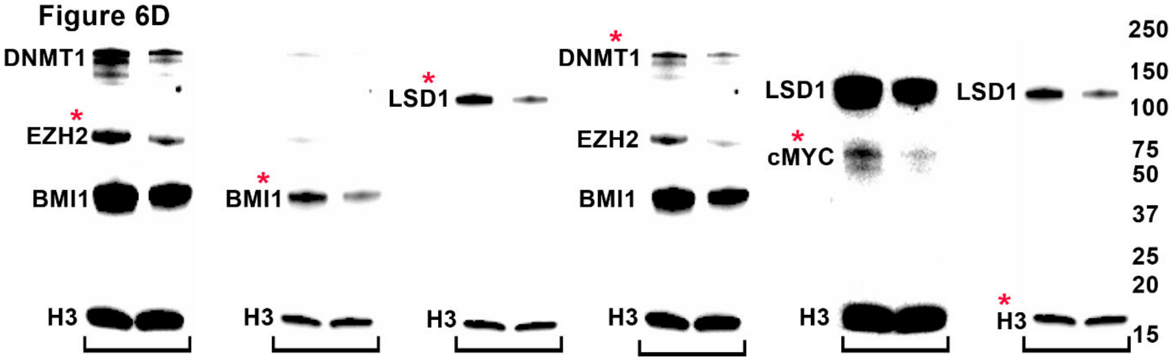

Supplementary Figure 24: Asterisks shows the representative blot image in the respective figures.

Figure S17D

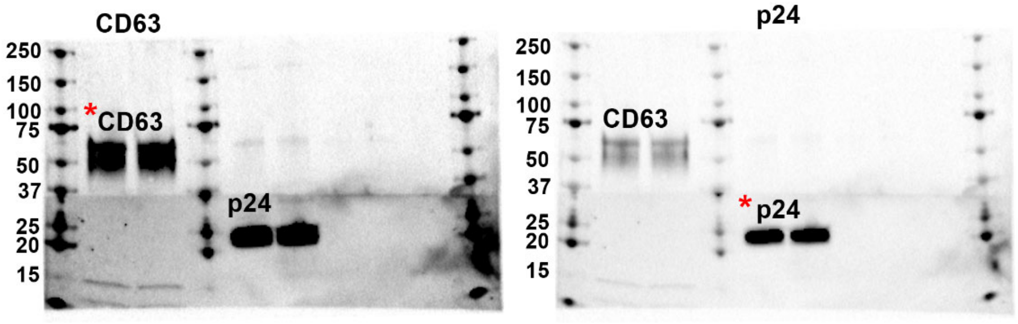

Figure S19A

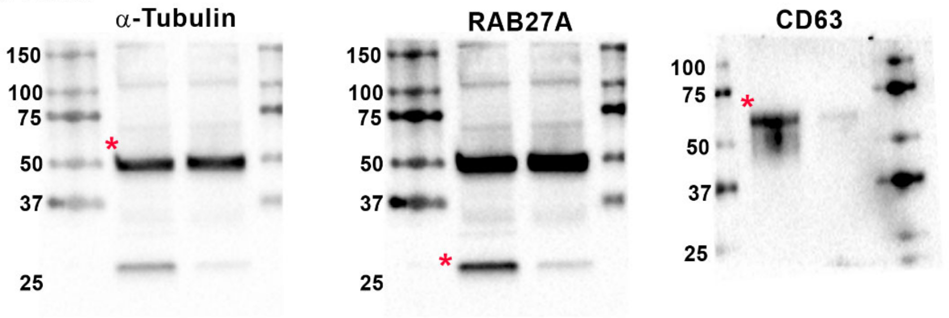

Supplementary Figure 25: Asterisks shows the representative blot image in the respective figures.

miR-124 targets

Rank	GO category	Biological process	pValue	Bonferroni
1	GO:0048666	neuron development	2.06E-12	1.40E-08
2	GO:0030030	cell projection organization	2.61E-12	1.77E-08
3	GO:0031175	neuron projection development	2.91E-12	1.97E-08
4	GO:0048699	generation of neurons	4.30E-12	2.91E-08
5	GO:0030182	neuron differentiation	5.93E-12	4.02E-08
6	GO:0022008	neurogenesis	9.97E-12	6.76E-08
7	GO:0006357	regulation of transcription from RNA polymerase II promoter	1.32E-11	8.96E-08
8	GO:0045944	positive regulation of transcription from RNA polymerase II promoter	6.31E-11	4.28E-07
9	GO:0000902	cell morphogenesis	1.01E-09	6.85E-06
10	GO:0000902	cellular component morphogenesis	2.93E-09	1.98E-05

miR-128 targets

Rank	GO category	Biological process	pValue	Bonferroni
1	GO:0072359	circulatory system development	6.85E-20	4.71E-16
2	GO:0072358	cardiovascular system development	6.85E-20	4.71E-16
3	GO:0009790	embryo development	1.16E-19	7.96E-16
4	GO:0006357	regulation of transcription from RNA polymerase II promoter	8.03E-18	5.52E-14
5	GO:0048699	generation of neurons	1.00E-17	6.88E-14
6	GO:0043009	chordate embryonic development	3.61E-17	2.48E-13
7	GO:0009792	embryo development ending in birth or egg hatching	7.99E-17	5.49E-13
8	GO:0030182	neuron differentiation	9.14E-17	6.28E-13
9	GO:0022008	neurogenesis	1.05E-15	7.21E-12
10	GO:0007417	central nervous system development	1.84E-15	1.27E-11

miR-137 targets

Rank	GO category	Biological process	pValue	Bonferroni
1	GO:0006357	regulation of transcription from RNA polymerase II promoter	2.99E-15	2.06E-11
2	GO:0000122	negative regulation of transcription from RNA polymerase II promoter	4.94E-14	3.41E-10
3	GO:0048699	generation of neurons	6.42E-14	4.43E-10
4	GO:0022008	neurogenesis	7.59E-14	5.24E-10
5	GO:0061061	muscle structure development	1.67E-12	1.15E-08
6	GO:2000113	negative regulation of cellular macromolecule biosynthetic process	7.27E-12	5.02E-08
7	GO:0051172	negative regulation of nitrogen compound metabolic process	1.79E-11	1.24E-07
8	GO:0030182	neuron differentiation	3.23E-11	2.23E-07
9	GO:0060537	muscle tissue development	4.09E-11	2.82E-07
10	GO:0031327	negative regulation of cellular biosynthetic process	9.56E-11	6.60E-07

Supplementary Table 1: GO categories of miR targets. Listed are the 10 most significantly enriched biological process regulated by the predicted targets of each microRNA. Each category is annotated by its own identifier.

**Hsa-miR-124**	GAATTCGCCCTTGAACAAAGAGCCTTTGGAAGACGTCGCTGTTATCTCATTGTCTGTGTGATTGGGGGAGCT<br>GCGGCGGGGAGGATGCTGTGGTCCCTTCCTCCGGCGTTCCCCACCCCCATCCCTCTCCCCGCTGTCAGTGCG<br>CACGCACACGCGCCGCTTTTTATTTCTTTTTCCTGGTTTTCTTATTCCATCTTCTACCCACCCCTCTTCCTT<br>TCTTTCACCTTTCCTTCCTTCCTTCCTCCTTTCCTTCCTCAGGAGAAAGGCCTCTCTCTCCGTGTTCACAGC<br>GGACCTTGATTTAAATGTCCATACAATTAAGGCACGCGGTGAATGCCAAGAATGGGGCTGGCTGAGCACCGT<br>GGGTCGGCGAGGGCCCGCCAAGGAAGGAGCGACCGACCGAGCCAGGCGCCCTCCGCAGACCTCCGCGCAGCG<br>GCCGC
**Hsa-miR-128**	GAATTCATGTCAGACTAGCATCCCTGGCATGCATGTTAAACAGTCTCCATAAATTATTTTTTGATCCTTCTT<br>CTGTTAAAGCAGAAAGTCAACCATGTCCGTACCTTTCTAGTTCATACCTTCTTTTAATTTTTTTTTTCTTTT<br>CAATTTGAAGAGAGTGCTTCCTCTGTTCTTAAGGCTAGGGAACCAAATTAGGTTGTTTCAATATCGTGCTAA<br>AAGATACTGCCTTTAGAAGAAGGCTATTGACAATCCAGCGTGTCTCGGTGGAACTCTGACTCCATGGTTCAC<br>TTTCATGATGGCCACATGCCTCCTGCCCAGAGCCCGGCAGCCACTGTGCAGTGGGAAGGGGGGCCGATACAC<br>TGTACGAGAGTGAGTAGCAGGTCTCACAGTGAACCGGTCTCTTTCCCTACTGTGTCACACTCCTAATGGAAT<br>GCCGTTATCCAAAGAGCAGCACGAACCCGACAGGGCTGAGTGGCTTGTGCTAGGGAGAGGTTTGTGTCATTC<br>CTGCTGACCAAACTGCAGGAAAAACTGCTAATTGTCATGCTGAAGACTGCCTGACGGGGAGACTCTGCCTTC<br>TGTAAGTAGGTCATGTAAAGAGCACGTGCTCCTTGCTGCTACTCATAGATGCCTGCTCCGTGATCTGATTTC<br>TGCACTGAATCTATGTTATGCATATGGAATGTATACAGATACATGTATGTAATATATACACAATATGTACCA<br>TTTTTATAACCTATGAGAAAAGTGCAGTTCTTGCCAAGGATTAGCACCAATCACAGACATCCTAGCGGCCGC
**Hsa-miR-137**	TCTAGAGCCACATCTTCCCTCCTCACTGGAAAGACAGCACTCTTCTGTGTTAAGTATTTGATTTTGTGATTT<br>GTCTTTCAGAATTGGAAATAGAGCGGCCATTTGGATTTGGGCAGGAAGCAGCCGAGCACAGCTTTGGATCCT<br>TCTTTAGGGAAATCGAGTTATGGATTTATGGTCCCGGTCAAGCTCAGCCCATCCCCAGGCAGGGGCGGGCTC<br>AGCGAGCAGCAAGAGTTCTGGTGGCGGCGGCGGCGGCAGTAGCAGCGGCAGCGGTAGCAGCGGCAGCGGTAG<br>CAGCGGCAGCGGCAGCTTGGTCCTCTGACTCTCTTCGGTGACGGGTATTCTTGGGTGGATAATACGGATTAC<br>GTTGTTATTGCTTAAGAATACGCGTAGTCGAGGAGAGTACCAGCGGCAGGGGGGCAGCGGCCGCCCTCCCCA<br>GCCCACCAGCTGGCCACTAAACGCCCGTGGTTGCCAAGGTAGCACTTTCTTGTTCTTTTCATTTCCTCGGGT<br>GTTTTCGCACTGGTTCCACCGGAAAGGCTGTGCGCTGCGCCTCTGGTGACCAGGACTGGAAAATGGGTTCGC<br>GCAGGGGGCGGGGGGCGAGGTGAAGGGGATGGGCGGGGAGCGGAAGGGGGTCCGTTTCCCCGAGCCTGCGGG<br>GCGGTTCGGCGTTCCCAGTCCGCTCCTCTCCTCTCCACGGTGGGGTGCGGCTGGAGGGAGGCGGCCGC
**Hsa-Cluster3**	TCTAGACTTTTCAATTTGAAGAGAGTGCTTCCTCTGTTCTTAAGGCTAGGGAACCAAATTAGGTTGTTTCAA<br>TATCGTGCTAAAAGATACTGCCTTTAGAAGAAGGCTATTGACAATCCAGCGTGTCTCGGTGGAACTCTGACT<br>CCATGGTTCACTTTCATGATGGCCACATGCCTCCTGCCCAGAGCCCGGCAGCCAGTCCAGTGGGAAGGGGGG<br>CCGATACACTGTACGAGAGTGAGTAGCAGGTCTCACAGTGAACCGGTCTCTTTCCCTACTGGACAGCTGCCT<br>CGGGAAGCCAAGTTGGGCTTTAAAGTGCAGGGCCTGCTGATGTTGAGTGCTTTTTGTTGGCCTCTCTCTCCG<br>TGTTCACAGCGGACCTTGATTTAAATGTCCATACAATTAAGGCACGCGGTGAATGCCAAGAATGGGGCTGGC<br>ATAAGAAGTTATGTATTCATCCAATAATTCAAGCCAAGCAAGTATATAGGTGTTTTAATAGTTTTTGTTTGC<br>ACTGACTCTCTTCGGTGACGGGTATTCTTGGGTGGATAATACGGATTACGTTGTTATTGCTTAAGAATACGC<br>GTAGTCGAGGAGAGTACCAGTGCACACTCCTAATGGAATGCCGTTATCCAAAGAGCAGCACGAACCCGACAG<br>GGCTGAGTGGCTTGTGCTAGGGAGAGGTTTGTGTCATTCCTGCTGACCAAACTGCAGGAAAAACTGCTAATT<br>GTCATGCTGAAGACTGCCTGACGGGGAGACTCTGCCTTCTGTAAGTAGGTCATGTAAAGAGCACGTGCTCCT<br>TGCTGCTGCGGCCGC
**Hsa-Scrambled Cluster3**	TCTAGACTTTTCAATTTGAAGAGAGTGCTTCCTCTGTTCTTAAGGCTAGGGAACCAAATTAGGTTGTTTCAA<br>TATCGTGCTAAAAGATACTGCCTTTAGAAGAAGGCTATTGACAATCCAGCGTGTCTCGGTGGAACTCTGACT<br>CCATGGTTCACTTTCATGATGGCCACATGCCTCCTGCCCAGAGCCCGGCAGCCAGTCCAGTGGGAAGGGGGG<br>CCGATACACTGTACGAGAGTGAGTAGCAGGTCATCGTCATTCGATTCACTGGCCCCTACTGGACAGCTGCCT<br>CGGGAAGCCAAGTTGGGCTTTAAAGTGCAGGGCCTGCTGATGTTGAGTGCTTTTTGTTGGCCTCTCTCTCCG<br>TGTTCACAGCGGACCTTGATTTAAATGTCCATACAATGCGCAAGACGCAATGTCAGAGTGAATGGGGCTGGC<br>ATAAGAAGTTATGTATTCATCCAATAATTCAAGCCAAGCAAGTATATAGGTGTTTTAATAGTTTTTGTTTGC<br>ACTGACTCTCTTCGGTGACGGGTATTCTTGGGTGGATAATACGGATTACGTTGGCTAGTAACTTCGTATATA<br>AGGTTCGAGGAGAGTACCAGTGCACACTCCTAATGGAATGCCGTTATCCAAAGAGCAGCACGAACCCGACAG<br>GGCTGAGTGGCTTGTGCTAGGGAGAGGTTTGTGTCATTCCTGCTGACCAAACTGCAGGAAAAACTGCTAATT<br>GTCATGCTGAAGACTGCCTGACGGGGAGACTCTGCCTTCTGTAAGTAGGTCATGTAAAGAGCACGTGCTCCT<br>TGCTGCTGCGGCCGC

Supplementary Table 2: Transgenic sequences used for overexpression of microRNAs. Underlined are the flanking sequences used for site specific cloning into expression vector pCDH-CMV-MCS-EF1-copGFP (System Biosciences, cat # CD511b-1)

Target	Sequence	Species specificity
EZH2 forward	5'-CTGATTTTACACGCTTCCGC	Human
EZH2 reverse	5'-GGAACAACGCGAGTCGG	Human
BMI1 forward	5'-TCGTTGTTCGATGCATTTCT	Human
BMI1 reverse	5'-CTTTCATTGTCTTTTCCGCC	Human
KDM1A (LSD1) forward	5'-ACACTGCAGTTGTGGTTGGA	Human
KDM1A (LSD1) reverse	5'-TAAATAACTGTGAACTCGGT	Human
cMYC forward	5'-CACCGAGTCGTAGTCGAGGT	Human
cMYC reverse	5'-TTTCGGGTAGTGGAAAACCA	Human
DNMT1 forward	5'-AGCAAGTCGGACAGTGACAC	Human
DNMT1 reverse	5'-CCCTCTTCCGACTCTTCCTTG	Human
MAP2 forward	5'-TTCGTTGTGTCGTGTTCTCA	Human
MAP2 reverse	5'-AACCGAGGAAGCATTGATTG	Human
TUBB3 forward	5'-TTTCATCTTTGGTCAGAGTG	Human
TUBB3 reverse	5'-AGTCGCAGTTTTCACACTCC	Human
copGFP forward	5'-CGCATGACCAACAAGATGAAG	Pontellina Plumata
copGFP reverse	5'-TGAAGATCACGCTGTCCTCG	Pontellina Plumata
Primary miR cluster transcript forward	5'-CCTCTGTTCTTAAGGCTAGGGAACC	Human
Primary miR cluster transcript reverse	5'-TCACTCTCGTACAGTGTATCG	Human
Cluster 3 Transgene forward	5'-CCCTACTGGACAGCTGCCTC	Human
Cluster 3 Transgene reverse	5'-GAGTGTGCACTGGTACTCTCCTCG	Human
rRNA 18S forward	5’-AACTTTCGATGGTAGTCGCCG	Human/ mouse
rRNA 18S reverse	5’-CCTTGGATGTGGTAGCCGTTT	Human/ mouse

Supplementary Table 3: DNA sequences used for PCR primers

Target (working dilution)	Vendor	Catalog number
EZH2 (IHC) (1:50)	Cell Signaling Technology	D2C9
BMI1 (IHC) (1:200)	Cell Signaling Technology	D20B7
LSD1 (IHC) (1:200)	Cell Signaling Technology	2139S
EZH2 (1:1000)	Cell Signaling Technology	D2C9
BMI1 (1:1000)	Cell Signaling Technology	D42B3
LSD1 (1:1000)	Cell Signaling Technology	C69G12
(UTX) KDM6A (1:1000)	Cell Signaling Technology	D3Q1I
DNMT1 (1:1000)	Cell Signaling Technology	D63A6
cMYC (1:1000)	Cell Signaling Technology	D84C12
SP1 (1:1000)	ABclonal Technology	A3331
JAG1 (1:1000)	ABclonal Technology	A12733
p21 Waf1/Cip1 (1:1000)	Cell Signaling Technology	12D1
Phospho-Histone H2A.X(Ser139) (1:1000)	Cell Signaling Technology	20E3
Histone H2A.X (1:1000)	Cell Signaling Technology	2595S
β3-Tubulin (1:1000)	Cell Signaling Technology	D71G9
GFAP (1:1000)	Cell Signaling Technology	D1F4Q
Nestin (1:1000)	Santa Cruz Biotechnology	SC-23927
α-Tubulin (1:5000)	Sigma Aldrich	T6074
Histone H3 (1:2000)	Cell Signaling Technology	D1H2
CD63 (1:1000)	BD Pharmingen	556019
HIV1 p24 (1:1000)	ThermoFisher Scientific	N29 (05-005)

Supplementary Table 4: Antibodies used for Western blotting and IHC

Supplementary Methods

Immunohistochemistry
Formalin-fixed paraffin embedded (FFPE) tissues specimens (5µm thickness) of GBM and normal brain were procured from the Pathology bio-repository at the Brigham & Women's Hospital. The sections were processed for immunostaining by the Specialized Histopathology and Tissue Micro-Array Core facility at BWH (antibodies used for IHC are listed in supplementary table S4). Sections were imaged using a Nikon eclipse Ti motorized microscope system, Japan (NIS-Elements 4.2).

PCR
For quantification of primary microRNA expression, total RNA was reverse-transcribed using iScript cDNA Synthesis Kit (BioRad) and qPCR was performed using TaqMan Gene Expression Master mix (Applied Biosystems) and TaqMan probes (Life Technologies) of hsa-mir-124-1 (Hs03303077-pri), hsa-mir-128-1 (Hs03303360_pri) and hsa-mir-137 (Hs03303136_pri) using 18S RNA as the internal control.
For semi-quantitative PCR, the primary Cluster 3 transgene and GFP transgene were PCR amplified using 50ng of cDNA and the primers listed in the Supplemental Table S4 using Phusion High-Fidelity PCR Kit (New England Biolabs, Ipswich, MA) following the manufacturer's protocol. All primer sequences used for PCR, except Taqman assays which were directly purchased from Applied Biosystems, are listed in Supplementary Table 3.

Western blot
Cell pellets or tissue fragments were lysed into RIPA buffer containing protein inhibitors. Lysis was augmented with sonication and the supernatant was cleared by centrifugation at 20,000g for 10 minutes. Protein concentration was measured by Bradford reagent assay. Ten µg of whole cell lysate were loaded for blotting.
Primary antibodies are listed in Supplementary Table S4.

Cell cultures
Glioma Stem-Like Cells (GSCs) G34 and G62 were previously obtained from primary human GBM specimens in Dr Chiocca's laboratory. MGG4 GSCs were kindly provided by Hiroaki Wakimoto from Massachusetts General Hospital. Neural Progenitor Cells were purchased form Life Technologies (cat # A15654) and were cultured as neurospheres using the same stem cell medium as described above. For GSCs and NPCs, neurobasal medium (Gibco/Life Technologies, MA, USA) supplemented with 2 µmol/L L-glutamine (Gibco), 20 ng/mL of epidermal growth factor (PeproTech, Rocky Hill, NJ), 20 ng/mL of basic fibroblast growth factor 2 (PeproTech), and 1× B27 supplement (Life Technologies) were used. The human glioblastoma cell lines, U251-

MG, U87-MG and T98G-MG (American Type Culture Collection), were grown in DMEM (Gibco) supplemented with 10% FBS (Gibco). For differentiation experiments, the cells were grown as monolayers in plastic dishes pre-coated with Poly-D-Lysine (Sigma-Aldrich, St Louis, MO). Neuronal differentiation was induced by removal of growth factors and addition of B27 medium containing Retinoic Acid (Gibco). Astrocytic differentiation was induced by changing the culture medium to DMEM supplemented with 10% Fetal Bovine Serum (FBS).

Protein knockdown

SiRNA oligonucleotides against EZH2, BMI1, LSD1 and RAB27A as well as negative control (Qiagen, Valencia, CA) were transfected at a 100 pmol / $5x10^5$ cells concentration and processed after 72 hours to validate their effect at the mRNA and protein levels. For double and triple knockdown studies, the combined concentration of siRNA oligonucleotides was maintained the same at 100 pmol / $5x10^5$ cells. All transfections were performed with Lipofectamine-2000 (Life Technologies) following the manufacturer's recommendations.

Transient Plasmid Transfection into GBM cells

For transient transfections, Cluster 3 and control DNA plasmids were used at a concentration of 5 µg/ml and mixed with 8 µl of Lipofectamine2000 and incubated with 2 $x10^5$ G34 cells in a conical tube (total volume 500 µl) for 6 hours at 37 degrees. After the incubation period, the cells were washed twice with PBS, plated in a 6-well plate and incubated for a period of 5 days in a transwell chamber (Greiner Bio-One, Monroe, NC) at 37 degrees.

Virus particles production

The lentiviral vector pCDH-EF1-copGFP (System Biosciences, Palo Alto, CA) was used to produce lentiviruses in HEK293 cells using the ViraPower packaging system (Invitrogen, Carlsbad, CA). Lipofectamine 2000 (Life Technologies) was used to transfect DNA plasmids into HEK293 cells. Supernatant was collected 60 hours after transfection and viral particles were concentrated by ultracentrifugation at 100,000 g for 90 minutes and resuspended in PBS.

Flow cytometry analysis

The U251 cells after siRNA knockdown and with either TMZ or RT exposure were washed twice with cold PBS containing 1% BSA, and then suspended with 100 µl Annexin V Binding Buffer. Then, 5 µl of APC/Annexin V, 5 µl of 7-AAD Viability Staining Solution (Annexin A5 Apoptosis Detection Kit, Biolegend, San Diego, CA) were added and incubated for 15 minutes in dark.

G34 GSC stably expressing microRNAs were washed twice with cold PBS and the samples were prepared using GFP-certified Apoptosis/Necrosis detection kit (Enzo Life Sciences, Farmingdale, NY), according to the instructions of the manufacturer protocol. The stained cells were immediately analysed with a BD Accuri C6 flow cytometer (Becton Dickinson, Franklin Lakes, NJ). Data analysis was performed using FlowJo Software (Tree Star Inc. Ashland, Oregon).

Electron Microscopy and Nanosight Analysis

The exosomes isolated as above were processed at the Harvard Medical School Electron Microscopy Facility and analyzed using a JEOL 1200EX transmission electron microscope. The images were recorded with an AMT 2k CCD camera. Immunogold labelling of the exosomes with CD63 and CD9 was performed by the facility personnel using their standard antibodies and protocols.

The conditioned media were collected from G34 parent cells as well as G34 cells stably expressing negative control and Cluster 3 transgenes, diluted 1:10 with double filtered PBS, and analyzed using NanoSight LM 10 (Malvern Instruments, Salisbury, UK), and quantified by NTA 3.0 program at the Nanosight Nanoparticle Sizing and Quantification Facility at the Massachusetts General Hospital, Boston, USA.

ATP-Glo Assay

Cells were plated in 96-well plates at a density of 2,000 cells/well in 200uL of culture medium. Cultures were treated with TMZ or 2Gy irradiation, and measurement of cell viability was performed 48 hours after treatment using the CellTiter-Glo kit (Promega) and the Polar Star Omega cell plate reader (BMG LabTech), according to the manufacturer’s instructions.
