# Supplementary material for: The functional synergism of microRNA clustering provides therapeutically relevant epigenetic interference in glioblastoma
Source: Nat Commun. 2019 Jan 25;10:442. doi: 10.1038/s41467-019-08390-z (PMC6347618; doi:10.1038/s41467-019-08390-z)
Supplement: Supplementary file 3 — Description of Additional Supplementary Files [file 41467_2019_8390_MOESM3_ESM.pdf]

Description of Additional Supplementary Files

File Name: Supplementary Data 1
Description: MicroRNA expression in GBM vs Brain. List of all annotated microRNA whose difference between GBM and brain specimen is significant at $p<0.05$. Differential expression is reported as Log Fold change, and relative p value of the difference is reported for each microRNA.

File Name: Supplementary Data 2
Description: List of conserved predicted mRNA targets for miR-124 by analysis with targetscan.org

File Name: Supplementary Data 3
Description: List of conserved predicted mRNA targets for miR-128 by analysis with targetscan.org

File Name: Supplementary Data 4
Description: List of conserved predicted mRNA targets for miR-137 by analysis with targetscan.org

File Name: Supplementary Data 5
Description: Column A reports all the genes found in Histone Modification category GO:001657. Columns B reports all the genes found in Negative Transcriptional Regulation category GO:0000122. Column C reports all genes in common betweento the two categories.

File Name: Supplementary Data 6
Description: Venn analysis crossing microRNA predicted targets with list of chromatin repressors.
