# Supplementary material for: The functional synergism of microRNA clustering provides therapeutically relevant epigenetic interference in glioblastoma
Source: Nat Commun. 2019 Jan 25;10:442. doi: 10.1038/s41467-019-08390-z (PMC6347618; doi:10.1038/s41467-019-08390-z)
Supplement: Supplementary file 10 — Reporting Summary [file 41467_2019_8390_MOESM10_ESM.pdf]

Editorial Note: Parts of this Peer Review File have been redacted as indicated to maintain the confidentiality of unpublished data and to remove third-party material where no permission to publish could be obtained.

Reviewers' comments:

Reviewer #1 (Remarks to the Author):

Very strong paper with impressive results using miR-123/128/137 as a therapeutic startegy; and extensive incorporation of primary tumors; and demonstration of bystander effects. Major weakness is the writing, or at least paragraph size. Each voluminous paragraph should be made into 2 or maybe 4. Survival data very impressive though, and mechanistic experiments well described.

Data in Fig 2 suggest some reciprocal inverse regulation of BMI1, EZH2 and LSD1, wherein knockdown of one led to upregulation of others. Any insights into how this occurs? Do levels of corresponding microRNAs change? When you knockdown BMI and EZH2 together in Fig 2 K, what happens to LSD1?

Any insights into how cluster 3 regulates myc nad dnmt1? Why does expression of cluster 3 decrease over time?

Reviewer #2 (Remarks to the Author):

In this manuscript, Bhaskaran et al found the cluster of microRNAs which significantly deregulated in GBM, and they have shown that re-expression of these microRNA suppressed their target genes, resulting in the contribution of survival of mouse with GBM. Furthermore, and this might be the most important point of this manuscript, authors found that these microRNA clusters are expanded by extracellular vesicles (EVs), resulting in the extension of their biological effect throughout the whole brain. The topic of this manuscript in interesting, however, I have several concerns which are important to insist their claims in this manuscript.

1. It is not clear that the expression of the microRNA cluster and the target genes are really from tumor cells or not. Thus authors should perform immunostaining of target genes and in situ hybridization of microRNA cluster in patient specimens.

2. On page 6 line 195, the author didn't show the data regarding the survival of the cells after the re-challenge of TMZ or RT treatment. Authors should show this to support their findings.

3. It has been shown that over-expression of vector-based microRNA inhibits the proper activity of Drosha and/or Dicer. Thus employing the GFP transgene as a control is not adequate. Authors should use the randomize sequence from polycistronic miRNA sequence as a control of miR-124/128/137 polycistronic miRNAs

4. In Figure 3H, the authors explained the reason for tumor progression after day 12 is because of the progressive loss of microRNA cluster expression. However, the author has used lentivirus to integrate this clustered microRNA. Thus how this microRNA can be a loss even integrated into the genome by lentivirus? If so, authors should show the re-expression of this clustered microRNA by injecting lentivirus with clustered microRNA to the tumor directly and see the survival of mice.

5. One largest concern about this manuscript is that how they can eliminate the possibility of transfer the microRNA cluster by lentivirus contamination instead of extracellular vesicles. In another word, I believe that what they called, bystander tumor cells, was not because of the extracellular vesicles transfer of microRNA, but because of the contamination of lentivirus with microRNA cluster. Only the way to avoid this is trying to establish the microRNA over-expression without lentivirus and did the same experiment shown in Figure 5 and 6. Indeed authors used the

same speed of ultracentrifugation when collecting lentivirus and collecting extracellular vesicles. Thus, their preparation of EVs could not eliminate the possibility of lentivirus contamination.

6. In Figure 5B, when authors compared the expression of microRNAs in the EVs, how they normalized each sample? Number of EVs? The protein concentration of EV fraction? Or total RNA from EVs? Authors should explain this clearly.

7. Regarding the query shown above, there is no single data showing the quality and character of EVs they have collected. Authors should show the marker of EVs, such as tetraspanin family proteins, endosomal proteins, and so on. In addition, the morphology of EVs taken by electron microscope should be shown. Further, and most importantly, the size and number of EVs from their cancer cell lines should be shown, especially with or without transduced microRNA cluster. Furthermore, to exclude the contamination of lentivirus, authors should perform the western blotting against virus protein to exclude the contamination of lentivirus in their preparation of EVs.

8. The mixture of cells, which they have used in this manuscript to prove the transfer of miRNA by EVs, could not prove the effect of EVs in vivo. If authors would like to insist the bystander effect by EVs, then the easiest way to prove this is that injection of EVs directly into an inoculated tumor. It is also important to eliminate the possibility of contamination of lentivirus in their preparation of EVs.

9. The best way to prove the involvement of EVs between cells is knock-down the genes which associated with EV secretion from cells. Those genes, including RAB27A, RAB27B, and nSMASE2, should be eliminated from the cells, which provide EVs, then assessed the transfer of microRNA as well as down-regulation of target genes.

10. Authors haven’t excluded the possibility of upregulation of endogenous microRNA cluster after the addition of EVs. Thus, authors should show the expression of primary microRNA after the addition of EVs.

We would like to thank the reviewers for taking the time to review our manuscript and for providing their excellent comments and insights. Please find below a point-by-point response to your comments. Additional cited references (in brackets) are reported at the end of the document.

Reviewer #1 (Remarks to the Author):

Very strong paper with impressive results using miR-123/128/137 as a therapeutic strategy; and extensive incorporation of primary tumors; and demonstration of bystander effects. Survival data very impressive though, and mechanistic experiments well described.

- Comment #1: Major weakness is the writing, or at least paragraph size. Each voluminous paragraph should be made into 2 or maybe 4.

  Answer: Thank you very much for your comments. We agree that the article might result fatiguing to read, as it touches upon several aspects at once. It was our intention to intertwingle purely mechanistic biological considerations of microRNAs clustering and regulation of epigenetic proteins in glioblastoma, with their translational relevance. Where possible, we have reduced wording and rearranged paragraphs to improve readability. Will be glad to further edit as advised by editor.

- Comment #2: Data in Fig 2 suggest some reciprocal inverse regulation of BMI1, EZH2 and LSD1, wherein knockdown of one led to upregulation of others. Any insights into how this occurs?

  Answer: We have performed RT-qPCR analyzing the expression of genes encoding BMI1, EZH2 and LSD1 (KDM1A) upon single knockdown by siRNA, to establish whether the observed upregulation of the other proteins (as seen in Fig 2J) is due to transcriptional activation, or might be due to other causes. We have observed a clear UP-regulation of mRNA expression of the non-targeted genes, suggesting that each one of them works as a negative transcriptional regulator for the other two, as shown below. These data have been added as Supplementary Figure S7 and discussed in the manuscript.

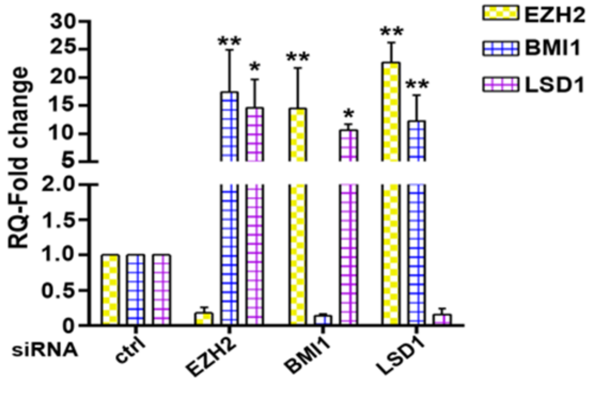

Supplementary Figure S7: Rebound transcriptional activation upon selective knock down. RT-qPCR expression analysis from Glioblastoma G34 cells after siRNA-mediated single knock

down of EZH2, BMI1 and LSD1. Results are from triplicate experiments. *=p<0.05, **=p<0.01 by two tailed t-test.

- Comment #3: Do levels of corresponding microRNAs change?

  Answer: This is an important question as it establishes whether the microRNAs controlling these proteins are, in return, also controlled by them. We have observed no significant changes in the expression of the three microRNAs upon downregulation of their respective target genes, as shown below. These data have been added to Figure 2.

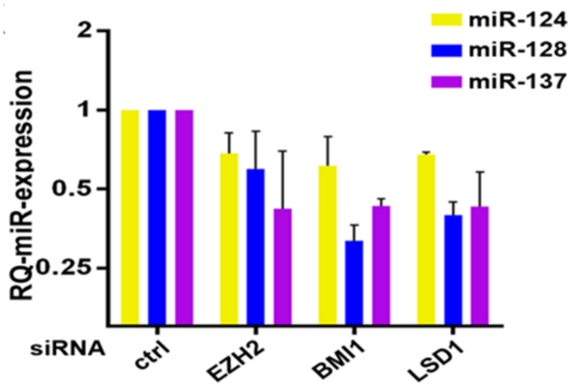

RT-qPCR expression analysis of miR-124, miR-128 and miR-137 from G34 Glioblastoma cells after single knock down of EZH1, BMI1 and LSD1. Reported are mean values from 3 experiments.

- Comment #4: When you knockdown BMI and EZH2 together in Fig 2 K, what happens to LSD1?

  Answer: We have performed both RT-qPCR and Western blot for EZH2, BMI1 and LSD1 after the three possible combinations of double knock-down (EZH2+BMI1, EZH2+LSD1 and BMI1+LSD1). In all three cases, the third element of the triad becomes overexpressed, both at the protein level and at the mRNA level, as shown below. These data have been added as Supplementary Figure S8 and discussed in the manuscript.

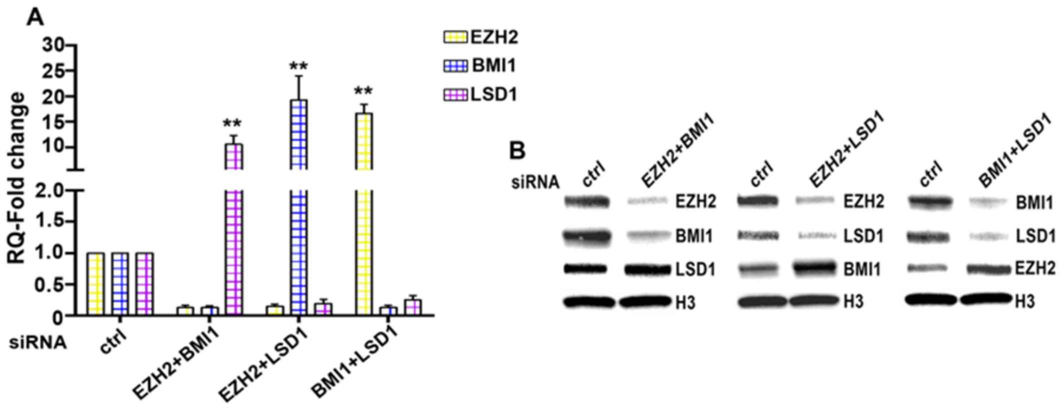

Supplementary Figure S8: Effect of double knock down A: RT-qPCR showing expression of EZH2, BMI1, and LSD1 after different combinations of double knock down in G34 cells. **B:**

Corresponding Western blot from protein lysate obtained from the same cells in panel A. Results are from triplicate experiments. **=p<0.01 by two tailed Student's t-test.

- Comment #5: Any insights into how cluster 3 regulates myc and dnmt1?

  Answer: According to our data, none of the three microRNAs has a direct effect on the expression level of the two proteins (Figure 3C). We do observe, though, a progressive downregulation of DNMT1 and MYC at the transcriptional level upon combined knock down of the EZH2/BMI1/LSD1 triad, suggesting that the combined suppression of the three epigenetic proteins (as it happens during Cluster 3 expression) exerts a progressively stronger interference on the transcriptional activation of MYC and DNMT1.
  These data have been added as Supplementary Figure S10 and discussed in the manuscript.

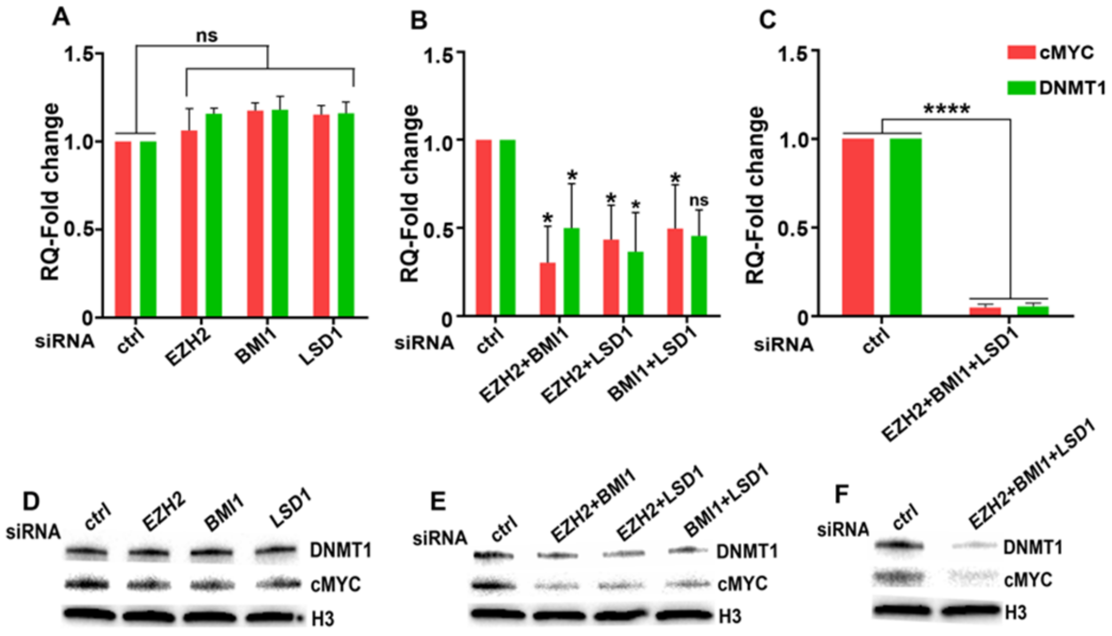

Supplementary Figure S10: DNMT1 and MYC expression incrementally declines upon progressive knockdown of EZH2, BMI1 and LSD1 function. RT-qPCR expression analysis of MYC and DNMT1 genes upon single (**A**), double (**B**) or triple knock down (**C**) of EZH2, BMI1 and LSD1 by siRNA transfection in G34 glioblastoma cells. Corresponding Western blots showing MYC and DNMT1 protein levels are presented in panels **D-F.** Represented data are means from triplicate experiments. *=p<0.05; ****=p<0.0001 by two tailed Student's t-test. ns= not significant.

- Comment #6: Why does expression of cluster 3 decrease over time?

  Answer: This is an interesting observation, which we agree was important to investigate and clarify. An important consideration to make and which was not stressed enough in our original manuscript, regards the genetic structure of the lentiviral vector backbone used for our overexpression experiments: briefly, this vector (pCDH) is constituted by a reporter GFP transgene which is under the control of the EF1 promoter, while the multiple cloning site, where the microRNA sequence is inserted, is controlled by an independent human CMV promoter sequence (see cartoon below). There is evidence in the literature that the CMV

promoter can become inactivated early on in vivo (Loser P et al., 1998), so we set up to investigate whether this could explain the observed progressive decrease of mature transgenic microRNA expression from tumor cells implanted intracranially.
The RNA of GBM cells stably transfected with Cluster 3 transgene or control vector, recovered from the brain after mice euthanasia, and sorted by GFP expression, was first treated with DNAse to remove any genomic contamination from the integrated transgenic DNA. Next, RT-qPCR was performed, measuring the levels of mature microRNAs (as originally shown in Supplementary Figure S7) and then we proceeded quantifying the expression level of the GFP transgene and the level of the Cluster 3 primary transcript (i.e. before microRNA processing).
As it is shown below, while GFP expression remains constant from day 1 through day 25, both mature microRNAs and primary Cluster 3 transcript show progressive decrease over time, strongly suggesting that the observed decrease in mature microRNAs is most likely due to progressive inactivation of transgene expression and not due, for example, to an acquired changes in microRNA processing/maturation.   These data have been added as Supplementary Figure 12 and discussed in the manuscript.

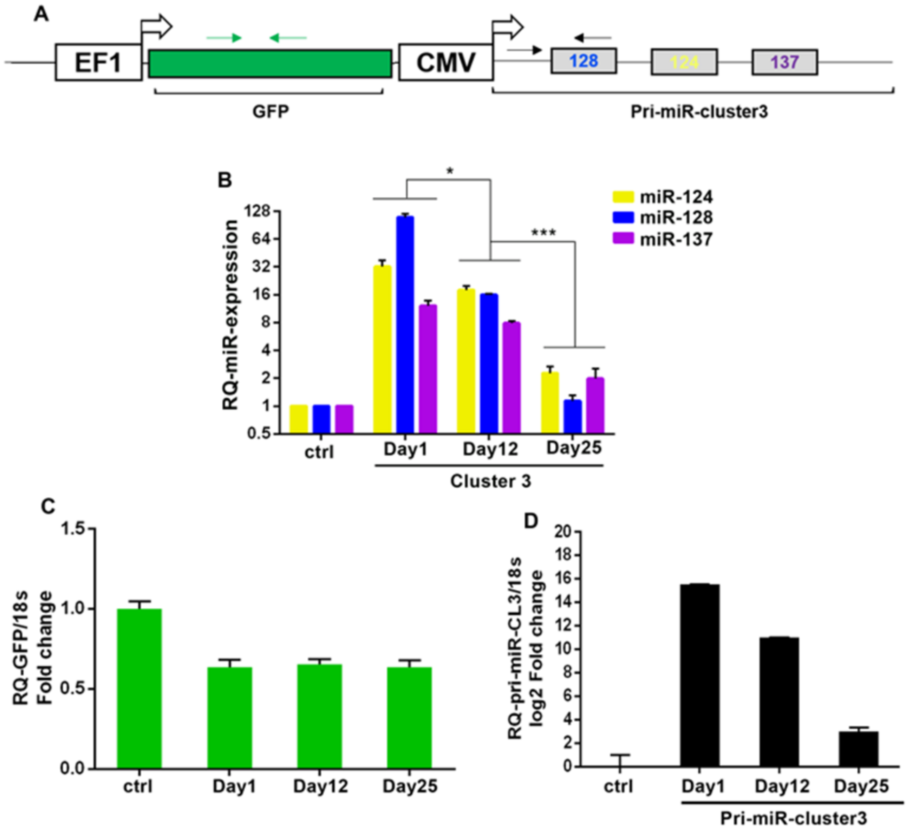

Supplementary Figure S12. Progressive loss of transgene expression *in vivo*. A: Cartoon schematizing the genomic configuration of the lentiviral vector used to overexpress microRNAs in this study. The GFP transgene is downstream to an EF1-responsive element, while the Cluster 3 primary transcript is downstream of the CMV promoter. Colored arrows represent primer sequences used for the RT-qPCR. **B:** Relative quantification of microRNA expression at different timepoints after intracranial implantation. G34 cells previously implanted intracranially in athymic mice were isolated from the brain at time of mouse

euthanasia (either at day 12 or at day 25) and the expression of cluster 3 transgene was measured against that of cells expressing negative control and against parental Cluster 3 cells at time of implantation. **C:** RT-qPCR showing expression of GFP transgene from cells in panel B. D: RT-qPCR showing expression of Primary Cluster 3 gene from cells in panel B. Reported are mean ± SD from three separate experiments. *=p<0.05; ***=p<0.001 (Student's t-test, 2 tails).

Reviewer #2 (Remarks to the Author):

In this manuscript, Bhaskaran et al found the cluster of microRNAs which significantly deregulated in GBM, and they have shown that re-expression of these microRNA suppressed their target genes, resulting in the contribution of survival of mouse with GBM. Furthermore, and this might be the most important point of this manuscript, authors found that these microRNA clusters are expanded by extracellular vesicles (EVs), resulting in the extension of their biological effect throughout the whole brain. The topic of this manuscript in interesting, however, I have several concerns which are important to insist their claims in this manuscript.

- Comment #1: It is not clear that the expression of the microRNA cluster and the target genes are really from tumor cells or not. Thus, authors should perform immunostaining of target genes and in situ hybridization of microRNA cluster in patient specimens.

  Answer: We thank the reviewer for the suggestion of performing simultaneous IHC for proteins and ISH for microRNAs. Below are representative images from IHC for EZH2, BMI1 and LSD1 in consecutive 5 micron FFPE slices comparing human GBM specimen to normal brain. The significant overexpression of each of the three proteins in the tumor is evident, as shown below. This also shows the obvious histological difference of the samples used in our experiments, suggesting that the samples used for the RNA analysis by PCR and Western blot were devoid of gross cross-contamination between the two tissues. This has been added as Supplementary Figure S3.

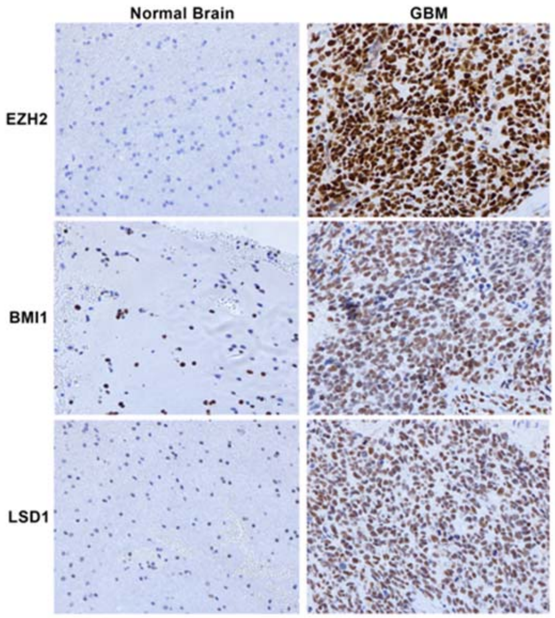

Supplementary Figure S3: Immunohistochemistry for EZH2, BMI1 and LSD1 in operative specimens of normal brain Vs glioblastoma. Cell nuclei are stained with Hematoxilin (blue) while target proteins are stained with DAB (brown). Magnification: 20x

Regretfully, in the given time we have not been able to successfully optimize the microRNA in situ hybridization procedure, as we were not able to obtain reliable stains with positive and negative control probes. We explored the option of outsourcing this investigation, but the requested fee for three microRNAs was in excess to $12,000, much higher than our budget.

In partial amend to this, we would like to cite here our prior work (Godlewski et al., 2008), where a co-stain for BMI1 and miR-128 was provided, showing an inverse correlation between the two, quantified in Panel C below.

[FIGURE REDACTED]

[Figure legend redacted.]

In addition, others have demonstrated by in situ hybridization that miR-124 is downregulated in primary brain tumors in comparison to normal brain (Nelson P et al., 2006).

We thus have not considered a priority to repeat these experiments, mainly because of the perceived high cost/benefit ratio. In fact, both RT-qPCR and high throughput analysis of bulk tissue are generally well accepted means to determine the relative abundance of microRNAs in different specimens, and there is consistent published evidence of the downregulation of each one of these three microRNAs in glioblastoma, as reported by many authors over the past decade (Godlewski et al., 2008; Singh S et al., 2012; Shea A et al., 2016; Ahir B et al., 2017).

We apologize for not being able to completely satisfy this request of the reviewer, but, if this is felt to be of the utmost importance for the acceptance of this work, we are willing to reconsider the cost to honor it.

- Comment #2: On page 6 line 195, the author didn't show the data regarding the survival of the cells after the re-challenge of TMZ or RT treatment. Authors should show this to support their findings.

Answer: We have added Supplementary Figure S6 (also displayed below) showing the lack of antiproliferative effect of Temozolomide or irradiation re-challenge in the three different cell lines reported in the main text and figures, after they had developed resistance per the protocol detailed in Figure 2D and 2E.

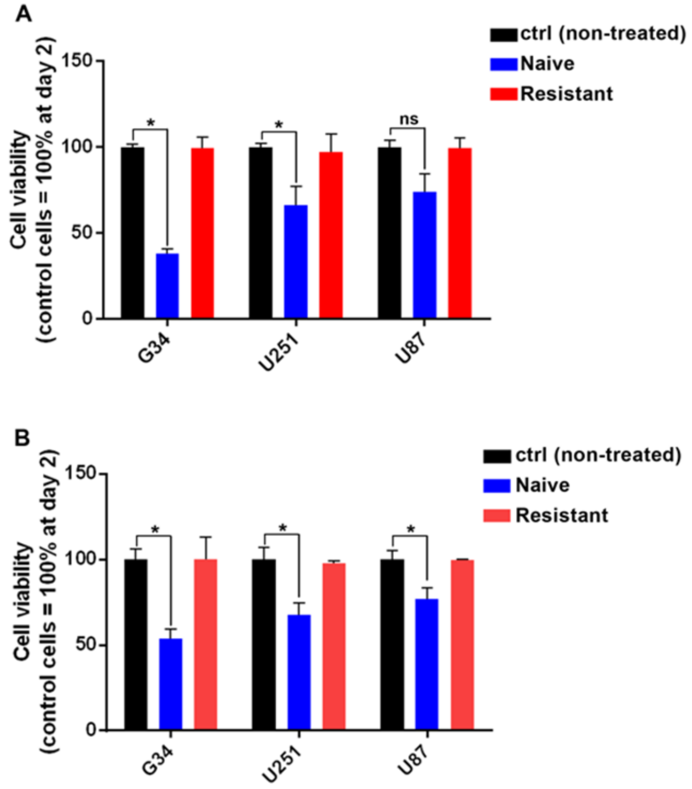

Supplementary Figure S6: Evidence of acquired resistance to Temozolomide and irradiation re-challenge after chronic exposure. Relative measurement of metabolic activity by ADP-Glo Kinase assay protocol 48 hours after treatment with 15 µM TMZ (panel A) or 2Gy irradiation (panel B). Blue bars represent cells that were naïve for prior treatment, while red bars represent cells which were previously treated and made resistant using the protocols described in the material and methods section and schematized in the cartoon in Figure 2D and 2E. Proliferation was normalized to untreated cells (black bars). Each experimental setting was performed in triplicates. *=$p<0.05$ by two tailed Student's t-test.

- Comment #3: It has been shown that over-expression of vector-based microRNA inhibits the proper activity of Drosha and/or Dicer. Thus employing the GFP transgene as a control is not adequate. Authors should use the randomize sequence from polycistronic miRNA sequence as a control of miR-124/128/137 polycistronic miRNAs.

Answer: The decision to use an “empty” vector (i.e. only expressing GFP) was made mainly because of the multiplicity of transgenes used in this work (single microRNA transgenes and cluster 3 transgene), making it difficult to decide what sequence would serve best as a negative control, and also because in our experience the use of “empty vectors” appeared to be well accepted in the literature.
Regardless, we agree with the reviewer that it is important to rule out the possibility that some unanticipated biological effects could be mediated by the presence of the transgene itself, independent of the production of mature microRNAs. Following this suggestion, we have designed a “scrambled control” sequence, by replacing the 20 nucleotide sequence encoding each mature microRNAs of Cluster 3 with a computer-generated, random sequence containing the exact number of nucleotides and same GC ratio of the native sequences, to minimize the amount of changes from the parental Cluster 3 sequence. We have then cloned the new scrambled sequence into our pCDH lentivector backbone, made stable G34 cell lines and repeated key experiments comparing the biological properties of cells transduced with either the empty vector, the scrambled control, or Cluster 3. Data presented below summarizes the evidence that the scrambled sequence did not show any significant biological difference compared to the empty vector control previously used, in terms of microRNA expression, effect on target proteins, effect on cell proliferation and sensitization to Temozolomide.

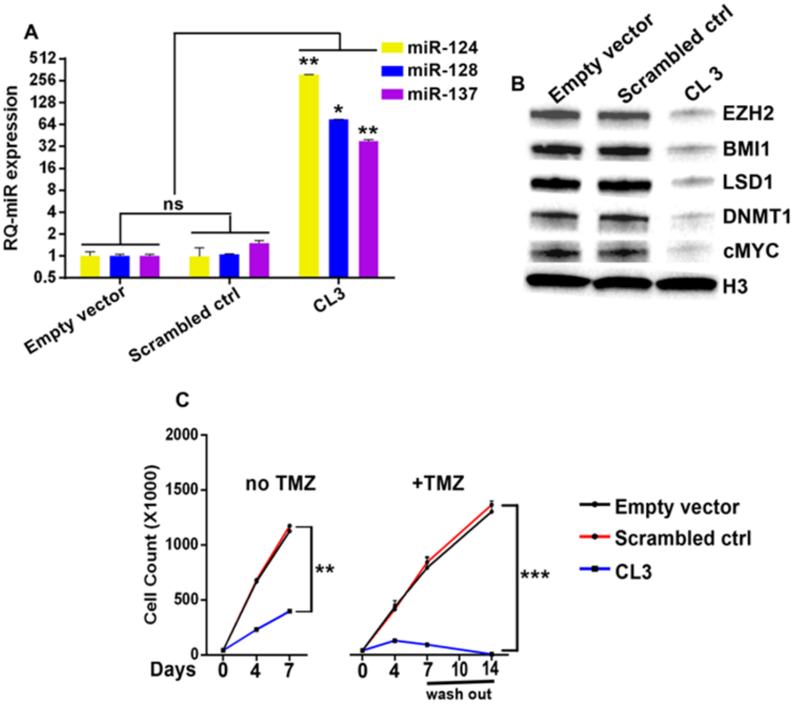

A: RT-qPCR showing the relative expression of mature miR-124, miR-128 and miR-137 from G34 glioma stem like cells after different lentivector transduction and FACS. “Empty Vector” refers to lentivector only expressing GFP; “Scrambled ctrl” refers to lentivector carrying GFP and an 800 bp sequence derived from Cluster 3, but with random modification of the three 20 nucleotide sequences encoding for miR-124, miR-128 and miR-137, respectively. “CL3” refers to the lentivector carrying GFP and the Cluster 3 sequence. **B:** Western blot analysis form protein lysate of cells in panel A. **C:** Cell count at different time points of G34 cells expressing the three different transgenes, both in the absence (left graph) and in the presence of 15

µM TMZ (right graph). Reported are means from triplicate experiments. *=p<0.05, **=p<0.01, by two tailed Student's t-test.

- Comment #4: In Figure 3H, the authors explained the reason for tumor progression after day 12 is because of the progressive loss of microRNA cluster expression. However, the author has used lentivirus to integrate this clustered microRNA. Thus how this microRNA can be a loss even integrated into the genome by lentivirus? If so, authors should show the re-expression of this clustered microRNA by injecting lentivirus with clustered microRNA to the tumor directly and see the survival of mice.

  Answer: Please refer to the response to Comment # 6 of Reviewer 1.

  The scope of this work was mainly to show the biological rationale and translational potential for the use of clustered microRNAs for glioblastoma therapy, a task well suited for lentiviral vectors, which, however, are not probably the best vectors for in vivo delivery, at least in our hands. In fact, we have attempted pilot experiments of intracranial injections with our lentivectors, but we have had very limited infectivity (as determined by amount of GFP-positive cells 5 days after infection). We have already started to use [redacted] as a more promising *in-vivo* carrier for our Cluster 3 transgene, and we have obtained [redacted] and significant extension of survival in mice, as shown below. [redacted].

[FIGURE REDACTED]

[Figure legend redacted.]

- <u>Comment #5:</u> One largest concern about this manuscript is that how they can eliminate the possibility of transfer the microRNA cluster by lentivirus contamination instead of extracellular vesicles. In another word, I believe that what they called, bystander tumor cells, was not because of the extracellular vesicles transfer of microRNA, but because of the contamination of lentivirus with microRNA cluster. Only the way to avoid this is trying to establish the microRNA over-expression without lentivirus and did the same experiment shown in Figure 5 and 6. Indeed authors used the same speed of ultracentrifugation when collecting lentivirus and collecting extracellular vesicles. Thus, their preparation of EVs could not eliminate the possibility of lentivirus contamination.

<u>Answer:</u> This is an interesting comment which brings about a few aspects that deserved further investigation on our part. The reviewer is correct when notices that the speed of ultracentrifugation is the same for both EVs and viruses, carrying the risk of concentrating virions together with the EVs used for downstream experiments.
Our reasoning for considering our experiments (and their results) appropriate, was based on two considerations:

1. The glioblastoma cells used as “EV producers” are cells which have been infected by our replication-defective lentivector and have been through a one week period of expansion, followed by FACS (by GFP expression), followed by another 1 week expansion before being used for experiments, including collection of EVs. We considered fairly safe to assume that within this 2 week period, any free virions from the initial infection would have been internalized by the cells. Also, since these vectors are unable to replicate, due to their defective genome, these stable cells are not able to produce new virions.
2. We have evidence (Figure 6B and Supplementary Figure 14D) that RFP cells growing together with GFP cells (i.e. those previously infected by the lentivector) do not express neither the Cluster 3 transgene nor the GFP transgene, but only the mature microRNAs, excluding the possibility that they have been infected by the GFP virion.

Notwithstanding, we considered the reviewer’s criticism well taken, and agreed with the suggestion provided by the reviewer to reproduce the experiment without using a virus. To do so, we only transfected G34 cells with the DNA plasmid encoding either Cluster 3 or scrambled control, but without helper plasmids (which are necessary for the packaging of virions in permissive cells), to be sure that those cells would not be able to produce any infectious virions. Yet, they would still be able to express the transgene. After transfection, these cells were rinsed twice in PBS and then placed in a transwell assay together with the usual

RFP cells, to repeat the experiments detailed in Figure 5. As shown below, even in this fashion, we observed an increase in the level of the three mature microRNAs in the RFP cells grown with the cells which were previously transfected with the Cluster 3 plasmid, and this resulted in a significant decrease in their proliferation, recapitulating what we had observed with the cells transfected by the virus. These data have been added as Supplementary Figure S16 and discussed in the manuscript.

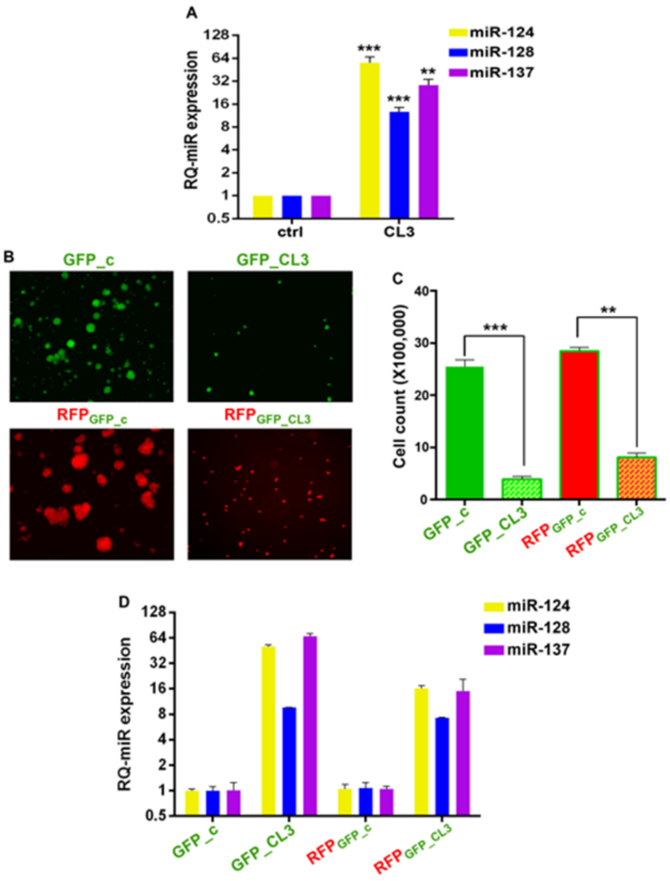

Supplementary Figure S16: microRNA upregulation in bystander cells is independent from lentivirus infection. Transwell assay performed with G34 cells transfected with DNA plasmids encoding either scrambled control or Cluster 3 transgenes but without the use of lentiviruses. **A:** RT-qPCR showing that transfected cells are able to produce mature microRNAs from the cluster 3 transgene. **B:** In a transwell assay, RFP-positive G34 cells growing together but separated from Cluster 3-transfected cells display delayed growth, quantified in panel **C**. **D:** RT-qPCR from cells in panel B, showing increased levels of mature miR-124, miR-128, and miR-137 in the RFP-positive cells growing in proximity of Cluster 3-transfected GFP cells. **=p<0.01; ***=p<0.001 by two tailed Student's t-test.

- Comment #6: Figure 5B, when authors compared the expression of microRNAs in the EVs, how they normalized each sample? Number of EVs? The protein concentration of EV fraction? Or total RNA from EVs? Authors should explain this clearly.

  Answer: The QuantiMir kit (System Biosciences, Palo Alto, CA) was used to extract total RNA from EVs. The RNA (10 ng per sample) was then retrotranscribed and used for PCR-based quantification of miR-124, miR-128 and

miR-137, using TaqMan primers (Life Technology). This clarification was added to the material and methods section of the manuscript.

- Comment #7: Regarding the query shown above, there is no single data showing the quality and character of EVs they have collected. Authors should show the marker of EVs, such as tetraspanin family proteins, endosomal proteins, and so on. In addition, the morphology of EVs taken by electron microscope should be shown. Further, and most importantly, the size and number of EVs from their cancer cell lines should be shown, especially with or without transduced microRNA cluster. Furthermore, to exclude the contamination of lentivirus, authors should perform the western blotting against virus protein to exclude the contamination of lentivirus in their preparation of EVs.

  Answer: Thank you for these pertinent comments. Below we provide the response to the specific requests. Our EV preparation appeared to be rather pure and homogeneous, without major differences in size or vesicles number due to the microRNA overexpression. Importantly, we could not find any evidence of virion contamination of our EV preparations, even by employing a 5 fold escalation of the Virus/EV ratio used in the experiments reported in the manuscript, as explained in the legend below. This data have been added as Supplementary Figure S17 and discussed in the main body of the manuscript.

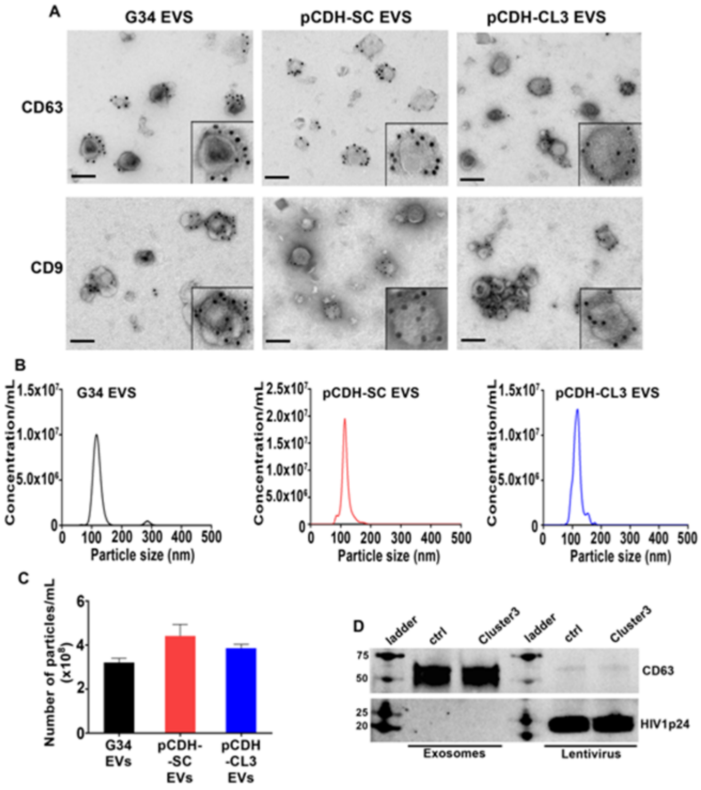

Supplementary Figure S17. Characterization of extracellular vesicles. A: Transmission Electron Microscopy images of representative EV preparations from either parental non infected, scrambled control and cluster 3-infected G34 cells. Immunolabeling for tetraspanins CD63 and CD9 was performed to show the enrichment of EVs markers. Bar=100 nm. Each picture has a 4x magnification box detailing immunolabeling. **B:** Nanosight analysis of the three EV preparations shown in panel A, demonstrating similar vesicle size distribution and homogeneity. **C:** Concentration of EVs for each preparation. **D:** Western blot of EVs obtained from scrambled control

(ctrl) or Cluster 3-infected cells, in comparison to corresponding lentivirus preparations used to infect the cells 2 weeks prior (the amount of EVs used for this Western blot is 10 μg of total proteins per lane, which is the same amount used in the experiments described in the manuscript, while the amount of lentivirus loaded in each well is 1 μg of proteins, corresponding to 1/5 of the amount of virus used to initially transduce glioblastoma cells).

- Comment #8: The mixture of cells, which they have used in this manuscript to prove the transfer of miRNA by EVs, could not prove the effect of EVs in vivo. If authors would like to insist the bystander effect by EVs, then the easiest way to prove this is that injection of EVs directly into an inoculated tumor. It is also important to eliminate the possibility of contamination of lentivirus in their preparation of EVs.

  Answer: We have inoculated intracranially an equal amount of EVs (10 μg, by protein quantification) derived from G34 cells expressing either scrambled control or Cluster 3 as described above. The EVs were administered in two separate 5 μl injections 2 days apart, starting 5 days after initial intracranial inoculation of 10,000 parental G34 cells. We observed a significant increase in median survival in the order of 5 days in the mice receiving EVs derived from cells expressing Cluster 3 transgene. The survival benefit was not as pronounced as the survival observed with the direct intracranial cell mixing reported in Figure 6E and 6F, but this was not particularly surprising and we speculate that the episodic EV administration is not as effective as the more constant production and transfer of EVs that happens during cell co-implantation. These data have been added to Figure 5 and discussed in the main body of the manuscript.

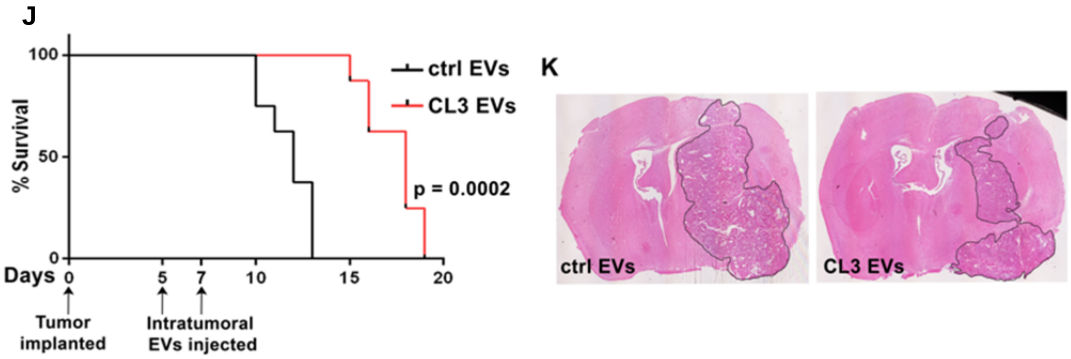

Kaplan-Meyer survival curve after intratumoral injection of purified EVs from G34 cells expressing either scrambled control or Cluster 3 transgene. Mice were inoculated at day 0 with 10,000 G34 glioma stem-like cells. Five days later, the mice were divided into 2 equal groups and were stereotactically inoculated intratumorally as described in panel **J**, with a total of 10 μg of EVs (by protein quantification), purified either from G34 cells expressing negative control transgene (ctrl EVs) or Cluster 3 transgene (CL3 EVs). Two mice per group were euthanized at the time the control group mice started showing signs of illness for histological analysis, shown in panel **K**.

- Comment #9: The best way to prove the involvement of EVs between cells is knock-down the genes which associated with EV secretion from cells. Those genes, including RAB27A, RAB27B, and nSMASE2, should be eliminated from the cells, which provide EVs, then assessed the transfer of microRNA as well as down-regulation of target genes.

Answer: Following the reviewer's suggestion, we performed RAB27A knock down by siRNA in our G34 cells transduced with either scrambled control or Cluster 3 transgenes, and, after verifying a significant decrease in the amount of EVs secretion by NanoSight analysis in comparison to cells treated with control siRNA, we repeated the transwell experiment described in Figure 5A-E. Importantly, knock down of RAB27A did not change the overall expression of mature microRNAs in the producing GFP-positive cells, nor it affected the growth of the cells, as compared to those treated with control siRNA. However, we did observe an almost complete loss of the inhibitory effect exerted by GFP-Cluster 3 cells on receiving RFP cells. RT-qPCR quantification of mature miR-124, miR-128 and miR-137 in those cells was not significantly different from the levels of cells exposed to control transgene, suggesting that the transfer of microRNAs was abolished by inhibiting EV release in GFP-positive cells. These data were added as Supplementary Figure S19 and discussed in the manuscript.

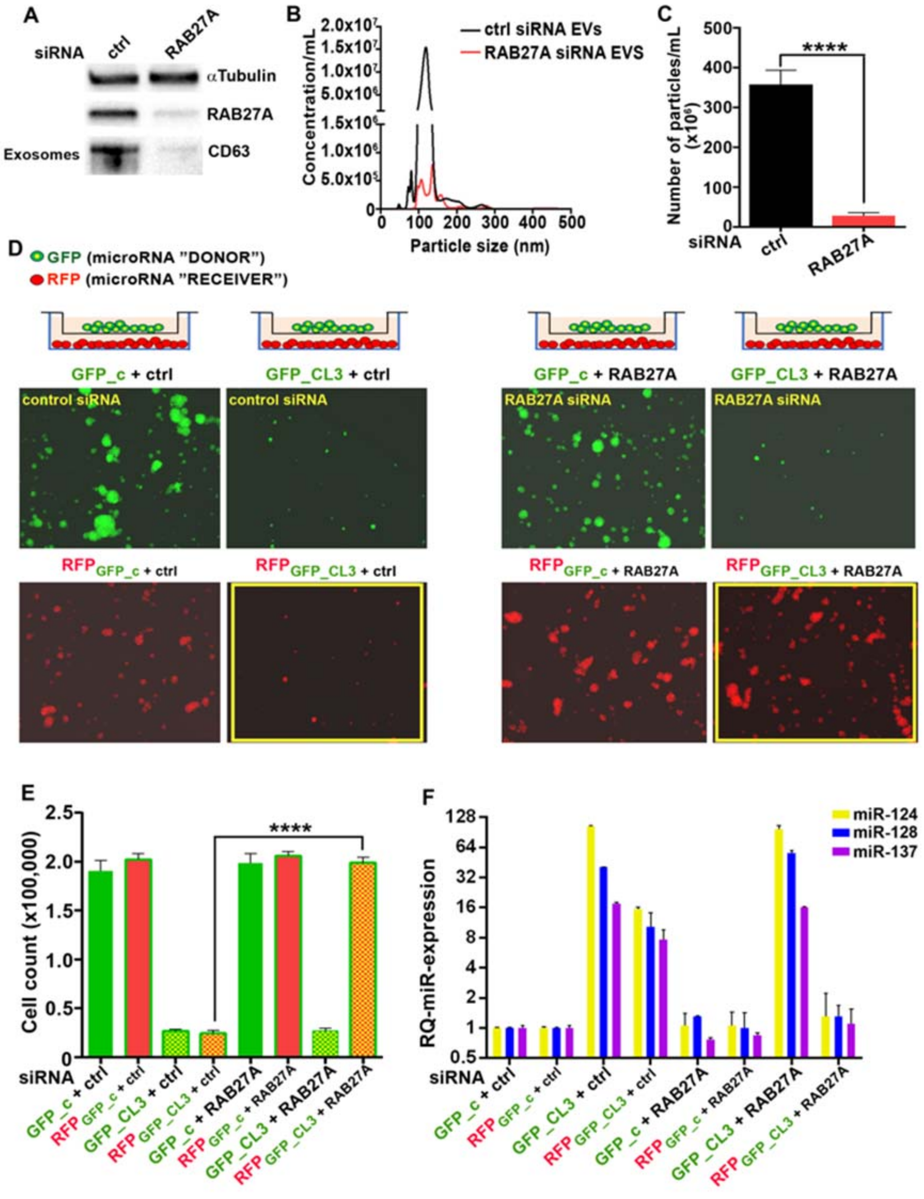

Supplementary Figure S19. Loss of microRNA transfer upon downregulation of the EVs secretory pathway. A: Western blot from G34 cells after treatment with RAB27A siRNA or negative control siRNA, showing strong RAB27A knockdown and resultant decrease in CD63 abundance in ultracentrifugation preparations. **B:** Nanosight analysis of size distribution of EVs obtained from cells in A. **C:** Nanosight quantification of EVs concentration of the two preparations. **D:** Transwell cultures of GFP-positive G34 cells stably expressing scrambled microRNA transgene (c) or Cluster 3 transgene (CL3), labelled in green, in the presence of control siRNA (ctrl) or RAB27A siRNA (RAB27A), labelled in black. Receiving RFP cells were not treated with any siRNAs. Each column represents a separate transwell assay. The 2 yellow frames underline the two samples whose statistical difference is reported in panel E. **E:** Cell counts of samples in D. **F:** RT-qPCR measuring level of mature microRNAs in the samples described in D. Experiments were performed in triplicates, except for microRNA qPCR, which was performed once. ***=p<0.001; ****=p<0.0001 by two tailed Student's t-test.

- Comment #10. Authors haven't excluded the possibility of upregulation of endogenous microRNA cluster after the addition of EVs. Thus, authors should show the expression of primary microRNA after the addition of EVs.

  Answer: Thank you for this very pertinent comment. As suggested, we have performed RT-qPCR to measure the expression of primary transcripts for miR-124-1, miR-128-1 and miR-137 in RFP-positive cells after they were treated with the EVs. As shown below, we did not observe any changes in the transcription levels of any of the three primary microRNAs, strengthening the possibility that the observed increase in mature microRNAs is indeed due to transfer from GFP cells rather than endogenous transcriptional activation in RFP cells. This data has been added as Supplementary Figure 18 and discussed in the manuscript.

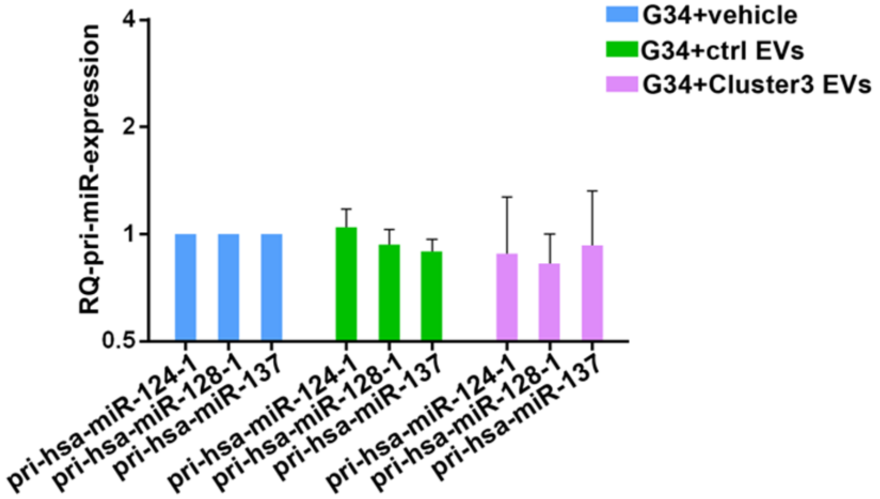

Supplementary Figure S18: EV administration does not induce expression of endogenous microRNA genes. RT-qPCR of primary microRNAs expression comparing the non treated G34 cells (G34+vehicle, blue bars) against those incubated with EVs derived either from negative control cells (green bars) or from Cluster 3 cells (pink bars). Reported data are from duplicate experiments.

References:

1. Ahir BK, Ozer H, Engelhard HH and Lakka SS. MicroRNAs in glioblastoma pathogenesis and therapy: a comprehensive review. Crit Rev Oncol Hematol. 2017;120: 22-33
2. Godlewski J, Nowicki MO, Bronisz A, Williams S, Otsuki A, Nuovo G, et al. Targeting of the Bmi-1 oncogene/stem cell renewal factor by microRNA-128 inhibits glioma proliferation and self-renewal. *Cancer Res*. 2008;68(22):9125-30
3. Loser P, Jennings GS, Strauss M and Sandig V. Reactivation of the previously silenced cytomegalovirus major immediate early promoter in the mouse liver: involvement of NFkB. J Virol. 1998; 72(1): 180-90
4. Shea A, Harish V, Afzal Z, Chijioke J, Kedir H, Dusmatova S et al. MicroRNAs in glioblastoma multiforme pathogenesis and therapeutics. *Cancer Med*. 2016;5(8):1917-46.
5. Singh SK, Vartanian A, Burrell K and Zadeh G. A microRNA link to glioblastoma heterogeneity. *Cancers*. 2012, 4, 846-872

REVIEWERS' COMMENTS:

Reviewer #1 (Remarks to the Author):

Revised manuscript adresses issues raised in prior review.

Reviewer #2 (Remarks to the Author):

The authors have answered all of my comments very honestly and precisely. Most importantly, what I have asked in the previous manuscript could be found in another a published elsewhere. In that aspect, this revised manuscript contains not only novel finding regarding the roles of microRNA in glioblastoma but also the variety of experiments which is extremely important for the EV research, which has been added in the revised manuscript based on what I have asked. I have nothing to comment on this manuscript further.
