# Supplementary material for: The functional synergism of microRNA clustering provides therapeutically relevant epigenetic interference in glioblastoma
Source: Nat Commun. 2019 Jan 25;10:442. doi: 10.1038/s41467-019-08390-z (PMC6347618; doi:10.1038/s41467-019-08390-z)
Supplement: Supplementary file 2 — Peer Review File [file 41467_2019_8390_MOESM2_ESM.pdf]

nature research

Corresponding author(s): Pierpaolo Peruzzi

Reporting Summary

Nature Research wishes to improve the reproducibility of the work that we publish. This form provides structure for consistency and transparency in reporting. For further information on Nature Research policies, see Authors & Referees and the Editorial Policy Checklist.

Statistical parameters

When statistical analyses are reported, confirm that the following items are present in the relevant location (e.g. figure legend, table legend, main text, or Methods section).

n/a	Confirmed	
☐	☒	The exact sample size (*n*) for each experimental group/condition, given as a discrete number and unit of measurement
☐	☒	An indication of whether measurements were taken from distinct samples or whether the same sample was measured repeatedly
☐	☒	The statistical test(s) used AND whether they are one- or two-sided<br>*Only common tests should be described solely by name; describe more complex techniques in the Methods section.*
☒	☐	A description of all covariates tested
☐	☒	A description of any assumptions or corrections, such as tests of normality and adjustment for multiple comparisons
☐	☒	A full description of the statistics including central tendency (e.g. means) or other basic estimates (e.g. regression coefficient) AND variation (e.g. standard deviation) or associated estimates of uncertainty (e.g. confidence intervals)
☐	☒	For null hypothesis testing, the test statistic (e.g. *F*, *t*, *r*) with confidence intervals, effect sizes, degrees of freedom and *P* value noted<br>*Give P values as exact values whenever suitable.*
☒	☐	For Bayesian analysis, information on the choice of priors and Markov chain Monte Carlo settings
☒	☐	For hierarchical and complex designs, identification of the appropriate level for tests and full reporting of outcomes
☒	☐	Estimates of effect sizes (e.g. Cohen's *d*, Pearson's *r*), indicating how they were calculated
☐	☒	Clearly defined error bars<br>*State explicitly what error bars represent (e.g. SD, SE, CI)*

Our web collection on statistics for biologists may be useful.

Software and code

Policy information about availability of computer code

Data collection	microRNA expression data in clinical specimens was obtained from publicly available TCGA database
Data analysis	Analysis of differential microRNA expression from TCGA data was performed using LIMMA R package<br>All other analysis were performed using GraphPad Prism, Version 6.0

For manuscripts utilizing custom algorithms or software that are central to the research but not yet described in published literature, software must be made available to editors/reviewers upon request. We strongly encourage code deposition in a community repository (e.g. GitHub). See the Nature Research guidelines for submitting code & software for further information.

Data

Policy information about availability of data

All manuscripts must include a data availability statement. This statement should provide the following information, where applicable:
- Accession codes, unique identifiers, or web links for publicly available datasets
- A list of figures that have associated raw data
- A description of any restrictions on data availability

The data that support the findings of this study which are not directly available within the paper [and its supplementary information files] will be available from the corresponding author upon reasonable request. This includes DNA sequences of all transgenes used in this study.

Field-specific reporting

Please select the best fit for your research. If you are not sure, read the appropriate sections before making your selection.

☒ Life sciences ☐ Behavioural & social sciences ☐ Ecological, evolutionary & environmental sciences

For a reference copy of the document with all sections, see nature.com/authors/policies/ReportingSummary-flat.pdf

Life sciences study design

All studies must disclose on these points even when the disclosure is negative.

Sample size	TCGA sample size was made of 558 glioblastomas and 10 normal brains.<br>All survival animal studies were performed with at least 6 animals per group
Data exclusions	no data were excluded from analysis
Replication	All experiments were replicated three times, unless otherwise specified in figure legends.
Randomization	randomization was performed for the in vivo EV injection, whereby animals were initially injected with the same amount of GBM cells and then, at time of EV injections, they were divided randomly into two equal groups of 6 animals, receiving control or microRNA EVs, respectively.
Blinding	Blinding was not possible in this study as the authors needed to be aware at any given time of the transgene or treatment applied to each sample.

Reporting for specific materials, systems and methods

Materials & experimental systems

n/a	Involved in the study	
☐	☒	Unique biological materials
☐	☒	Antibodies
☐	☒	Eukaryotic cell lines
☒	☐	Palaeontology
☐	☒	Animals and other organisms
☒	☐	Human research participants

Methods

n/a	Involved in the study	
☒	☐	ChIP-seq
☐	☒	Flow cytometry
☒	☐	MRI-based neuroimaging

Unique biological materials

Policy information about availability of materials

Obtaining unique materials	All unique biological material used in this study are available from the authors upon request.

Antibodies

Antibodies used	EZH2 (IHC) Cell Signaling Technology D2C9<br>BMI1 (IHC) Cell Signaling Technology D20B7<br>LSD1 (IHC) Cell Signaling Technology 2139S<br>EZH2 Cell Signaling Technology D2C9<br>BMI1 Cell Signaling Technology D42B3<br>LSD1 Cell Signaling Technology C69G12<br>(UTX) KDM6A Cell Signaling Technology D3Q1I<br>DNMT1 Cell Signaling Technology D63A6<br>cMYC Cell Signaling Technology D84C12<br>SP1 ABclonal Technology A3331<br>JAG1 ABclonal Technology A12733<br>p21 Waf1/Cip1 Cell Signaling Technology 12D1<br>Phospho-Histone H2A.X(Ser139) Cell Signaling Technology 20E3<br>Histone H2A.X Cell Signaling Technology 2595S<br>β3-Tubulin Cell Signaling Technology D71G9

GFAP Cell Signaling Technology D1F4Q
Nestin Santa Cruz Biotechnology SC-23927
α-Tubulin Sigma Aldrich T6074
Histone H3 Cell Signaling Technology D1H2
CD63 BD Pharmingen 556019
HIV1 p24 ThermoFisher Scientific N29 (05-005)

Validation	Each and every antibody used in this manuscript was tested and validated by the respective vendor for the specific application for which it was used in our experiments.

Eukaryotic cell lines

Policy information about cell lines

Cell line source(s)	G34 and G62 glioblastoma stem-like cells were previously isolated from primary glioblastoma specimens in our laboratory.<br>MGG4 glioblastoma stem-like cells were a gift of Dr Wakimoto from Massachusetts General Hospital.<br>U87, U251,T98G and CT2A cell lines were previoulsy purchased from ATCC and stored in our laboratory cell repository.<br>Human Neural Stem Cells were purchased form Thermo Fisher Scientific (Cat # A15654).
Authentication	G34 cells were analyzed and autenthicated as human by Idexx Bioresearch (www.idexbioresearch.com) using short tandem repeat profiling.
Mycoplasma contamination	G34 cells were tested for mycoplasma and resulted negative for contamination (analysis performed by third party at Yale University Laboratories)
Commonly misidentified lines (See ICLAC register)	none

Animals and other organisms

Policy information about studies involving animals; ARRIVE guidelines recommended for reporting animal research

Laboratory animals	Mouse Athymic nu/nu, Females, age 6-8 weeks. Purchased from Envigo (www.envigo.com)
Wild animals	This study did not involve wild animals
Field-collected samples	This study did not involve field-collected samples

Flow Cytometry

Plots

Confirm that:

☒ The axis labels state the marker and fluorochrome used (e.g. CD4-FITC).

☒ The axis scales are clearly visible. Include numbers along axes only for bottom left plot of group (a 'group' is an analysis of identical markers).

☒ All plots are contour plots with outliers or pseudocolor plots.

☒ A numerical value for number of cells or percentage (with statistics) is provided.

Methodology

Sample preparation	The U251 cells after siRNA knockdown and with either TMZ or RT exposure were washed twice with cold PBS containing 1% BSA, and then suspended with 100μl Annexin V Binding Buffer. Then, 5μl of APC/Annexin V, 5μl of 7-AAD Viability Staining Solution (Annexin A5 Apoptosis Detection Kit, Biolegend, San Diego, CA) were added and incubated for 15 minutes in dark.<br>G34 GSC stably expressing microRNAs were washed twice with cold PBS and the samples were prepared using GFP-certified Apoptosis/Necrosis detection kit (Enzo Life Sciences, Farmingdale, NY), according to the instructions of the manufacturer protocol.
Instrument	BD Accuri C6 flow cytometer (Becton Dickinson, Franklin Lakes, NJ)
Software	FlowJo Software (Tree Star Inc. Ashland, Oregon)
Cell population abundance	> 10,000 cells per experiment were analyzed
Gating strategy	Gating parameters were chosen so that they would group greater than 90% of the control sample cells. Gating parameters were kept identical across experimental samples.

☒ Tick this box to confirm that a figure exemplifying the gating strategy is provided in the Supplementary Information.
